# HAX1-dependent control of mitochondrial proteostasis governs neutrophil granulocyte differentiation

**DOI:** 10.1172/JCI153153

**Published:** 2022-05-02

**Authors:** Yanxin Fan, Marta Murgia, Monika I. Linder, Yoko Mizoguchi, Cong Wang, Marcin Łyszkiewicz, Natalia Ziȩtara, Yanshan Liu, Stephanie Frenz, Gabriela Sciuccati, Armando Partida-Gaytan, Zahra Alizadeh, Nima Rezaei, Peter Rehling, Sven Dennerlein, Matthias Mann, Christoph Klein

**Affiliations:** 1 Department of Pediatrics, Dr. von Hauner Children’s Hospital and Gene Center, University Hospital, Ludwig-Maximilians-Universität (LMU), Munich, Germany.; 2 Department of Proteomics and Signal Transduction, Max Planck Institute of Biochemistry, Martinsried, Germany.; 3 Department of Biomedical Sciences, University of Padua, Padua, Italy.; 4 Department of Cellular Biochemistry, University Medical Center Goettingen, Goettingen, Germany.; 5 Hematology and Oncology Department, Hospital de Pediatria “Prof. Dr. J.P. Garrahan,” Buenos Aires, Argentina.; 6 Unidad de Investigación en Inmunodeficiencias Primarias, Instituto Nacional de Pediatría, Mexico City, Mexico.; 7 Immunology, Asthma and Allergy Research Institute and; 8 Research Center for Immunodeficiencies, Children’s Medical Center, Tehran University of Medical Sciences, Tehran, Iran.; 9 Cluster of Excellence “Multiscale Bioimaging: From Molecular Machines to Networks of Excitable Cells,” University of Goettingen, Goettingen, Germany.; 10 Max Planck Institute for Biophysical Chemistry, Goettingen, Germany.

**Keywords:** Cell Biology, Immunology, Mitochondria, Neutrophils

## Abstract

The relevance of molecular mechanisms governing mitochondrial proteostasis to the differentiation and function of hematopoietic and immune cells is largely elusive. Through dissection of the network of proteins related to HCLS1-associated protein X-1, we defined a potentially novel functional CLPB/HAX1/(PRKD2)/HSP27 axis with critical importance for the differentiation of neutrophil granulocytes and, thus, elucidated molecular and metabolic mechanisms underlying congenital neutropenia in patients with HAX1 deficiency as well as bi- and monoallelic mutations in *CLPB*. As shown by stable isotope labeling by amino acids in cell culture (SILAC) proteomics, CLPB and HAX1 control the balance of mitochondrial protein synthesis and persistence crucial for proper mitochondrial function. Impaired mitochondrial protein dynamics are associated with decreased abundance of the serine-threonine kinase PRKD2 and HSP27 phosphorylated on serines 78 and 82. Cellular defects in HAX1^–/–^ cells can be functionally reconstituted by HSP27. Thus, mitochondrial proteostasis emerges as a critical molecular and metabolic mechanism governing the differentiation and function of neutrophil granulocytes.

## Introduction

Neutrophil granulocytes are critical mediators of innate immunity (1). In a highly controlled process of granulopoiesis, granulocyte-monocyte progenitors develop into mature neutrophils (2, 3). This developmental path is orchestrated by cytokine-dependent transcriptional networks and associated with remodeling of cell nucleus as well as granule formation (4–6). Besides this genetic control of differentiation, metabolism-dependent processes have been implicated in this highly coordinated differentiation process.

The autophagy-controlled fatty acid oxidation–oxidative (FAO-oxidative) phosphorylation (OXPHOS) pathway functions as a critical cellular mechanism to supply sufficient ATP for the energy-demanding neutrophil differentiation process (7). Besides this critical regulation of the energy-metabolic adaptation during hematopoiesis, mitochondria link metabolism-dependent gene regulation with proper hematopoietic stem cell function (8).

Mitochondria are well known for their role as biosynthetic and bioenergetic organelles and serve as central components for protein homeostasis (proteostasis) (9, 10). Mitochondrial proteostasis faces unique challenges as the majority of mitochondrial proteins are encoded by the nuclear genome and translated in the cytosol (11). These nuclear-encoded mitochondrial proteins need to be imported in their unfolded states to pass through import channels in mitochondrial membranes before being processed and assembled into their functional states (12–14). Moreover, several mitochondrial multiprotein complexes (4 of the OXPHOS complexes) are assembled from both nuclear and mitochondrial polypeptides. Mitochondria have evolved complex networks of molecular chaperones and proteolytic systems and other quality control factors to maintain a functionally competent mitochondrial proteome (11, 15, 16).

The conserved mitochondrial serine proteases Parl (presenilin-associated, rhomboid-like) and HtrA2 (high temperature–regulated A2; also known as Omi) interact with the antiapoptotic HCLS1-associated protein X-1 (HAX1) (17). It has been hypothesized that this protein network may allow Hax1 to present HtrA2 to Parl, thus facilitating cleavage of HtrA2 and preventing accumulation of mitochondrial outer membrane–associated activated Bax and apoptosis (17).

Consistent with its antiapoptotic role in hematopoietic cells, HAX1 deficiency in humans causes severe congenital neutropenia associated also with variable neurological impairment (18). Recently, HAX1 was shown to interact with the disaggregase CLPB, a member of the ATPases associated with diverse cellular activities (AAA+) (19). Notably, biallelic autosomal recessive mutations in *CLPB* have been linked to 3-methylglutaconic aciduria, a severe mitochondrial disorder associated with increased levels of 3-methylglutaconic acid, neurological impairment, and occasionally neutropenia (20, 21).

Recent studies have highlighted the importance of functional mitochondria in maintaining hematopoietic stem cell function (8) and mitochondrial metabolism for cellular differentiation (7, 22–25). However, the molecules and pathways governing the dynamics of mitochondrial protein/complex synthesis and persistence critical to preserve mitochondrial proteostasis required during neutrophil differentiation are largely undefined. Critical downstream pathway target proteins of this axis that act in concert to preserve mitochondrial functions remain largely elusive.

Here, we probe mitochondrial proteostatic networks in innate immunity and address our hypothesis that neutrophil granulocytes are particularly sensitive to disturbances of mitochondrial proteostasis. We define a critical CLPB/HAX1 axis for maintaining mitochondrial fitness and functionally link HAX1 to the PRKD2 kinase and the small heat shock protein HSP27.

## Results

### HAX1 is an interaction partner and substrate of CLPB in the mitochondrial intermembrane space.

**Figure 1:**
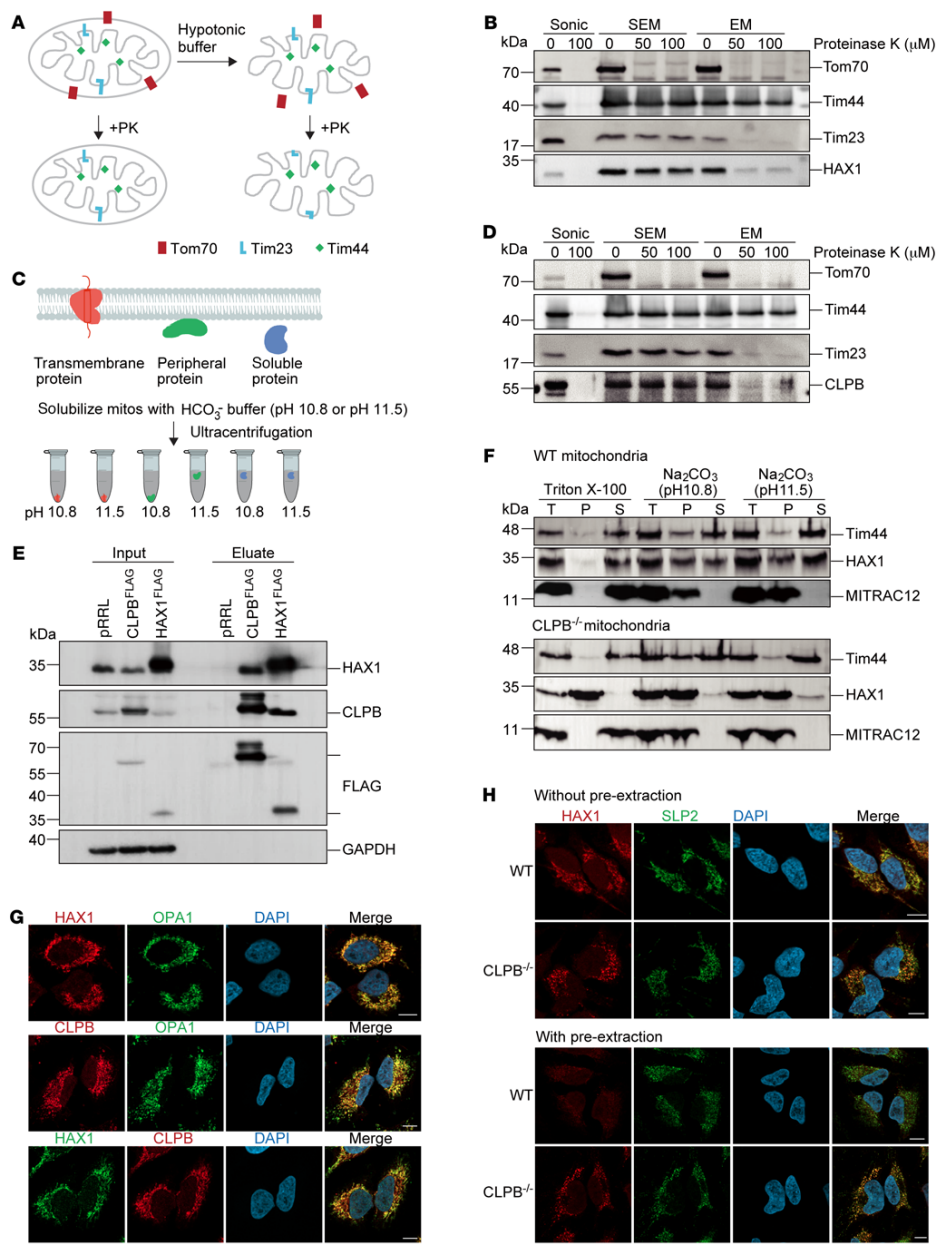
HAX1 is an interaction partner and a substrate of CLPB in the mitochondrial intermembrane space. (**A**) Scheme of mitochondrial swelling. (**B**) Submitochondrial localization of HAX1 analyzed by protease protection. Proteinase K (PK) was applied to sonicated mitochondria (Sonic), intact mitochondria (SEM), or mitoplasts (hypotonically swollen mitochondria by EM). (**C**) Scheme of carbonate extraction. (**D**) Submitochondrial localization of CLPB analyzed by protease protection. PK was applied to sonicated mitochondria (Sonic), intact mitochondria (SEM), or mitoplasts (hypotonically swollen mitochondria by EM). (**E**) Immunoprecipitation (IP) experiments with lysates from HEK293T cells expressing WT HAX1^FLAG^, CLPB^FLAG^, or empty pRRL vector. Proteins in the input (2%) and in the bound fractions (50%) were analyzed by immunoblotting. (**F**) Membrane association of HAX1 in WT or CLPB^–/–^ PLB-985 cells. Freshly isolated mitochondria were subjected to carbonate extraction or detergent lysis, separated into supernatant (S) and pellet (P) fractions, and analyzed by immunoblotting (T, total). See complete unedited blots in the supplemental material. (**G**) Confocal images of immunostaining of OPA1 and HAX1, OPA1 and CLPB, or HAX1 and CLPB in combination with DAPI (DNA staining) in HeLa cells. Scale bars: 10 μm. (**H**) Immunostaining of HAX1 and SLP2 in HeLa WT or CLPB^–/–^ cells without (top panel) or with (bottom panel) pre-extraction treatment. DNA was visualized with DAPI. Scale bars: 10 μm.

HAX1 was found to predominantly localize to mitochondria (26, 27), yet the exact distribution and function of HAX1 in promyeloid cells remain largely obscure. To study the intracellular localization of HAX1, we chose the promyelocytic leukemic PLB-985 cell line. Mitochondrial proteins are distributed into 4 different subcompartments: outer membrane, inner membrane, intermembrane space (IMS), and matrix (scheme in Figure 1A).

To define the spatial organization of HAX1 in mitochondria, we tested the accessibility of HAX1 to externally added proteinase K (PK) under iso- or hypo-osmotic buffer conditions (sucrose, EDTA, MOPS [SEM] or EDTA, MOPS [EM] buffer). While iso-osmotic conditions allow PK to only degrade outer membrane proteins accessible from the cytosolic side, hypo-osmotic conditions enable the protease to degrade protein domains in or exposed to the IMS (28).

Treatment of isolated mitochondria with PK led to the degradation of the outer membrane protein TOM70 (Figure 1B, lane 1), whereas TIM23, an IMS protein, remained intact under iso-osmotic conditions (Figure 1B, lane 3). Similarly to TIM23, HAX1 remained unaffected after PK treatment (Figure 1B, lane 4). Upon osmotic disruption of the outer mitochondrial membrane, TIM23 and HAX1 became accessible to PK treatment, indicating that both proteins were exposing their antibody binding regions to the IMS (Figure 1B, lanes 3 and 4).

Next, we examined whether HAX1 is a membrane protein and performed experiments involving carbonate extraction of isolated mitochondria (Figure 1C). TIM44, a peripherally membrane-associated protein (29), was released from the mitochondrial membrane into the supernatant (S) at pH 11.5 (Figure 1F, lane 1). Similarly, HAX1 partially remained in the pellet fraction (P) upon carbonate extraction at pH 11.5. Since HAX1 was partially resistant to carbonate extraction (Figure 1F, lane 2), we conclude that HAX1 is associated with the mitochondrial membrane.

HAX1 appears to regulate highly diverse cellular processes through binding to a multitude of interaction partners, yet the functional relevance of these networks is controversial (30). To gain insights into HAX1 protein networks at the IMS, we performed immunoprecipitation (IP) experiments. For this interaction analysis, we transfected FLAG-tagged HAX1 into HEK293T cells, highly accessible to transient transfection of DNA and capable of producing large amounts of recombinant protein, and determined the interacting factors of HAX1 by mass spectrometry (Supplemental Figure 1A; supplemental material available online with this article; https://doi.org/10.1172/JCI153153DS1). One of the most prominent binding partners of HAX1 was identified as caseinolytic peptidase B protein homolog (CLPB) (Supplemental Table 1). CLPB is a member of the ATPases associated with diverse cellular activities (AAA+) that serve critical functions in proteostasis by promoting disaggregation/solubilization and downstream refolding of substrates (31–34). In bacteria, ClpB confers resistance against heat shock (35) and regulates thermal stress responses (36) by threading unfolded polypeptides through the central channel of a hexamer ring (37). In humans, biallelic mutations in CLPB have been identified in children suffering from cataracts, neurodevelopmental defects, and congenital neutropenia (20, 21).

To verify our mass spectrometry data, the eluate of the HAX1-FLAG IP was analyzed by immunoblotting, confirming the enrichment of CLPB in the IP (Figure 1E). Importantly, we confirmed the binding between HAX1 and CLPB in the human myeloid cell line HL-60 (Supplemental Figure 2B). The bidirectional coimmunoprecipitation studies further confirmed the mutual interaction between HAX1 and CLPB (Figure 1E). The interaction between HAX1 and CLPB was also evidenced by an endogenous antibody IP (Supplemental Figure 1B).

We next analyzed the submitochondrial localization of CLPB (Figure 1D). Similarly to HAX1, CLPB remained intact upon PK treatment, whereas under hypo-osmotic buffer conditions both proteins were degraded. We next studied the localization of HAX1 and CLPB in HeLa cells by confocal microscopy. Interestingly, both HAX1 and CLPB were partially colocalized with the IMS-resident protein OPA1 (Figure 1G; for specificity of the HAX1 and CLPB staining, see Supplemental Figure 1C).

Intrigued by the partial overlap in the clinical manifestation in HAX1 and CLPB deficiency, we hypothesized that HAX1 is functionally downstream of CLPB and that the phenotype of congenital neutropenia in CLPB deficiency is mediated by defective function of HAX1. To address the question of whether the interaction with CLPB preserves the function of HAX1 by preventing misfolding and aggregation of HAX1, we generated a series of CLPB-knockout (KO) cell lines using the CRISPR/Cas9 system. Mutant isogenic clones were isolated and tested for protein expression (Supplemental Figure 2C). In mitochondria isolated from CLPB-KO cells, HAX1 was recovered mainly from the pellet fraction after either nonionic detergent lysis or carbonate extraction (Figure 1F, lane 5), indicating that it formed insoluble aggregates in the absence of CLPB. Importantly, the solubility of TIM44 was not affected by the absence of CLPB (Figure 1F, lane 4).

To assess how the absence of CLPB affects the cellular distribution of HAX1, we performed immunofluorescence studies. In control cells, HAX1 was uniformly distributed, while in the absence of CLPB, HAX1 was enriched in bright puncta (Figure 1H, top panel). Notably, pre-extraction of cells prior to fixation removed soluble HAX1 in control cells, while in CLPB-deficient cells, HAX1 remained partially colocalized with the mitochondrial matrix protein SLP2 (a mitochondrial protein that assembles into large SPY complexes in the inner membrane; ref. 38) (Figure 1H, bottom panel). Thus, CLPB ensures proper folding and correct distribution of HAX1 in the mitochondrial IMS.

### Phenotypes of patients with monoallelic variants in CLPB.

In our Care-for-Rare repository for inborn errors of immunity (refer to https://www.care-for-rare.org), we identified 4 patients with heterozygous variants in the *CLPB* gene (Table 1). Clinically, 3 of 4 patients (patients 1, 2, and 3) presented with severe neutropenia in the first year, and patient 4 presented with severe neutropenia in the seventh year of life leading to recurrent bacterial infections. Examination of the bone marrow revealed a myeloid maturation arrest at the promyelocyte stage. Interestingly, only patient 1 showed severe central nervous system involvement associated with neurocognitive developmental delay and spasticity. Thus, monoallelic mutations in *CLPB* may present with typical features of severe congenital neutropenia. Patients 2 and 3 were refractory to high doses of G-CSF therapy and were therefore treated with hematopoietic stem cell transplantation.

### Identification of critical residues enabling CLPB-HAX1 interaction.

We next aimed at identifying residues that confer interaction between HAX1 and CLPB.

**Figure 2:**
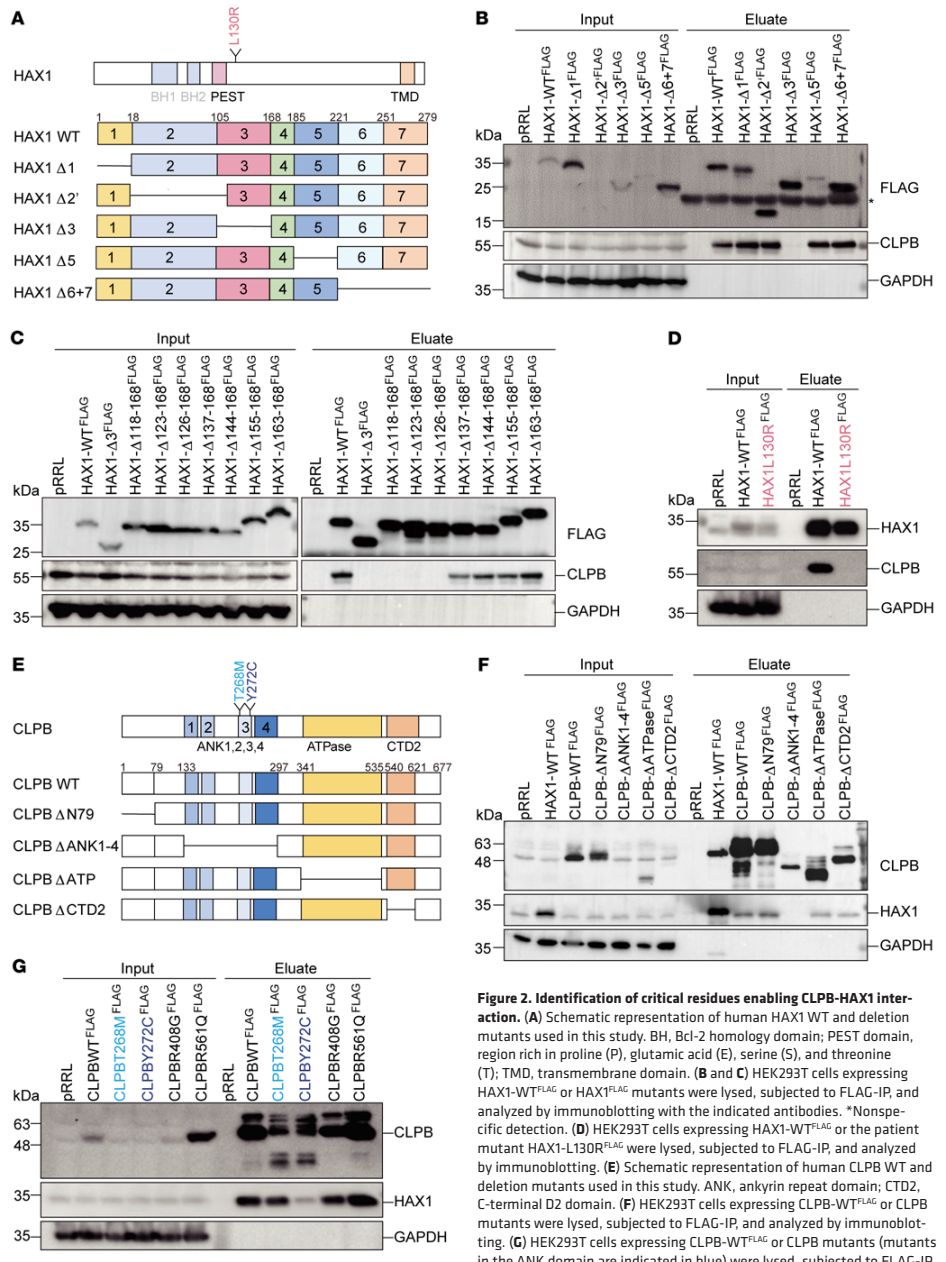
Identification of critical residues enabling CLPB-HAX1 interaction. (**A**) Schematic representation of human HAX1 WT and deletion mutants used in this study. BH, Bcl-2 homology domain; PEST domain, region rich in proline (P), glutamic acid (E), serine (S), and threonine (T); TMD, transmembrane domain. (**B** and **C**) HEK293T cells expressing HAX1-WT^FLAG^ or HAX1^FLAG^ mutants were lysed, subjected to FLAG-IP, and analyzed by immunoblotting with the indicated antibodies. *Nonspecific detection. (**D**) HEK293T cells expressing HAX1-WT^FLAG^ or the patient mutant HAX1-L130R^FLAG^ were lysed, subjected to FLAG-IP, and analyzed by immunoblotting. (**E**) Schematic representation of human CLPB WT and deletion mutants used in this study. ANK, ankyrin repeat domain; CTD2, C-terminal D2 domain. (**F**) HEK293T cells expressing CLPB-WT^FLAG^ or CLPB mutants were lysed, subjected to FLAG-IP, and analyzed by immunoblotting. (**G**) HEK293T cells expressing CLPB-WT^FLAG^ or CLPB mutants (mutants in the ANK domain are indicated in blue) were lysed, subjected to FLAG-IP, and analyzed by immunoblotting.

HAX1 is a 35 kDa protein that contains 2 domains sharing similarity to BCL2 domains, followed by a PEST sequence and C-terminal transmembrane domain (Figure 2A and ref. 39). To gain insights into which protein domains of HAX1 support the binding to CLPB, different FLAG-tagged full-length and truncated versions of HAX1 were expressed by transient transfection in HEK293T cells and isolated by FLAG-IPs. First, we compared association with CLPB between full-length HAX1 and the 5 deletion constructs of HAX1 (summarized in Figure 2A).

Full-length WT and other truncations coprecipitated with CLPB respectively; in contrast, HAX1 lacking exon 3 was unable to interact with CLPB (Figure 2B). To further narrow down the interaction site in exon 3, we generated additional deletion constructs. In particular, a construct lacking residues 126–168 did not associate with CLPB (Figure 2C), while a deletion construct encompassing amino acids 137–168 could still confer interaction. Thus, formation of the CLPB-HAX1 complex is mediated by residues between HAX1 amino acids 126 and 136. Interestingly, in the identified HAX1 binding region resides the known disease-causing variant L130R, which is associated with severe congenital neutropenia and severe neurological symptoms (40).

We generated FLAG-tagged expression vectors carrying WT *HAX1* or the patient-specific mutation *L130R*, transfected them into HEK293T cells, and subjected the encoded WT and mutated proteins to IP. As shown in Figure 2D, only HAX1^WT^-FLAG was able to coprecipitate with CLPB, indicating that L130 of HAX1 is a critical residue enabling the interaction to CLPB.

Reciprocally, we next aimed at mapping the HAX1 binding region in CLPB. CLPB comprises an ankyrin repeat (ANK) domain, which consists of 4 well-conserved subdomains; an ATPase domain (NBD); and a small C-terminal D2 (CTD2) domain (34) (scheme in Figure 2E). By overexpression of CLPB isoform 1 (amino acids 1–707) and isoform 2 (amino acids 1–677) in HEK293T cells and HL-60 cells, respectively, we found that CLPB isoform 2 displayed molecular weight similar to that of endogenous CLPB (Supplemental Figure 2A). Unlike in previous reports, HAX1 interacted with CLPB isoform 2 (amino acids 1–677) rather than isoform 1 (amino acids 1–707) (Supplemental Figure 2B and refs. 21, 34). Meanwhile, reconstitution of CLPB isoform 2 reversed the reduced detection of HAX1 in CLPB^–/–^ cells (Supplemental Figure 2C). Therefore, we chose isoform 2 (amino acids 1–677) for further experiments.

Next, we generated FLAG-tagged full-length and truncated versions of these important functional elements of CLPB and expressed them in HEK293T cells. CLPB^WT^-FLAG and CLPB lacking the first 79 residues efficiently bound to HAX1 (Figure 2F). In contrast, CLPB with either its ATPase domain or its CTD2 domain deleted appeared to associate less efficiently with HAX1. The absence of the ANK domain fully abolished the interaction with HAX1. This suggests that the interaction of CLPB with HAX1 critically depends on its ANK domain.

Defined variants clustered in the ANK domain of CLPB have previously been linked to the rare autosomal recessive mitochondrial disorder 3-methylglutaconic aciduria, type VII (MGCA7), associating neurological impairment and neutropenia (21). While genotype-phenotype relationships in CLPB deficiency remain to be established in detail, some variants affecting the ANK domain, in particular CLPB^Y272C^, are known to be associated with congenital neutropenia. We therefore examined whether known genetic variants may compromise the binding of CLPB to HAX1.

Among the genetic variants studied, only the CLPB^Y272C^ mutant within the ANK domain almost completely lost its ability to bind to HAX1 (Figure 2G).

Taken together, out results indicate that exon 3 in HAX1 and the ANK domain in CLPB confer interaction between these 2 proteins. Specifically, 2 severe-neutropenia-causing mutants in HAX1 and CLPB, L130 and Y272, respectively, emerge as key regulatory interaction sites enabling protein-protein formation.

### A CLPB/HAX1 axis regulates mitochondrial proteostasis.

**Figure 3:**
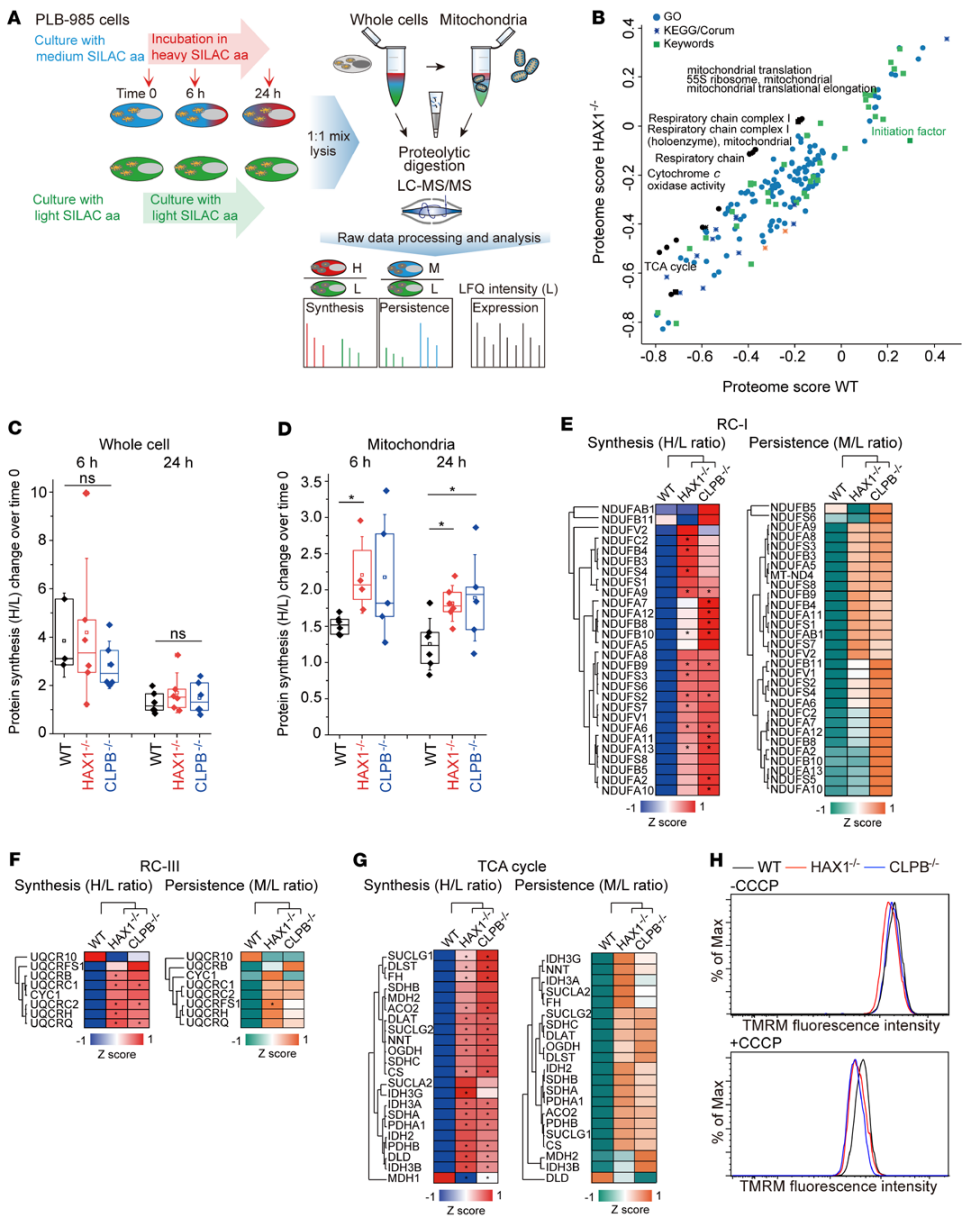
A CLPB/HAX1 axis regulates mitochondrial proteostasis. (**A**) SILAC-based workflow for the analysis of protein dynamics and processing of cells for peptide digestion and liquid chromatography–tandem mass spectrometry analysis. H/L and M/L indicate protein synthesis and persistence. (**B**) 2D annotation enrichment in the whole-cell protein synthesis (H/L) data at 24 hours. The significant annotations with the largest differences are marked in black with names. Annotation type is indicated. The analysis was performed on the median of *n* = 6. Significance was determined using Benjamini-Hochberg FDR with a threshold value of 0.02. (**C** and **D**) Increased mitochondrial protein synthesis in HAX1^–/–^ and CLPB^–/–^ cells. A protein synthesis rate proxy was derived by calculation of the changes in median H/L ratio at 6 hours and 24 hours compared with 0 hour. This was calculated in whole cells (**C**) and mitochondria (keyword annotation) (**D**) (*n =* 6, *P < 0.05, 1-way ANOVA followed by Tukey’s test). (**E**–**G**) Unsupervised hierarchical clustering of protein synthesis (H/L, left) and protein persistence (M/L, right) for mitochondrial RC-I (**E**), RC-III (**F**), and TCA cycle (**G**) (filtered by Gene Ontology annotation), comparing WT, HAX1^–/–^, and CLPB^–/–^ clones after 24 hours of pulse with heavy amino acids. Clusters are based on *z* scores as indicated. Significant differences (*n =* 6, *P < 0.05, Student’s *t* test) are marked with an asterisk. Only proteins with 100% valid SILAC ratios were used for the analysis. (**H**) Measurement of MMP in WT, HAX1^–/–^, and CLPB^–/–^ PLB-985 cells in the absence (top) or presence (bottom) of CCCP (2.5 μM) using TMRM (2.5 nM). Data represent 3 independent experiments.

In view of the CLPB/HAX1 axis, we next hypothesized that HAX1 controls proteostasis in mitochondria of promyeloid cells and made use of an integrated approach combining pulse-chase stable isotope labeling by amino acids in cell culture (SILAC) with mass spectrometry. This strategy allows for the quantitative assessment of protein synthesis and persistence kinetics both in whole cells and in purified mitochondria (workflow scheme in Figure 3A). We decided to use the human promyelocytic leukemic PLB-985 cell line for the SILAC-based proteomics analysis.

PLB-985 cells (WT, HAX1^–/–^, and CLPB^–/–^) were cultured in medium containing arginine and lysine with light isotopes of carbon, hydrogen, and nitrogen (i.e., ^12^C^14^N) (light [L]) or containing l-arginine-^13^C_6_^14^N_4_ and l-lysine-^2^H_4_ (medium [M]) for 10 cell divisions to achieve >99% incorporation of L or M amino acids. Then, M-labeled cells were subjected to medium containing l-arginine-^13^C_6_^15^N_4_ and l-lysine-^13^C_6_^15^N_2_ (“heavy,” H) to initiate pulse labeling for defined time points, and we isolated either whole cells or mitochondria. Thus, ratios of H/L and M/L reflect different protein kinetics as synthesis (assembly) and persistence, respectively (41).

To reveal pathways whose protein synthesis was most affected by HAX1 deficiency, we used 2D annotation enrichment analysis (42). Consistent with the localization of HAX1, mitochondria-related annotation terms such as mitochondrial translation, mitochondrial respiratory chain, and TCA cycle significantly differed in HAX1-deficient cells compared with WT (Figure 3B).

This prompted us to adopt the H/L SILAC ratio as a proxy to compare protein synthesis of WT, HAX1^–/–^, and CLPB^–/–^ cells. We determined the median H/L value of each cell clone at time points 0, 6 hours, and 24 hours and calculated the variation of protein synthesis for each clone (*n =* 6 per genotype). This calculation was applied to data sets derived from both isolated mitochondria and whole cells. While no significant difference in protein synthesis could be observed in whole cells (Figure 3C), the level of protein synthesis was significantly elevated in HAX1- and CLPB-deficient mitochondria compared with WT (Figure 3D).

We then subdivided the proteins of the mitochondrial respiratory chain into the individual complexes and compared the median mitochondrial protein synthesis (H/L ratio) and persistence (M/L ratio) of WT, HAX1-deficient, and CLPB-deficient cells (Figure 3, E and F, and Supplemental Figure 3, A and B). We only focused on proteins whose H/L and M/L ratios were quantified in all clones in 2 replicates (*n =* 6 per genotype).

In comparison with WT, we detected a significantly higher H/L ratio of 11 proteins out of 28 respiratory chain complex I (RC-I) subunits in HAX1-deficient and 12 proteins in CLPB-deficient mitochondria (Figure 3E). The median M/L ratio, our proxy for protein persistence, displayed higher in HAX1- and CLPB-deficient mitochondria as well (Figure 3E). RC-III displayed a very similar behavior to RC-I, with 5 of 8 quantified proteins showing higher H/L ratio in HAX1 deficiency and 3 in CLPB deficiency (Figure 3F). For those whose protein synthesis rate appeared to be increased, their protein persistence also exhibited a slight upregulation (Figure 3F). In the SILAC analysis of both RC-I and RC-III, the differences in H/L ratio between WT and both HAX1- and CLPB-deficient cells shared a similar trend and a largely overlapping statistical significance, suggesting that both proteins are indeed functionally connected. For RC-IV and RC-V, the dynamics of protein synthesis were more variable, especially in HAX1^–/–^ mitochondria, where few proteins displayed significantly higher protein synthesis than in WT (Supplemental Figure 3, A and B). In RC-V we observed only ATP5C1 to be more synthesized in HAX1- and CLPB-deficient cells (Supplemental Figure 3B). The variability of M/L ratio was also evident in RC-IV and RC-V (Supplemental Figure 3, A and B). With respect to protein persistence (M/L ratio), we observed that HAX1- and CLPB-deficient cells tended toward an increase in RC-I, RC-III, and RC-V, yet the level of significance was not reached except for the RC-III member UQCRFS1 in HAX1-KO mitochondria (Figure 3, E and F, and Supplemental Figure 3B).

In addition, we observed that within the TCA cycle, defined by keyword annotations, 21 of 22 proteins showed higher protein synthesis and slightly increased protein persistence in HAX1- and CLPB-deficient cells compared with WT (Figure 3G). Interestingly, MDH, a cytosolic protein of the aspartate/malate shuttle, shows a different dynamic profile in comparison with the other components of the TCA cycle. In contrast to mitochondrial pathways, variable trends of protein synthesis/persistence were observed in non-mitochondrial pathways such as Golgi apparatus or the pathway of glycolysis (Supplemental Figure 3, C and D, and Supplemental Tables 2 and 3).

Given the higher protein synthesis rate among mitochondrial proteins, we next studied the expression of the key mitochondrial biogenesis regulators PGC1α and TFAM (43).

We examined the expression of PGC1α and TFAM by immunoblotting in control and in HAX1- and CLPB-deficient cells (Supplemental Figure 3E). In addition, TFAM expression was documented by quantitative proteomics. The endogenous levels of PGC1α were not altered among control, HAX1-deficient, and CLPB-deficient cells. The endogenous expression of TFAM was slightly increased in HAX1- and CLPB-deficient cells compared with control cells (Supplemental Figure 3E). The label-free quantification (LFQ) intensities of TFAM were significantly higher in HAX1-deficient cells, while they were only slightly higher in CLPB-deficient cells than in WT cells (Supplemental Figure 3F).

We next examined whether the altered synthesis rates impact on mitochondrial function. We measured the mitochondrial membrane potential (MMP) as an indicator of mitochondrial activity in control, HAX1-deficient, and CLPB-deficient PLB-985 cells, respectively. FACS analyses revealed a slightly lower fluorescence intensity of tetramethylrhodamine methyl ester (TMRM) in HAX1- and CLPB-deficient cells (Figure 3H, top). In the presence of the mitochondrial uncoupler carbonyl cyanide *m*-chlorophenyl hydrazone (CCCP), HAX1- and CLPB-deficient PLB-985 cells showed increased dissipation of the MMP in comparison with control cells (Figure 3H, bottom).

### HAX1 is required for respiratory chain complex activity.

**Figure 4:**
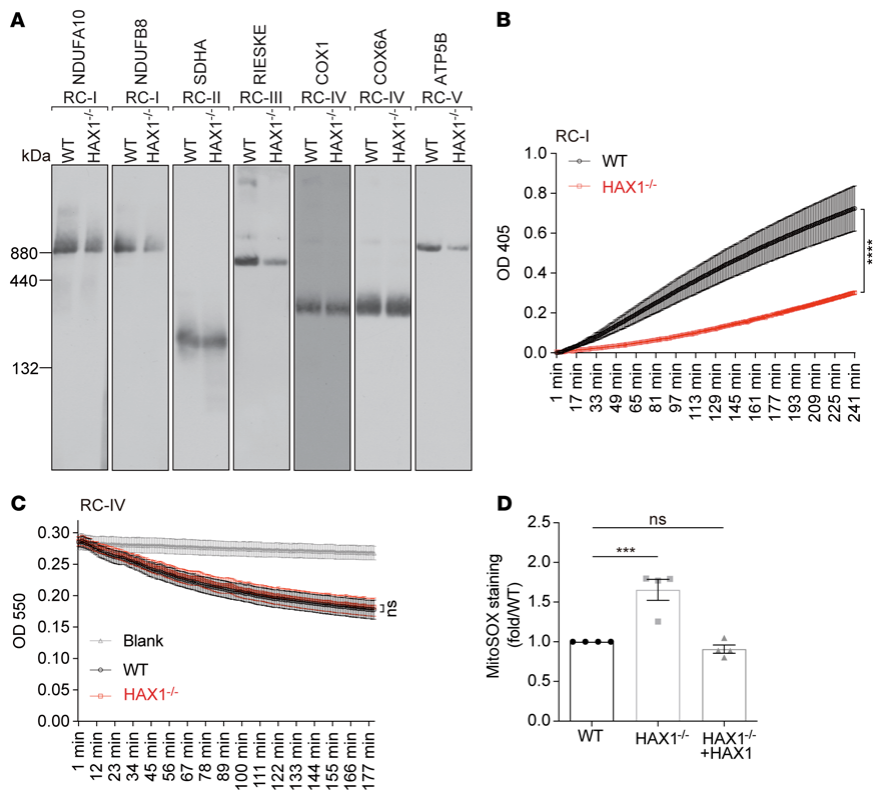
HAX1 is required for respiratory complex activity. (**A**) Mitochondria from WT and HAX1^–/–^ cells were isolated and analyzed by blue native PAGE. The mild detergent digitonin was used for solubilization. The amount of RCs I–V was quantified using the indicated signals. (**B** and **C**) Mitochondria derived from WT or HAX1^–/–^ PLB-985 cells were subjected to quantification of RC-I activity (**B**) (*n =* 3, ****P < 0.0001, 2-way ANOVA followed by Bonferroni’s test) or RC-IV activity (**C**) (*n =* 3, 2-way ANOVA followed by Bonferroni’s test). (**D**) Quantification of mitochondrial ROS production in WT, HAX1^–/–^, or HAX1^–/–^ reconstituted with HAX1 cells using Mitochondrial Superoxide Indicator (MitoSOX) (*n =* 4, ***P < 0.001, 1-way ANOVA followed by Tukey’s test).

To corroborate our finding of altered RC dynamics in HAX1 deficiency, we next analyzed the steady-state amount of RCs I–V by blue native PAGE (BN-PAGE) in WT and HAX1-deficient cells (Figure 4A). Loss of HAX1 led to reduced levels of RC-I, RC-III, and RC-V (Figure 4A), indicating that HAX1 regulates the steady-state amount of these RCs.

Next, we were interested to examine whether an imbalance in protein synthesis and turnover in RCs affects the physiological function of these complexes. We therefore assessed the enzymatic activities of RC-I in both WT and HAX1^–/–^ cells, using an assay that relies on immune capture of RC-I from freshly isolated mitochondria. The NADH dehydrogenase activity of immune-captured RC-I was determined by the oxidation of NADH and simultaneous reduction of the provided dye resulting in increase in absorbance. Mitochondria isolated from HAX1^–/–^ cells exhibited significantly reduced RC-I activity compared with mitochondria purified from WT cells (Figure 4B), while the activity of RC-IV was not affected in HAX1-KO cells (Figure 4C).

To test the functional relevance of altered RC-I/RC-III protein dynamics, we next asked whether ROS production is affected in HAX1-deficient cells. Mitochondrial O_2_^–^ levels were significantly increased in the absence of HAX1 compared with WT, as revealed by Mitochondrial Superoxide Indicator (MitoSOX; Thermo Fisher Scientific) (Figure 4D). HAX1-KO cells that were functionally complemented with HAX1 restored efficient O_2_^–^ levels (Figure 4D). Thus, our data identify HAX1 as a critical regulator of mitochondrial respiratory chain complex activity.

### HAX1 regulates HSP27 in mitochondria.

**Figure 5:**
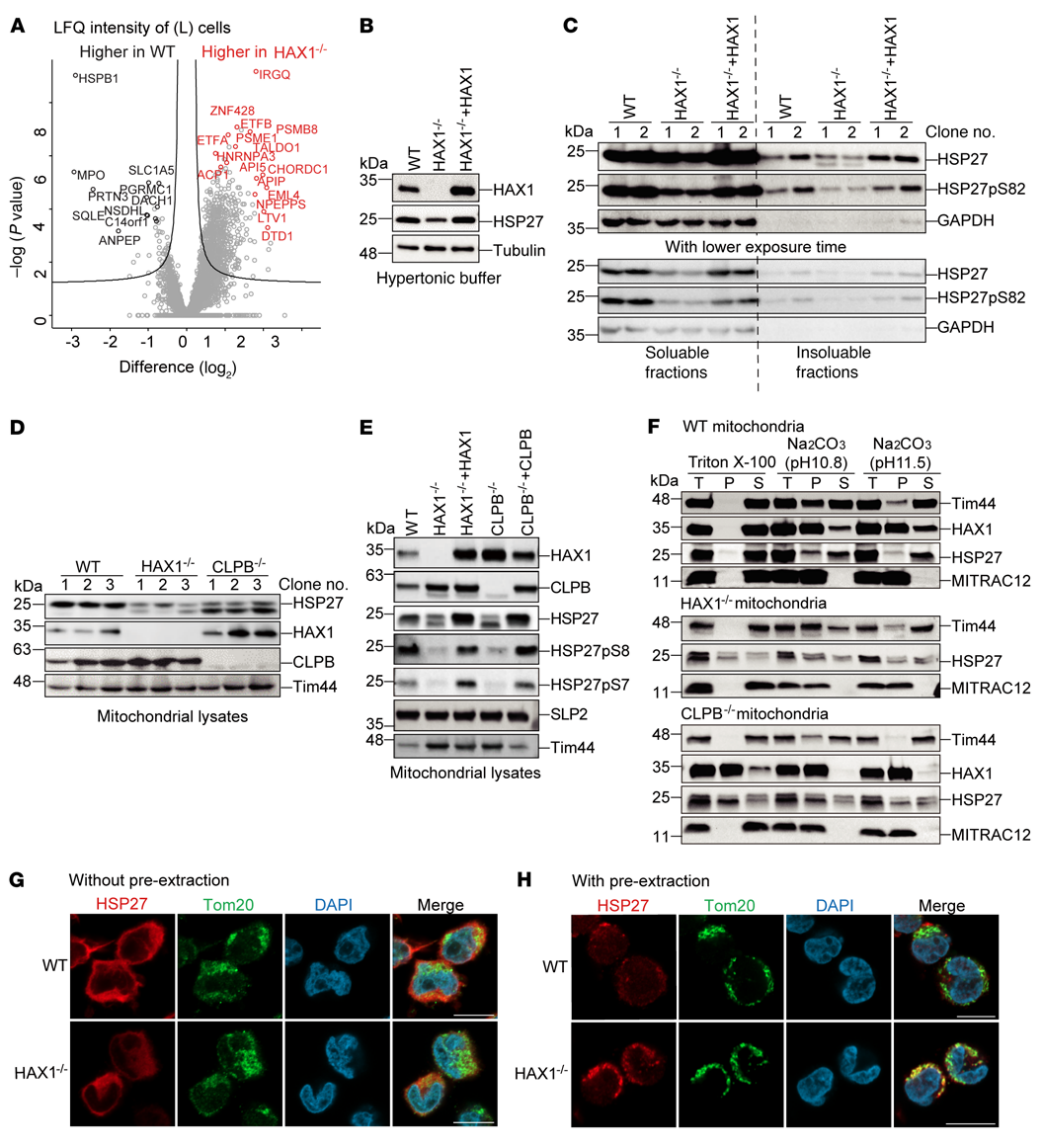
HAX1 regulates phosphorylation and solubility of HSP27 in mitochondria. (**A**) Volcano plot of changes in protein expression (LFQ intensity, SILAC light) between WT and HAX1^–/–^ PLB-985 cells (*n =* 18 each, 2 replicates). Data were filtered for at least 3 valid values in total. The comparison highlights 71 proteins with significantly higher expression in WT and 159 proteins in HAX1^–/–^ clones. Proteins marked in black (higher in WT) and red (higher in HAX1^–/–^) highlight a subset of proteins with absolute fold change greater than 2 and *P* value less than 0.001. (**B**) PLB-985 cells harvested from the indicated genotypes were subjected to hypertonic lysis and analyzed by immunoblotting with indicated antibodies. This immunoblot is additionally shown in Supplemental Figure 4A, experiment 1, top panel. (**C**) PLB-985 cells from the indicated genotypes were subjected to hypertonic lysis. After centrifugation, supernatants were collected as soluble fractions, and pellets were resolved in Laemmli buffer as insoluble fractions. All samples were analyzed with higher or lower exposure time by immunoblotting. (**D** and **E**) Isolated mitochondria from the indicated genotypes were solubilized with Laemmli buffer and analyzed by immunoblotting. (**F**) Purified WT, HAX1^–/–^, or CLPB^–/–^ mitochondria were subjected to carbonate extraction or lysed with Triton X-100 and separated into supernatant (S) and pellet (P) fractions. All lysates were analyzed by immunoblotting. T, total. (**G** and **H**) Immunostaining of HSP27 and TOM20 in PLB-985 WT or HAX1^–/–^ cells without (**G**) or with (**H**) pre-extraction treatment. DNA was visualized with DAPI. Scale bars: 10 μm.

In an effort to define critical downstream mediators of HAX1, we compared the proteome of WT and HAX1-deficient PLB-985 cells for differential protein expression using mass spectrometry and LFQ. We included a total of 4372 proteins with at least 2 peptides used for LFQ quantification and more than 3 valid values in total. The volcano plot of differentially expressed proteins is shown in Figure 5A. One of the most downregulated proteins in HAX1-deficient cells was HSP27, a molecular chaperone that maintains cellular proteostasis by preventing the aggregation of partially unfolded proteins (44–46). In its unphosphorylated form, HSP27 assembles into large insoluble oligomeric complexes, whereas phosphorylation results in complex dissociation into smaller soluble oligomeric ensembles (47).

To examine whether HAX1 is implicated in the regulation of these 2 oligomeric assembly states of HSP27, we prepared cell lysates from WT and HAX1-deficient cells as well as from HAX1-reconstituted cells under hypertonic buffer conditions (a condition in which only small soluble HSP27 oligomers can be efficiently extracted) and performed immunoblotting to assess the abundance of HSP27. Under hypertonic lysis conditions, less HSP27 could be extracted from HAX1-deficient cells in comparison with control and HAX1-reconstituted cells (Figure 5B and Supplemental Figure 4A, top panel). In addition, we prepared whole-cell lysates using SDS-PAGE sample buffer, enabling us to efficiently extract and to solubilize large oligomeric HSP27. These immunoblot analyses revealed that, under these conditions, almost identical amounts of HSP27 could be extracted in WT and HAX1-deficient cells as well as from HAX1-reconstituted cells (Supplemental Figure 4A, bottom panel). These results indicate that HAX1 is involved in the regulation of the dynamic oligomeric assembly states of HSP27.

As the level of HSP27 phosphorylation correlates with the solubility of HSP27, soluble and insoluble fractions of cell extracts were recovered and examined for the total amount of HSP27 and phosphorylated HSP27 (p-HSP27) by immunoblot analysis. Intriguingly, in the absence of HAX1, HSP27 appeared in 2 forms that migrated with different rates in the insoluble fraction (Figure 5C). To test whether these 2 forms of HSP27 correlate with the phosphorylation status of HSP27 and hence with the solubility of HSP27, we used phospho-specific antibodies raised against serine 82 (HSP27pS82) (Supplemental Figure 4B). Indeed, the upper band corresponded to p-HSP27 (Figure 5C), while the lower band specifically present in HAX1^–/–^ cells could not be detected by the HSP27pS82 specific antibody. This suggests that HAX1 is involved in maintaining the phosphorylated, soluble form of HSP27. Intrigued by the observation that HAX1, a mitochondrial IMS protein, may control phosphorylation of HSP27, a protein primarily localized in the cytosol, we were interested in whether distinct variants of HSP27 may be detectable in mitochondria and performed immunoblotting of HSP27 derived from mitochondrial lysates of WT, HAX1^–/–^, and CLPB^–/–^ cell clones. Not only in the absence of HAX1, but also in the absence of CLPB, we could document the appearance of the lower HSP27-specific band, corresponding to the unphosphorylated, insoluble form (Figure 5D). In the absence of HAX1 and CLPB, both signals specific to pS82 and pS78 were largely reduced. Upon retrovirus-mediated reconstitution of HAX1 and CLPB, the signal of HSP27pS82 and HSP27pS78 could be detected at a level comparable to that in control cells (Figure 5E). These findings indicate that the CLPB-HAX1 complex specifically regulates the phosphorylation state of HSP27 in mitochondria.

To provide evidence that HAX1 regulates the solubility of HSP27, we performed experiments involving carbonate extraction of isolated mitochondria from WT, HAX1^–/–^, and CLPB^–/–^ PLB-985 cells and examined HSP27 by immunoblotting. Under non-ionic lysis conditions, HSP27, similarly to the membrane-bound protein TIM44, was recovered from the supernatant (S) and was released from the mitochondrial membrane into the supernatant (S) at pH 11.5 in WT mitochondria (Figure 5F). In contrast, in the absence of HAX1 or CLPB, HSP27 partially remained in the pellet fraction under different lysis and extraction conditions (Figure 5F). In line with our previous data, the unphosphorylated, insoluble form of HSP27 was enriched in the pellet fractions indicated by the appearance of the second HSP27-specific band (Figure 5F). To confirm that HAX1 controls solubility of HSP27, we next performed confocal microscopy studies in PLB-985 cells. In native conditions, the majority of HSP27 was found throughout the cytoplasm, while only a small proportion located to the mitochondria, as shown by costaining of the translocase TOM20, a mitochondrial outer membrane protein (Figure 5G). By contrast, when soluble proteins were removed before fixation, the HSP27 protein largely disappeared in WT cells, whereas in HAX1^–/–^ cells HSP27 was detectable, in particular in mitochondria, evidenced by a clustered punctate pattern and partial colocalization with TOM20 (Figure 5H).

Taken together, our data demonstrate that HAX1 ensures the efficient phosphorylation and solubility of HSP27 in mitochondria.

### PRKD2 is a mitochondrial kinase involved in HSP27 phosphorylation.

**Figure 6:**
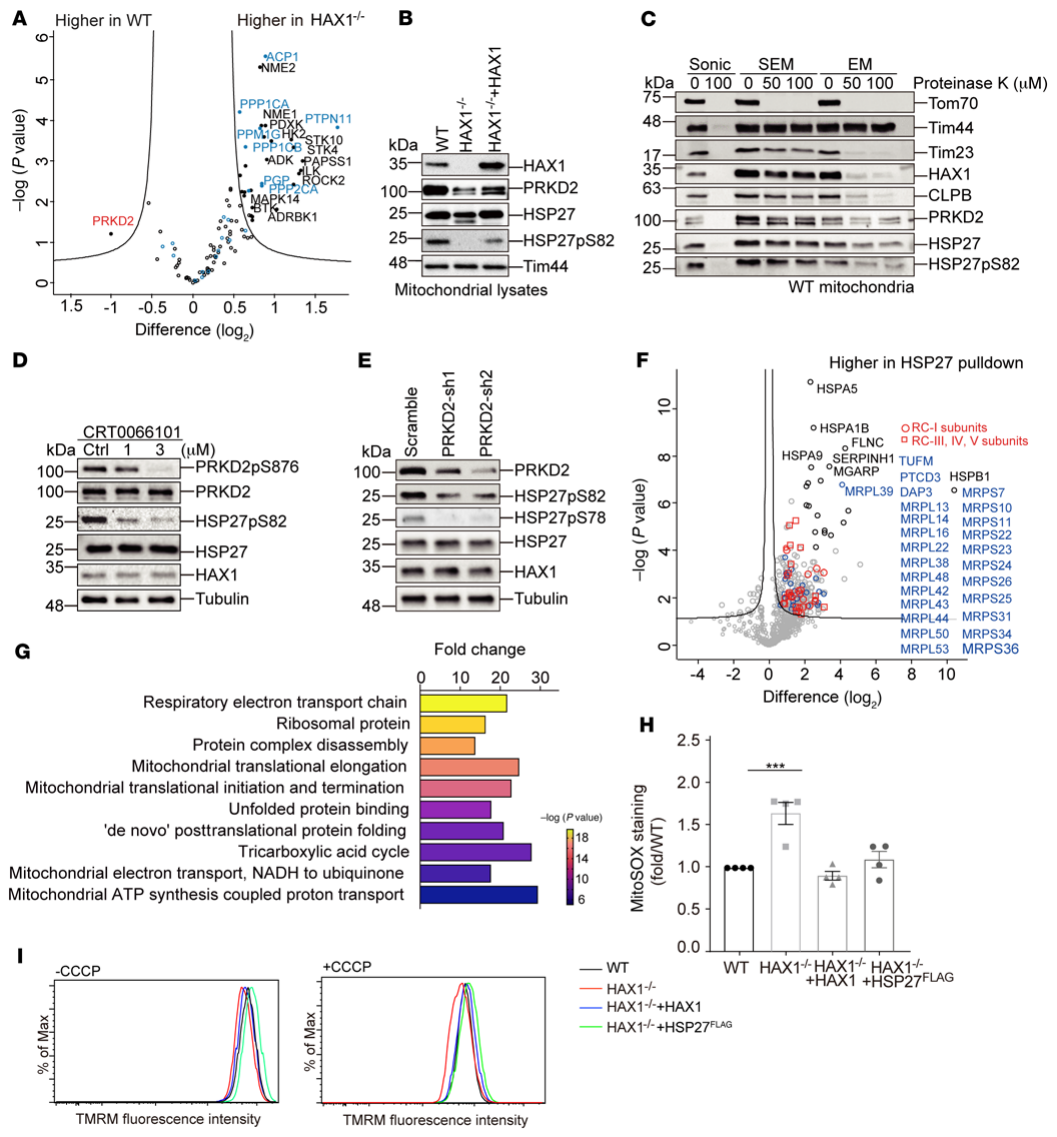
PRKD2 is a mitochondrial kinase involved in HSP27 phosphorylation. (**A**) Volcano plot comparing protein expression (LFQ intensity, SILAC light) of kinases (black) and phosphatases (blue, both keyword annotation) in WT and in HAX1^–/–^ clones (*n =* 18 each, 2 replicates). Data were filtered for 14 valid values in at least 1 group (106 proteins in total). (**B**) Isolated mitochondria from indicated genotypes were solubilized with Laemmli buffer and analyzed by immunoblotting. (**C**) Isolated mitochondria were swollen or sonicated and/or treated with PK and analyzed by immunoblotting (EM: EDTA, MOPS; SEM: sucrose, EDTA, MOPS). (**D**) Lysates of PLB-985 cells treated with the protein kinase D inhibitor CRT0066101 were analyzed by immunoblotting with the indicated antibodies. (**E**) Lysates of PLB-985 cells expressing doxycycline-inducible shRNA targeting either control or PRKD2 were analyzed by immunoblotting with the indicated antibodies. (**F**) Volcano plot illustrating the mitochondrial interactome of HSP27 (*n =* 6) versus control (non-bait) (*n =* 6). The analysis is based on 724 proteins that were commonly identified in 2 biological replicates. The bait (HSP27/HSPB1) and the interactors with the highest *P* values are marked in black. Significant interactors annotated as mitochondrial translation (Gene Ontology) are marked in red and blue and listed. (**G**) Gene Ontology Biological Process pathway enrichment analysis of the HSP27 interactome (**F**, right), color-coded by enrichment *P* value as indicated. (**H**) Quantification of mitochondrial ROS production in WT, HAX1^–/–^, and HAX1^–/–^ cells reconstituted with either HAX1 or HSP27^FLAG^ cells using MitoSOX (*n =* 4, ***P < 0.001, 1-way ANOVA followed by Tukey’s test). (**I**) MMP in WT, HAX1^–/–^, HAX1^–/–^ + HAX1, or HAX1^–/–^ + HSP27 PLB-985 cells in the absence or presence of CCCP (2.5 μM) by TMRM (2.5 nM). Data represent 3 independent experiments.

Several protein kinase families have been implicated in targeting HSP27 for phosphorylation (48). To date, however, phosphorylation of HSP27 in mitochondria and the respective kinases have not been studied in detail. In search of candidate kinases, we queried our protein expression data set (LFQ intensity, light SILAC labeling, filtered for 25% valid values in total) for “kinase” and “protein phosphatase” (keyword annotation). Among 106 candidate proteins retrieved and shown in the volcano plot, only serine-threonine protein kinase D2 (PRKD2) had significantly higher expression in WT compared with HAX1^–/–^ cells (Figure 6A).

PRKD2, a member of the protein kinase D (PKD) family, has previously been implicated in leukemogenesis (49, 50), but its molecular targets in hematopoietic cells remain largely unclear. To validate our mass spectrometry result, we assessed protein expression of PRKD2 in mitochondria isolated from WT and HAX1^–/–^ cells as well as HAX1-reconstituted PLB-985 cells. In the absence of HAX1, PRKD2 expression was reduced (Figure 6B and Supplemental Figure 5, A–E). Since PRKD2 has not yet been reported to be spatially confined to mitochondria, we next studied whether PRKD2 and HSP27 are both located inside mitochondria. Following our experimental strategy outlined in Figure 1, treatment of mitochondria with PK resulted in the degradation of Tom70 (Figure 6C, lane 1), whereas PRKD2, HSP27, Tim23, HAX1, and CLPB remained unaffected after PK treatment (Figure 6C, lanes 3–6). Interestingly, p82-HSP27 behaved similarly to PRKD2 and remained intact (Figure 6C, lane 8). Upon osmotic disruption of the outer mitochondrial membrane, a portion of PRKD2, HSP27, and p-HSP27 was resistant to protease treatment, indicating that they are localized in the mitochondrial IMS and in the matrix. To directly test whether PRKD2 supports phosphorylation of HSP27, we inhibited PRKD2 using CRT0066101, a PKD inhibitor (51), and determined the phosphorylation status of HSP27 by using the phospho-specific antibody pS82. Notably, there was a dose-dependent decrease in pS82 in PLB-985 cells (Figure 6D and Supplemental Figure 5, F–J). In a more specific and complementary approach, we used doxycycline-inducible shRNA targeting *PRKD2* transcript. In comparison with control PLB-985 cells, the partial depletion of PRKD2 resulted in reduced phosphorylation of HSP27 at both S82 and S78 (Figure 6E and Supplemental Figure 5, K–N).

In view of the critical function of HSP27 for HAX1-dependent mitochondrial proteostasis, we were interested in defining downstream targets of HSP27 by IP studies. Mitochondria lysates from HSP27-FLAG–expressing cells as well control cells were subjected to FLAG-IP and mass spectrometry analysis. In contrast to control IPs, mitochondria from HSP27-FLAG–expressing cells were enriched for proteins that are part of the respiratory electron transport chain, mitochondrial translational pathways, “de novo” protein folding machineries, and TCA cycle (Figure 6, F and G). Notably, the group of highly enriched proteins included key RNA-binding proteins, such as MRPSs, MRPLs, and TUFM, indicative of an involvement of HSP27 in translation (52, 53).

We assume that in the absence of HAX1, decreased phosphorylation of HSP27 will result in decreased solubility and function of HSP27 in mitochondria. This, in turn, may predispose nascent mitochondrial peptides to an imbalanced protein turnover. Consistent with this idea, we found evidence of an increased activation of the Sirt3/FOXO3A/LC3 pathway in HAX1-deficient cells (Supplemental Figure 5O). In the presence of increasing concentrations of the proteasome inhibitor MG132, LC3B abundance was further increased in HAX1-deficient cells, indicating more proneness to mitochondrial proteotoxic stress in HAX1^–/–^ cells in comparison with WT cells.

Interestingly, some subunits of RCs characterized by an increased protein synthesis and persistence were also enriched in the HSP27 IP (e.g., NDUFS2, NDUFS3, UQCRC1, UQCRC2, ATP5C1) (Figure 6F and Supplemental Table 4). We used a MitoSOX-based flow cytometric assay to detect mitochondrial ROS production in control and in HAX1-deficient cells reconstituted with either HAX1 or HSP27-FLAG (Supplemental Figure 5P). HAX1-deficient cells reconstituted with either HAX1 or HSP27-FLAG could recover O_2_^–^ levels to a degree similar to that of cells expressing HAX1, as revealed by the superoxide indicator MitoSOX (Figure 6H).

In addition, we analyzed MMP as an indicator of mitochondrial function in control cells and HAX1-deficient cells reconstituted with either HAX1 or HSP27. FACS analyses revealed that a lower fluorescence intensity of TMRM in the presence of CCCP in HAX1-deficient cells could be restored by reconstitution with HAX1 or HSP27 (Figure 6I, bottom).

### HSP27 restores defective neutrophil differentiation in HAX1-deficient induced pluripotent stem cells.

**Figure 7:**
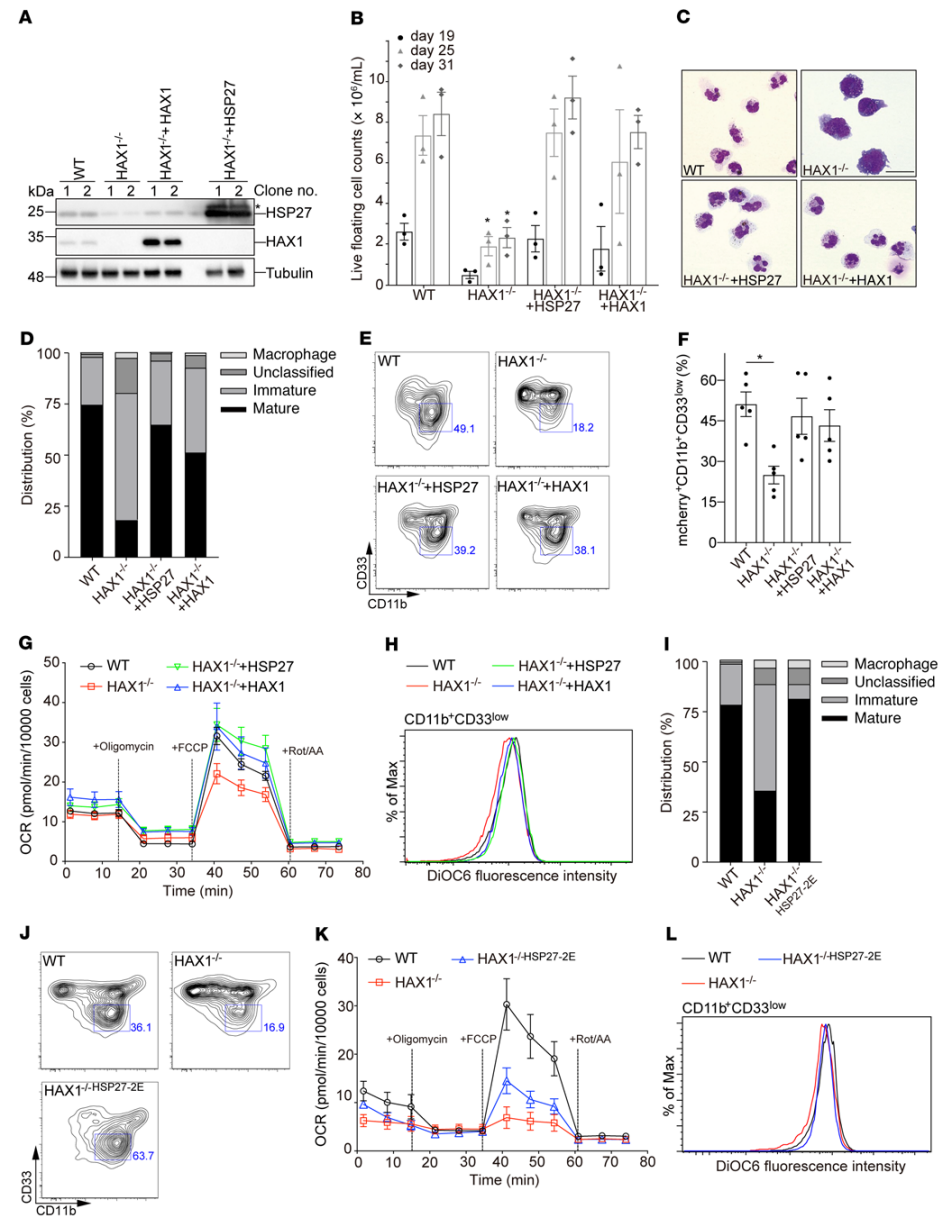
HSP27 overexpression restores neutrophil differentiation defect in HAX1^–/–^ iPSCs. (**A**) Immunoblot analysis of WT and HAX1-deficient iPSCs overexpressing either HSP27 or HAX1. *An additional band detected by the HSP27 antibody after HSP27 overexpression in iPSCs. (**B**) Live floating cells generated from indicated iPSC colonies (6 per well) were quantified during differentiation (*n =* 3, *P < 0.05, 2-way ANOVA followed by Tukey’s test). (**C**) Light microscopy of iPSC-derived immature and mature neutrophil granulocytes (at day 28) stained with May-Grünwald Giemsa. Scale bar: 10 μm. (**D**) Quantification of the distribution of immature (myeloblasts, promyelocytes, myelocytes, and metamyelocytes) and mature neutrophil granulocytes (band and segmented neutrophils). (**E** and **F**) Analysis of differentiated neutrophil-like cells on day 28 by FACS (**E**) and quantification of immature iPSC-derived neutrophil granulocytes (mCherry^+^CD11b^+^CD33^lo^) (**F**) (*n =* 5, *P < 0.05, 1-way ANOVA followed by Tukey’s test). (**G**) Measurement of oxygen consumption rate (OCR) in hematopoietic progenitor cells derived from the indicated genotypes (at day 18 of iPSC-derived neutrophil granulocyte differentiation) following a sequential addition of oligomycin, FCCP, and rotenone (Rot) and antimycin A (AA). (**H**) Measurement of MMP in CD11b^+^CD33^lo^ populations derived from WT, HAX1^–/–^, HAX1^–/–^ + HAX1, or HAX1^–/–^ + HSP27 iPSCs using DiOC6. (**I**) Quantification of the distribution of precursor populations in iPSC-derived myeloid cells from the indicated genotypes. Data represent 2 independent experiments. (**J**) Analysis of differentiated neutrophil-like cells (CD11b^+^CD33^lo^) at day 28 of differentiation by FACS. Data represent 3 independent experiments. (**K**) Measurement of OCR in hematopoietic progenitor cells derived from the indicated genotypes (at day 18 of iPSC-derived neutrophil granulocyte differentiation) following a sequential addition of oligomycin, FCCP, and Rot and AA. (**L**) Measurement of MMP in CD11b^+^CD33^lo^ populations derived from WT, HAX1^–/–^, or HAX1^–/–HSP27-2E^ iPSCs using DiOC6 at day 28 of differentiation. Data represent 3 independent experiments.

Since RC-I dysfunction is not the causative mechanism for neutropenia in HAX1-deficient patients, we sought to query a more relevant model system. We made use of an induced pluripotent stem cell (iPSC) in vitro differentiation system (Supplemental Figure 6A), allowing us to engineer human neutrophils by CRISPR/Cas9–mediated genome editing. We generated iPSCs deficient in expression of HAX1 and reconstituted these cells with either HAX1 or HSP27 by the inducible expression transposon system *piggyBac* (Figure 7A and ref. 54).

During the differentiation process, the number of floating cells, representing myeloid cells at various stages of development, increased between day 19 and day 25 in control iPSCs, whereas HAX1-deficient iPSCs had a significantly reduced capacity to differentiate into neutrophil granulocytes (Figure 7B). Importantly, the number of progenitor cells could be reestablished by the expression of HAX1 or HSP27 (Figure 7B). HAX1-deficient cells displayed not only quantitative but also qualitative defects. Light microscopy of Giemsa-stained cells showed mostly immature myeloid cells with dark cytoplasm and nonsegmented nuclei in HAX1-deficient iPSCs (Figure 7C). In contrast to control cells, HAX1-deficient cells had a skewed distribution of mature versus immature neutrophil granulocytes (Figure 7D). In striking contrast, both HAX1 and HSP27 expression restored differentiation toward mature segmented neutrophils (Figure 7, C and D). Similarly, the correction of the skewing of HAX1-deficient cell differentiation could also be determined by flow cytometry. Whereas HAX1-deficient cells did not mature into a CD11b^+^CD33^lo^ population, control cells as well as reconstituted cells showed coordinated expression of these CD markers (Figure 7, E and F).

We next examined mitochondrial function using Agilent Seahorse in hematopoietic progenitor cells (at day 18 of iPSC-derived neutrophil granulocyte differentiation). Mitochondrial ATP production and maximal respiratory capacity, as measured after addition of the uncoupler carbonyl cyanide-4-trifluoromethoxyphenylhydrazone (FCCP), were significantly lower in HAX1-deficient iPSC-derived cells compared with control iPSC-derived cells (Figure 7G and Supplemental Figure 6, B–D). Importantly, ATP production and respiratory capacity were restored upon reconstitution of HSP27 or HAX1 (Figure 7G and Supplemental Figure 6, B–D).

In addition, we examined the membrane potential in mature iPSC-derived neutrophil granulocytes (at day 28 of iPSC-derived neutrophil granulocyte differentiation). We used 3,3′-dihexyloxacarbocyanine iodide (DiOC6) as a probe to study MMP in CD11b^+^CD33^lo^ expressing cells by flow cytometry. These FACS analyses revealed that the fluorescence intensity of DiOC6 in HAX1-deficient iPSC-derived neutrophil granulocytes was reduced compared with that in control iPSC-derived neutrophil granulocytes and iPSC-derived neutrophil granulocytes reconstituted with HAX1 or HSP27 (Figure 7H), confirming an impaired mitochondrial function in mature HAX1-deficient iPSC-derived neutrophil granulocytes.

Taken together, our results demonstrate that HSP27 functionally complements HAX1 during the coordinated differentiation of neutrophil granulocytes.

We then examined the functional consequences of phosphomimic mutations of HSP27 in HAX1-deficient iPSC-derived cells. As the phosphorylation of both S82 and S78 is reduced in HAX1-deficient PLB-985 cells (Figure 5E), we introduced the phosphomimic residues S82E and S78E of HSP27 by CRISPR/Cas9–mediated gene editing in HAX1-deficient iPSCs, referred to as HAX1^–/–HSP27-2E^, and analyzed the effect on neutrophil development. The introduction of these phosphomimic residues rendered the HSP27 epitope inaccessible to the phospho-specific antibodies pS82 and pS78, thus validating our HAX1^–/–HSP27-2E^ cell line (Supplemental Figure 6E).

During the differentiation process, the number of myeloid progenitor cells increased in control iPSCs, whereas HAX1-deficient iPSCs and HAX1-deficient cells with the phosphomimic residues in HSP27 (HAX1^–/–HSP27-2E^) had a significantly lower number of progenitor and mature granulocytes (Supplemental Figure 6F). Low floating cell numbers in HAX1-deficient iPSC-derived neutrophil granulocytes with phosphomimic mutations in HSP27 might arise from aberrant colony formation during neutrophil differentiation (Supplemental Figure 6G).

Light microscopy of Giemsa-stained control, HAX1-deficient, and HAX1^–/–HSP27-2E^ iPSC-derived neutrophil granulocytes revealed that differentiation toward mature neutrophils with segmented and band nuclei was, however, restored in HAX1^–/–HSP27-2E^ iPSCs (Figure 7I and Supplemental Figure 6H). Similarly, the phosphomimic mutations of HSP27 in HAX1-deficient iPSC-derived neutrophil granulocytes (HAX1^–/–HSP27-2E^) expressed CD surface markers, CD11b^+^CD33^lo^, resembling those in mature neutrophil granulocytes (Figure 7J). The phosphomimic mutations of HSP27 in HAX1-deficient iPSC-derived myeloid progenitors partially restored the maximal respiratory capacity in Seahorse experiments (Figure 7K). Our flow cytometry analyses revealed comparable MMP values of control and HAX1^–/–HSP27-2E^ iPSC-derived neutrophil granulocytes (Figure 7L).

Thus, these results corroborate our previous finding that HSP27 phosphorylation is critical for neutrophil granulocyte differentiation and function.

## Discussion

We here identify a functional CLPB/HAX1/(PRKD2)/HSP27 axis controlling mitochondrial proteostatic networks critical for the differentiation of human neutrophil granulocytes.

We identify CLPB (isoform 2) as a critical interaction partner of HAX1 in human cells. CLPB is a member of the AAA+ (ATPases associated with diverse cellular activities) family of proteins that use energy derived from ATP hydrolysis to unfold bound substrates or remodel and dissociate protein complexes (31). AAA+ proteins assemble into asymmetric hexameric rings that hydrolyze ATP and thread substrate proteins through a central channel via mobile substrate-binding “pore loops” (33, 55). Conserved Tyr residues within these pore loops coordinate substrate binding with their efficient translocation. The human nucleotide binding domain (NBD) NBD2 of CLPB closely resembles the NBD2 of Hsp104 and Hsp78, while the N-terminal NBD1 and middle domain are replaced by an ANK domain (56). We here describe Y272 within the ANK domain as a key residue enabling CLPB-HAX1 complex formation. Interestingly, a subset of patients with mutations in *CLPB* is characterized not only by progressive brain dysfunction but also by congenital neutropenia (57). With the identification of L130 in HAX1 we thus verify 2 severe-neutropenia-causing mutants in HAX1 and CLPB, L130 and Y272, respectively, as essential residues for complex formation/stability and provide mechanistic evidence explaining a genotype-phenotype correlation. The critical dependence of HAX1 on CLPB is furthermore underscored by the fact that both CLPB-deficient myeloid cells and HAX1-deficient myeloid cells share similar imbalances of their proteome composition, such as defects in HSP27 and dysfunction of respiratory complex proteins.

Surprisingly, we also identify monoallelic *CLPB* variants (*G560R*, *R561Q*, and *R620H*) associated with severe congenital neutropenia. This is in line with very recent work by the Link and Wortmann laboratories (58, 59). The identified variants reside within the C-terminal ATP-binding domain. While the R561Q mutant is competent for HAX1 binding (Figure 2G), the mutant R561G (in which at the same position the arginine is substituted for glutamine) was shown to be impaired in its ATPase and disaggregase activity (59). Thus, R561 is a critical residue enabling proper CLPB function.

Human CLPB has recently been shown to function as a potent “stand-alone” mitochondrial disaggregase (34). The inner-membrane protease PARL — a known interaction partner of HAX1 — removes an autoinhibitory peptide from CLPB to enhance disaggregase activity (34). Since murine Hax1 has been shown to present HtrA2/Omi to the mitochondrial protease Parl (17), it is conceivable that HAX1 plays a role in regulating CLPB processing as well. PARL mediates cleavage of a variety of mitochondrial membrane proteins, linking it closely to mitochondrial homeostasis and diseases with mitochondrial dysfunction (60).

Interestingly, the consequences of HAX1 and CLPB deficiency for the composition of the mitochondrial proteome are highly similar, supporting the functional relevance of the CLPB-HAX1 interaction. In myeloid cells lacking either HAX1 or CLPB, dynamics of synthesis and persistence of proteins composing respiratory complexes (RC-I and RC-III) as well as the TCA cycle are severely perturbed. Accumulation of proteins assembling into RC-I led to reduced enzymatic activity. Since RC-I contains more than twice as many subunits in comparison with the other RCs, its association may be more complex and vulnerable.

Interestingly, the respiratory chain complexes (RC-I, RC-III) that were significantly affected in their assembly dynamics by the loss of HAX1 and CLPB originate from both mitochondrial and nuclear genes. Thus, we assume that proper RC-I and RC-III assembly is subject to tight translational control of both mitochondrial and cytosolic ribosomes. Presumably, coupling and synchronization of mitochondrial translation with import of cytosolic proteins require a high degree of coordination. In accordance with this idea, a translational plasticity pathway enabling adaptation of mitochondrial protein synthesis to the influx of nuclear-encoded subunits has been identified (61). It is therefore conceivable that RC-I and RC-III assembly strictly depends on a fine-tuned balance of mitochondrial and cytosolic translational activities.

Recently, the yeast homolog of CLPB, HSP104, was described as an essential key player in the “mitochondria as guardian in cytosol” (MAGIC) pathway (62). It will be interesting to examine whether CLPB acts as an essential guardian in human cells that couples mitochondrial to cellular proteostasis.

Chaperones such as HSP70 and CLPB homologs are known to cooperate with cochaperones assisting in protein quality control. We identified HSP27 as one of the most differentially expressed proteins in HAX1-deficient cells. HSP27 belongs to the family of ATP-independent chaperones that are well known for their roles in protein maturation, refolding, and degradation. HSPs are thought to maintain their client proteins in a soluble, folding-competent state for subsequent processing by ATP-dependent chaperones. Phosphorylation of the N-terminal domain of HSP27 combined with competitive interactions for the β4 to β8 grooves induces HSP27 oligomer disassembly and subsequently leads to the active chaperone-competent state of HSP27 (63, 64). Here, we find that in the absence of CLPB or HAX1, the unphosphorylated form of HSP27 accumulated concomitant with its insolubility in mitochondrial lysates.

As key elements of the cellular protein quality control system, small HSPs such as HSP27 are known to play pivotal roles in cell survival (65). Their function during cell development and differentiation is less well understood. HSP27 expression was shown to transiently increase during early phases of the differentiation process concomitantly with the gradual growth arrest that precedes terminal differentiation. Here, we find that the inducible and transient overexpression of HSP27 in HAX1-deficient iPSCs is capable of restoring the ordered differentiation and mitochondrial function of iPSC-derived progenitor and mature neutrophil granulocytes. The observation that phosphomimicking mutants (S78E and S82E) of HSP27 in HAX1-deficient iPSCs failed to restore the number of progenitor and mature neutrophil granulocytes might suggest that dynamic cycles between phosphorylated and unphosphorylated states are critical to serve HSP27 chaperone function.

Reversible phosphorylation events have been reported to play a critical role during HSP27-mediated rescue of neuronal plasticity deficits (64). The knockin of the phosphomimicking mutants (S78E and S82E) of HSP27 recovers the differentiation of fewer neutrophil granulocytes albeit displaying segmented nuclei expressing mature CD surface markers in HAX1-deficient iPSCs. This might indicate that tight spatial and temporal control of the phosphorylation status of HSP27 could be a critical regulatory mechanism to safeguard HSP27 function during neutrophil differentiation.

Based on our data, PRKD2, a member of the calcium/calmodulin-dependent protein kinase superfamily (66), emerges as a mitochondrial kinase involved in the phosphorylation of HSP27. A possible explanation for the reduced expression of PRKD2 in HAX1-deficient cells could be transcriptional regulation of *PRKD2* by a retrograde signaling pathway induced by mitochondrial stress. We observed a reduction in *PRKD2* mRNA level in HAX1-deficient cells by quantitative PCR (Supplemental Figure 5Q). This transcriptional regulation of *PRKD2* by HAX1 could be indirect or only partially dependent on HAX1, as the reconstitution of HAX1 in HAX1-deficient cells could only partially restore PRKD2 expression.

Several lines of evidence suggest that neutrophil differentiation in the bone marrow is under distinct metabolic control by mitochondrial respiration, TCA cycle, and oxidative phosphorylation (67). Mechanistic insights into the role of mitochondria for this metabolic adaptation are still unclear.

Recent studies have revealed that loss of the mitochondrial complex III subunit Rieske iron-sulfur protein (RISP) leads to metabolite alterations (increased 2-hydroxyglutarate) that alter DNA and histone methylation and subsequently impair hematopoietic differentiation (8). Notably, in our SILAC data of HAX1-deficient cells, Uqcrfs1 encoding for RISP was significantly altered in its persistence.

In light of our data, we speculate that the neutrophil differentiation defects in HAX1 deficiency may in part be due to an imbalance of critical enzymes required for proper mitochondrial function.

Our discovery of a functional CLPB/HAX1/(PRKD2)/HSP27–dependent axis involved in the maintenance of mitochondrial proteostasis and its role in the differentiation of neutrophil granulocytes highlights an emerging theme in the development and function of the innate immune system.

## Methods

### Cell culture.

HeLa and HEK293T cells were cultured in DMEM (Gibco). PLB-985 cells were cultured in RPMI 1640 (Gibco), supplemented with 10% (vol/vol) FBS (Life Technologies), 50 U/mL penicillin, 50 μg/mL streptomycin, 2 mM l-Glu, and 10 mM HEPES (all from Gibco). Healthy control fibroblast-derived iPSCs were provided by Micha Drukker from Helmholtz Center Munich in Neuherberg, Germany. Detailed description of iPSC culture and generation of neutrophil granulocytes is provided in Supplemental Methods. Cells were grown under a 5% CO_2_ humidified atmosphere at 37°C. Cells were tested weekly for mycoplasma. All cell lines used in this study are described in Supplemental Table 8.

### sgRNA design and cloning for targeted gene correction in cell lines.

sgRNAs targeting *HAX1* and *CLPB* were designed with the online CRISPR Design tool developed by Feng Zhang’s laboratory (http://crispr.mit.edu/). sgRNA-Cas9 with fusion *GFP* or *RFP* plasmids was purchased from Addgene (PX458). sgRNAs were inserted into the plasmid following the protocol of Feng Zhang and colleagues (68). All sgRNAs used in this study are listed in Supplemental Table 5. Molecular cloning strategies and transfection experiments in this study are described in detail in Supplemental Methods.

### Antibodies.

All primary and secondary antibodies as well as fluorescent secondary antibodies used in this study are listed in Supplemental Table 6. Chemicals, peptides, and recombinant proteins used in this study are listed in Supplemental Table 7.

### Mitochondrial swelling and carbonate experiment.

Protocols for mitochondrial swelling and carbonate were modified according to a previous report (69). (For the swelling, isolated mitochondria were incubated with proteinase K after SEM buffer, EM buffer, or sonification treatments. For carbonate extraction, isolated mitochondria were solubilized with 0.1 M Na_2_CO_3_ at pH 10.8 or pH 11.5. Details regarding mitochondrial isolation, swelling, and carbonate extraction are given in Supplemental Methods.

### Immunofluorescence studies.

Fixed cells were quenched in 50 mM NH_4_Cl for 30 minutes before permeabilization with 0.2% Triton for 2 minutes at room temperature prior to blocking. Coverslips were then incubated with indicated primary and secondary antibodies before DAPI staining. Cell pre-extraction is described in Supplemental Methods.

### Measurement of mitochondrial MMP.

MMP was measured using TMRM (2.5 nM; MilliporeSigma) or DiOC6 (0.5 nM; MilliporeSigma) indicators according to the manufacturer instructions. In brief, cells were incubated with MMP indicators at 37°C for 15 minutes prior to a washing step and FACS (BD Biosciences).

### Seahorse assay.

A XF96 extracellular flux analyzer (Agilent Technologies) was applied to determine the bioenergetic profile of intact cells. Hematopoietic stem cells were harvested, seeded onto poly-d-lysine–coated XF96 plates (7 × 10^4^ cells per well), and incubated in RPMI medium (Agilent Technologies; supplemented with 5 mM glucose, 2 mM l-glutamine, and 1% FBS) for 1 hour in a CO_2_-free incubator. Oxygen consumption rate (OCR) was first measured in basal conditions (named as basal respiration) before cells were sequentially treated with 1.5 μM oligomycin (MilliporeSigma), 1 μM FCCP (MilliporeSigma), and 0.5 μM rotenone/antimycin A (MilliporeSigma) and 8 μM Hoechst (Thermo Fisher Scientific). Recorded OCR after all treatments was defined as non-mitochondrial respiration, which was subtracted from all OCR parameters. ATP production was calculated by the difference between the basal and oligomycin-inhibited respiration; maximal respiratory capacity was obtained as the rate of respiration after uncoupler FCCP treatment. Hoechst-positive cells were counted by a Cytation machine (Agilent). The number of live cells was normalized to individual sample for a final evaluation of sample OCR by Wave software (version 2.6.3, Agilent).

### SILAC labeling.

PLB-985 cells were cultured in SILAC RPMI medium (Gibco) lacking arginine and lysine and containing 10% (vol/vol) dialyzed FBS. This medium was supplemented with light standard unlabeled lysine and arginine, medium isotope-labeled ^13^C_4_-lysine and ^13^C_6_-arginine, or heavy isotope-labeled ^13^C_6_^15^N_2_-lysine and ^13^C_6_^15^N_4_-arginine (Silantes). Medium-labeled cells were pulsed with heavy medium for the indicated numbers of hours. Pulsed cells were mixed with the same number of “light” labeled cells (41). More details regarding SILAC analysis are given in Supplemental Methods.

### Data availability.

The mass spectrometry proteomics data were deposited in ProteomeXchange (ProteomeXchange Consortium) via the Proteomics Identifications Database (PRIDE) partner repository with the data set identifier PXD023790. Detailed relevant materials and methods are described in the supplemental material.

### Statistics.

In SILAC analysis, annotation enrichments were derived by Fisher’s exact test, using Benjamini-Hochberg FDR for truncation and a threshold value of 0.02. To determine the lists of proteins significantly changing among WT, HAX1-deficient, and CLPB-deficient clones, we performed Student’s 2-tailed *t* test using 0.05 FDR for truncation and 250 randomizations. Hierarchical clustering was performed on SILAC values (log_2_) after *z* scoring. All data are represented as mean ± SEM. Statistically significant differences (*P <* 0.05) were calculated with the help of Prism software (GraphPad Software) by unpaired, 2-tailed Student’s *t* test to compare 2 groups or by 1-way or 2-way ANOVA or multiple *t* test with Holm-Šidák to compare multiple groups as indicated in the figure legends.

### Study approval.

Patients were referred to the academic centers of the coauthors, who sent blood samples for genetic workup to LMU for further investigations. Informed consent/assent for the genetic and immunological studies, as well as their publication, was obtained from all legal representatives and patients. Genetic and functional studies on biosamples from patients and their relatives were performed under the framework of a scientific project entitled “Genetic characterization of congenital bone marrow failure and immunodeficiency syndromes.” This study was approved in 2011 by the ethics committee at LMU (346-11, 381-11) and includes permission to publish the results.

## Author contributions

CK, YF, M Murgia, and MIL conceptualized the study. YF, M Murgia, YM, and SD provided methodology. YF, SD, and YM provided validation. YF, M Murgia, YM, MŁ, and NZ provided formal analysis. YF provided visualization. M Murgia provided computational analysis. YF, M Murgia, YM, SD, CW, and YL provided investigation. SF, GS, APG, ZA, and NR obtained and analyzed clinical data. CK, M Mann, and PR provided resources. CK, MIL, M Mann, and PR supervised the study. MIL, YF, and CK wrote the original drafts of the manuscript. YF, MIL, YM, and SF revised the manuscript. All authors wrote, reviewed, and edited the manuscript. All authors read and approved the manuscript before submission.

## Supplementary Material

Supplemental data

## Figures and Tables

**Table 1:**
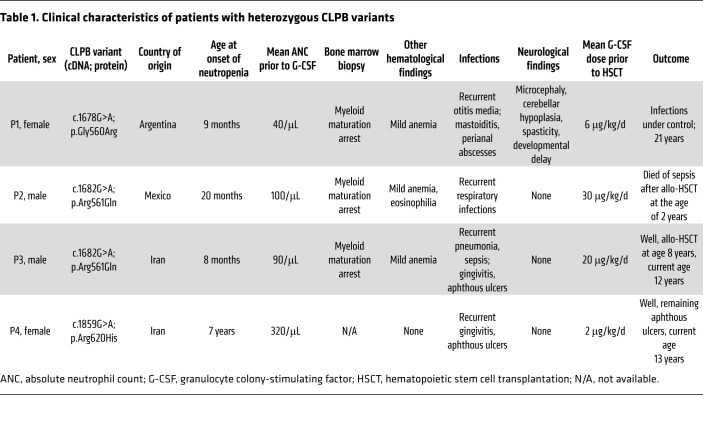
Clinical characteristics of patients with heterozygous CLPB variants

## Version 1 05/02/2022

Electronic publication

## References

[1] Mantovani A (2011). Neutrophils in the activation and regulation of innate and adaptive immunity. Nat Rev Immunol.

[2] Cowland JB, Borregaard N (2016). Granulopoiesis and granules of human neutrophils. Immunol Rev.

[3] Cassatella MA (2019). Biological roles of neutrophil-derived granule proteins and cytokines. Trends Immunol.

[4] Soehnlein O (2009). Neutrophil granule proteins tune monocytic cell function. Trends Immunol.

[5] Tecchio C (2014). Neutrophil-derived cytokines: facts beyond expression. Front Immunol.

[6] Ward AC (2000). Regulation of granulopoiesis by transcription factors and cytokine signals. Leukemia.

[7] Riffelmacher T (2017). Autophagy-dependent generation of free fatty acids is critical for normal neutrophil differentiation. Immunity.

[8] Anso E (2017). The mitochondrial respiratory chain is essential for haematopoietic stem cell function. Nat Cell Biol.

[9] Baker MJ (2011). Quality control of mitochondrial proteostasis. Cold Spring Harb Perspect Biol.

[10] Spinelli JB, Haigis MC (2018). The multifaceted contributions of mitochondria to cellular metabolism. Nat Cell Biol.

[11] Song J (2021). Quality control of the mitochondrial proteome. Nat Rev Mol Cell Biol.

[12] Dudek J (2013). Mitochondrial protein import: common principles and physiological networks. Biochim Biophys Acta.

[13] Wiedemann N, Pfanner N (2017). Mitochondrial machineries for protein import and assembly. Annu Rev Biochem.

[14] Grevel A (2019). Coupling of import and assembly pathways in mitochondrial protein biogenesis. Biol Chem.

[15] Deshwal S (2020). Mitochondrial proteases: multifaceted regulators of mitochondrial plasticity. Annu Rev Biochem.

[16] Jadiya P, Tomar D (2020). Mitochondrial protein quality control mechanisms. Genes (Basel).

[17] Chao JR (2008). Hax1-mediated processing of HtrA2 by Parl allows survival of lymphocytes and neurons. Nature.

[18] Klein C (2007). HAX1 deficiency causes autosomal recessive severe congenital neutropenia (Kostmann disease). Nat Genet.

[19] Chen X (2019). Targeting mitochondrial structure sensitizes acute myeloid leukemia to venetoclax treatment. Cancer Discov.

[20] Saunders C (2015). CLPB variants associated with autosomal-recessive mitochondrial disorder with cataract, neutropenia, epilepsy, and methylglutaconic aciduria. Am J Hum Genet.

[21] Wortmann SB (2015). CLPB mutations cause 3-methylglutaconic aciduria, progressive brain atrophy, intellectual disability, congenital neutropenia, cataracts, movement disorder. Am J Hum Genet.

[22] Owusu-Ansah E, Banerjee U (2009). Reactive oxygen species prime Drosophila haematopoietic progenitors for differentiation. Nature.

[23] Tormos KV (2011). Mitochondrial complex III ROS regulate adipocyte differentiation. Cell Metab.

[24] Zhang J (2011). UCP2 regulates energy metabolism and differentiation potential of human pluripotent stem cells. EMBO J.

[25] Hamanaka RB (2013). Mitochondrial reactive oxygen species promote epidermal differentiation and hair follicle development. Sci Signal.

[26] Antonicka H (2020). A high-density human mitochondrial proximity interaction network. Cell Metab.

[27] Suzuki Y (1997). HAX-1, a novel intracellular protein, localized on mitochondria, directly associates with HS1, a substrate of Src family tyrosine kinases. J Immunol.

[28] Dennerlein S (2015). MITRAC7 acts as a COX1-specific chaperone and reveals a checkpoint during cytochrome c oxidase assembly. Cell Rep.

[29] Rassow J (1994). Mitochondrial protein import: biochemical and genetic evidence for interaction of matrix hsp70 and the inner membrane protein MIM44. J Cell Biol.

[30] Klein C (2017). Kostmann’s disease and HCLS1-associated protein X-1 (HAX1). J Clin Immunol.

[31] Neuwald AF (1999). AAA+: a class of chaperone-like ATPases associated with the assembly, operation, and disassembly of protein complexes. Genome Res.

[32] Uchihashi T (2018). Dynamic structural states of ClpB involved in its disaggregation function. Nat Commun.

[33] Rizo AN (2019). Structural basis for substrate gripping and translocation by the ClpB AAA+ disaggregase. Nat Commun.

[34] Cupo RR, Shorter J (2020). Skd3 (human ClpB) is a potent mitochondrial protein disaggregase that is inactivated by 3-methylglutaconic aciduria-linked mutations. Elife.

[35] Squires CL (1991). ClpB is the Escherichia coli heat shock protein F84.1. J Bacteriol.

[36] Thomas JG, Baneyx F (1998). Roles of the Escherichia coli small heat shock proteins IbpA and IbpB in thermal stress management: comparison with ClpA, ClpB, and HtpG In vivo. J Bacteriol.

[37] Weibezahn J (2004). Thermotolerance requires refolding of aggregated proteins by substrate translocation through the central pore of ClpB. Cell.

[38] Wai T (2016). The membrane scaffold SLP2 anchors a proteolytic hub in mitochondria containing PARL and the i-AAA protease YME1L. EMBO Rep.

[39] Fadeel B, Grzybowska E (2009). HAX-1: a multifunctional protein with emerging roles in human disease. Biochim Biophys Acta.

[40] Lanciotti M (2010). Novel HAX1 gene mutations associated to neurodevelopment abnormalities in two Italian patients with severe congenital neutropenia. Haematologica.

[41] Boisvert FM (2012). A quantitative spatial proteomics analysis of proteome turnover in human cells. Mol Cell Proteomics.

[42] Cox J, Mann M (2012). 1D and 2D annotation enrichment: a statistical method integrating quantitative proteomics with complementary high-throughput data. BMC Bioinformatics.

[43] Dillon LM (2012). The role of PGC-1 coactivators in aging skeletal muscle and heart. IUBMB Life.

[44] Arrigo AP (2017). Mammalian HspB1 (Hsp27) is a molecular sensor linked to the physiology and environment of the cell. Cell Stress Chaperones.

[45] Jakob U (1993). Small heat shock proteins are molecular chaperones. J Biol Chem.

[46] Haslbeck M, Vierling E (2015). A first line of stress defense: small heat shock proteins and their function in protein homeostasis. J Mol Biol.

[47] Arrigo AP (2005). In search of the molecular mechanism by which small stress proteins counteract apoptosis during cellular differentiation. J Cell Biochem.

[48] Kostenko S, Moens U (2009). Heat shock protein 27 phosphorylation: kinases, phosphatases, functions and pathology. Cell Mol Life Sci.

[49] Di Bernardo MC (2008). A genome-wide association study identifies six susceptibility loci for chronic lymphocytic leukemia. Nat Genet.

[50] Yang ZF (2013). GABP transcription factor is required for development of chronic myelogenous leukemia via its control of PRKD2. Proc Natl Acad Sci U S A.

[51] Harikumar KB (2010). A novel small-molecule inhibitor of protein kinase D blocks pancreatic cancer growth in vitro and in vivo. Mol Cancer Ther.

[52] Cuesta R (2000). Chaperone hsp27 inhibits translation during heat shock by binding eIF4G and facilitating dissociation of cap-initiation complexes. Genes Dev.

[53] Carper SW (1997). Heat shock protein 27 stimulates recovery of RNA and protein synthesis following a heat shock. J Cell Biochem.

[54] Kim SI (2016). Inducible transgene expression in human iPS cells using versatile all-in-one piggyBac transposons. Methods Mol Biol.

[55] Deville C (2019). Two-step activation mechanism of the ClpB disaggregase for sequential substrate threading by the main ATPase motor. Cell Rep.

[56] Erives AJ, Fassler JS (2015). Metabolic and chaperone gene loss marks the origin of animals: evidence for Hsp104 and Hsp78 chaperones sharing mitochondrial enzymes as clients. PLoS One.

[57] Pronicka E (2017). A scoring system predicting the clinical course of CLPB defect based on the foetal and neonatal presentation of 31 patients. J Inherit Metab Dis.

[58] Warren JT (2022). Heterozygous variants of CLPB are a cause of severe congenital neutropenia. Blood.

[59] Wortmann SB (2021). Neutropenia and intellectual disability are hallmarks of biallelic and de novo CLPB deficiency. Genet Med.

[60] Spinazzi M, De Strooper B (2016). PARL: the mitochondrial rhomboid protease. Semin Cell Dev Biol.

[61] Richter-Dennerlein R (2016). Mitochondrial protein synthesis adapts to influx of nuclear-encoded protein. Cell.

[62] Ruan L (2017). Cytosolic proteostasis through importing of misfolded proteins into mitochondria. Nature.

[63] Freilich R (2018). Competing protein-protein interactions regulate binding of Hsp27 to its client protein tau. Nat Commun.

[64] Abisambra JF (2010). Phosphorylation dynamics regulate Hsp27-mediated rescue of neuronal plasticity deficits in tau transgenic mice. J Neurosci.

[65] Mogk A (2019). Cellular functions and mechanisms of action of small heat shock proteins. Annu Rev Microbiol.

[66] Rozengurt E (2005). Protein kinase D signaling. J Biol Chem.

[67] Kumar S, Dikshit M (2019). Metabolic insight of neutrophils in health and disease. Front Immunol.

[68] Cong L (2013). Multiplex genome engineering using CRISPR/Cas systems. Science.

[69] Mick DU (2012). MITRAC links mitochondrial protein translocation to respiratory-chain assembly and translational regulation. Cell.

