# Supplementary material for: HAX1-dependent control of mitochondrial proteostasis governs neutrophil granulocyte differentiation
Source: J Clin Invest. 2022 May 2;132(9):e153153. doi: 10.1172/JCI153153 (PMC9057593; doi:10.1172/JCI153153)

Supplemental material for

HAX1-dependent control of mitochondrial proteostasis governs neutrophil granulocyte differentiation

Yanxin Fan[1], Marta Murgia[2,3], Monika I. Linder[1], Yoko Mizoguchi[1§], Cong Wang [4†], Marcin Łyszkiewicz[1#], Natalia Ziętara[1¶], Yanshan Liu[1‡], Stephanie Frenz[1], Gabriela Sciuccati[5], Armando Partida-Gaytan[6&], Zahra Alizadeh[7], Nima Rezaei[8], Peter Rehling[4,9,10], Sven Dennerlein[4], Matthias Mann[2], Christoph Klein[1,11]*

[1]Department of Pediatrics, Dr. von Hauner Children's Hospital and Gene Center, University Hospital, LMU, Munich, Germany. [2]Department of Proteomics and Signal Transduction, Max-Planck-Institute of Biochemistry, Martinsried, Germany. [3]Department of Biomedical Sciences, University of Padova, Padua, Italy. [4]Department of Cellular Biochemistry, University Medical Center Goettingen, Goettingen, Germany. [5]Hematology and Oncology Department, Hospital de Pediatria “Prof. Dr. J.P. Garrahan”, Buenos Aires, Argentina. [6]Unidad de Investigación en Inmunodeficiencias Primarias, Instituto Nacional de Pediatría, Mexico City, Mexico. [7]Immunology Asthma and Allergy Research Institute, Tehran university of Medical Science, Teheran, Iran. [8]Research Center for Immunodeficiencies, Children’s Medical Center, Tehran University of Medical Sciences, Teheran, Iran. [9]Cluster of Excellence "Multiscale Bioimaging: from Molecular Machines to Networks of Excitable Cells" (MBExC), University of Goettingen, Goettingen, Germany. [10]Max Planck Institute for Biophysical Chemistry, Goettingen, Germany. [11]Lead contact.

§Present affiliation: Graduate School of Biomedical and Health Sciences (Medical), Hiroshima University, Hiroshima, Japan. †Present affiliation: Leibniz-Forschungsinstitut for Molecular Pharmacology (FMP), Berlin, Germany. #Present affiliation: Department of Pediatrics and Adolescent Medicine, University Medical Center Ulm, Ulm, Germany. ¶Present affiliation: Cancer Immunology and Immune Modulation, Boehringer Ingelheim Pharma GmbH & Co. KG, Biberach an der Riss, Germany. ‡Present affiliation: Laboratory of Genomic and Precision Medicine, Wuxi School of Medicine, Jiangnan University, Wuxi, China. &Present affiliation: Fundación Mexicana para Niñas y Niños con Inmunodeficiencias Primarias A.C., Mexico City, Mexico.

*Corresponding author:

Christoph Klein

Dr. von Hauner Children's Hospital, Lindwurmstr. 4, 80337 Munich, Germany

Tel.: +49 (0) 89 – 4400 57701; Fax: +49 (0) 89 – 4400 57702

Email: christoph.klein@med.uni-muenchen.de

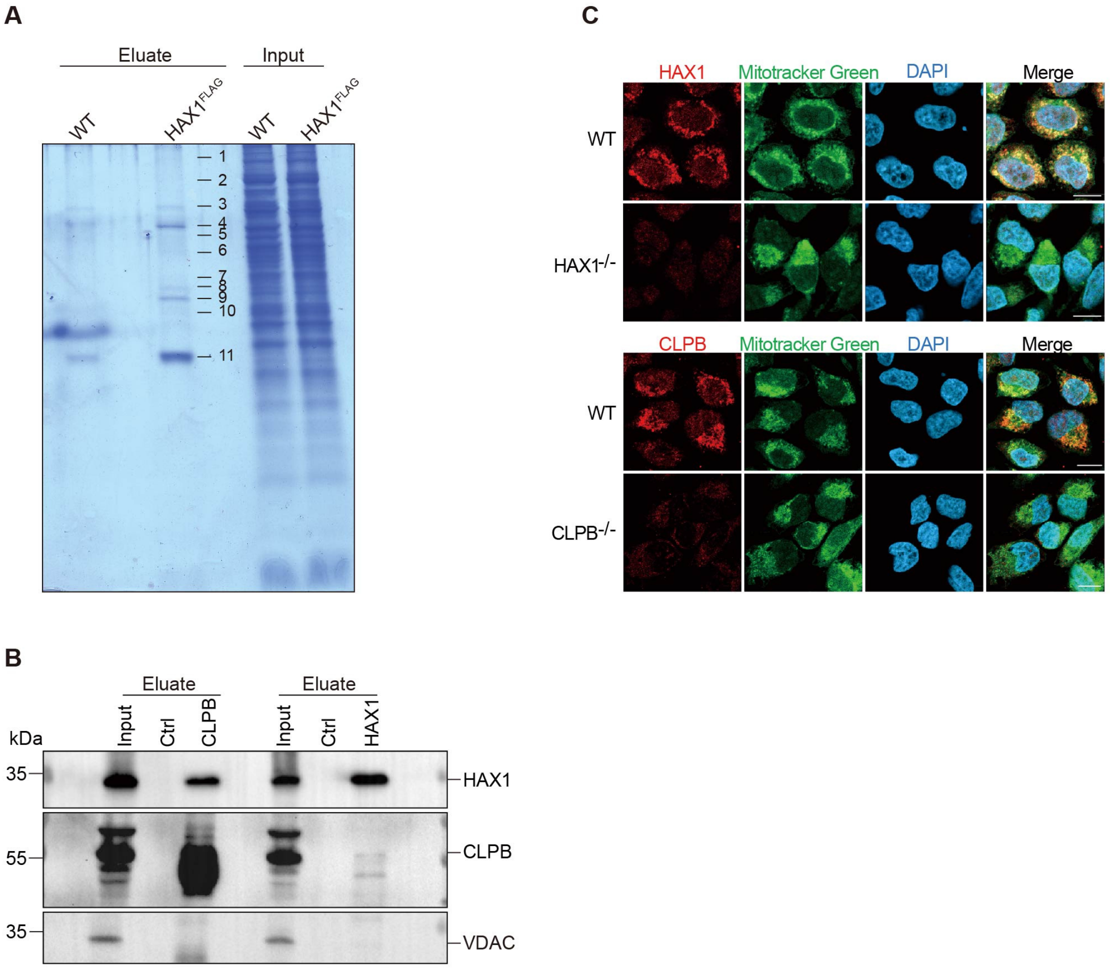

Supplemental Figure 1. Identification of HAX1 interacting proteins. Related to Figure 1. (A) Analysis of the HAX1-FLAG immunoprecipitation experiment by Coomassie-blue staining. Numbered bands were cut and processed for mass spectrometry analysis. The co-purified proteins in the immunoprecipitation of HAX1-FLAG are listed in Supplemental Table 1. (B) Endogenous immunoprecipitation experiment with HAX1 and CLPB antibodies. Input and eluates of precipitated proteins were analyzed by immunoblotting with the indicated antibodies. (C) Confocal images of immunostaining of HAX1 and CLPB in combination with the Mitotracker dye (green) and DAPI (DNA staining) in WT, HAX1$^{-/-}$ and CLPB$^{-/-}$ HeLa cells. Scale bar, 10 µm.

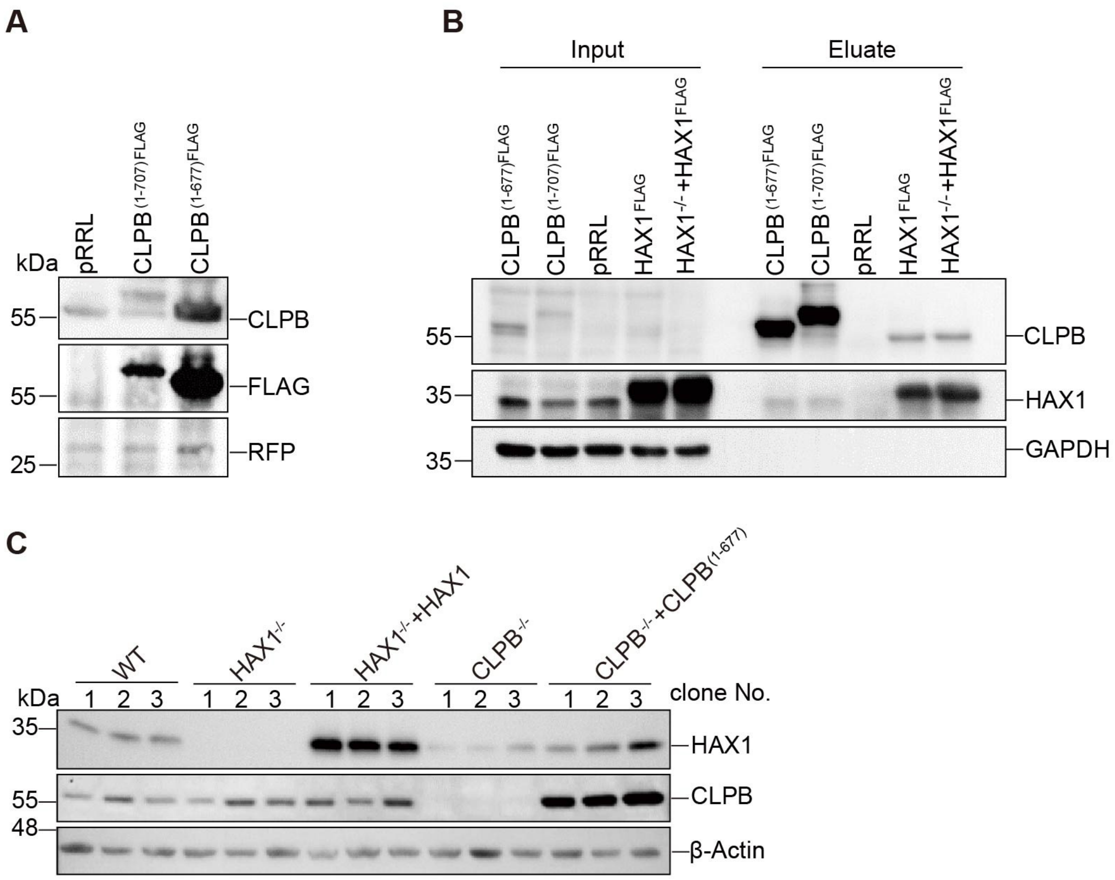

Supplemental Figure 2. Identification of the predominant isoform of CLPB *in vivo*. Related to Figure 2.

(A) HEK293T cells expressing empty pRRL vector, CLPB^{FLAG} isoform 1 (1-707aa) or CLPB^{FLAG} isoform 2 (1-677aa) were lysed and analyzed by immunoblotting. (B) To ensure the isoform of CLPB interacts with HAX1, HL60 cells expressing pRRL vector, CLPB^{FLAG} isoform 1 (1-707aa), CLPB^{FLAG} isoform 2 (1-677aa), HAX1^{FLAG} or HAX1$^{-/-}$ HL60 cells reconstituted with HAX1^{FLAG} were lysed, subjected to FLAG-immunoprecipitation and analyzed by immunoblotting. (C) WT, HAX1$^{-/-}$, HAX1$^{-/-}$+HAX1, CLPB$^{-/-}$ and CLPB$^{-/-}$+CLPB$^{(1-677)}$. PLB-985 cells were lysed and analyzed by immunoblotting.

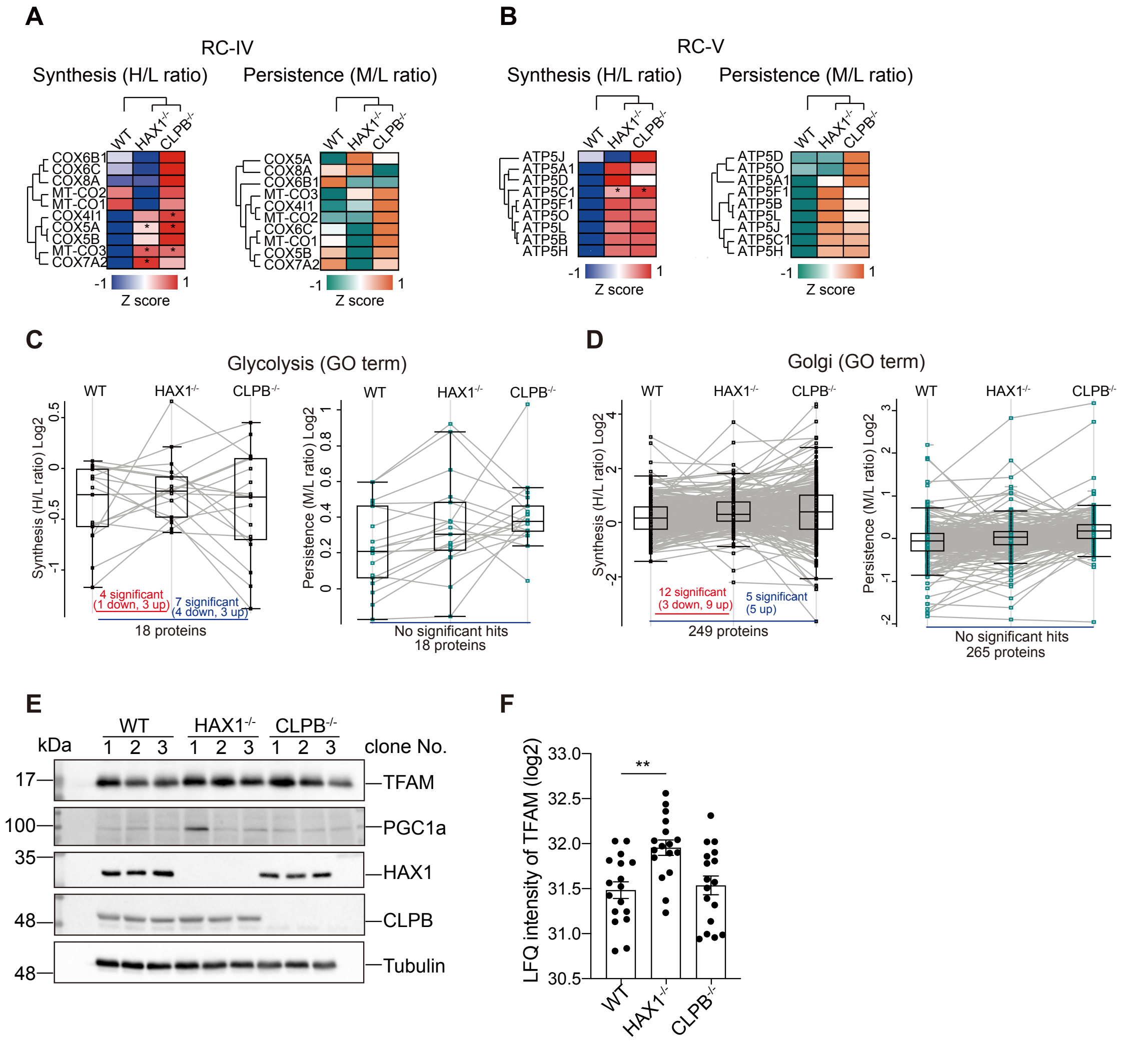

Supplemental Figure 3. Protein synthesis and persistence in mitochondria-dependent and -independent pathways. Related to Figure 3.

(A-B) Unsupervised hierarchical clustering of protein synthesis (H/L, left) and protein persistence (M/L, right) for mitochondrial RC-IV (A) and RC-V (B) in indicated genotypes after 24 hours of pulse with heavy amino acids (Gene Ontology (GO) annotations, n = 6, *P<0.05, Student's t-test). Proteins with 100% valid SILAC ratios were used for the analysis. (C-D) Profile plots of protein synthesis (H/L, left) and protein persistence (M/L, right) for glycolysis (C) or Golgi apparatus (D) in indicated genotypes after 24 hours of pulse with heavy amino acids (Gene Ontology (GO) annotations, n = 6, *P<0.05, Student's t-test,). The number of proteins with significantly different expression (*P<0.05) between WT and each mutant is indicated at the bottom of the plot. The corresponding proteins and their p values are detailed in Supplemental Table 2 and 3. (E) PLB-985 cells of indicated genotypes were lysed and analyzed by immunoblotting. (F) Analysis of LFQ intensity of TFAM (log2, SILAC light) from indicated genotypes (n = 18, *P<0.01, one-way ANOVA followed by Tukey's test).

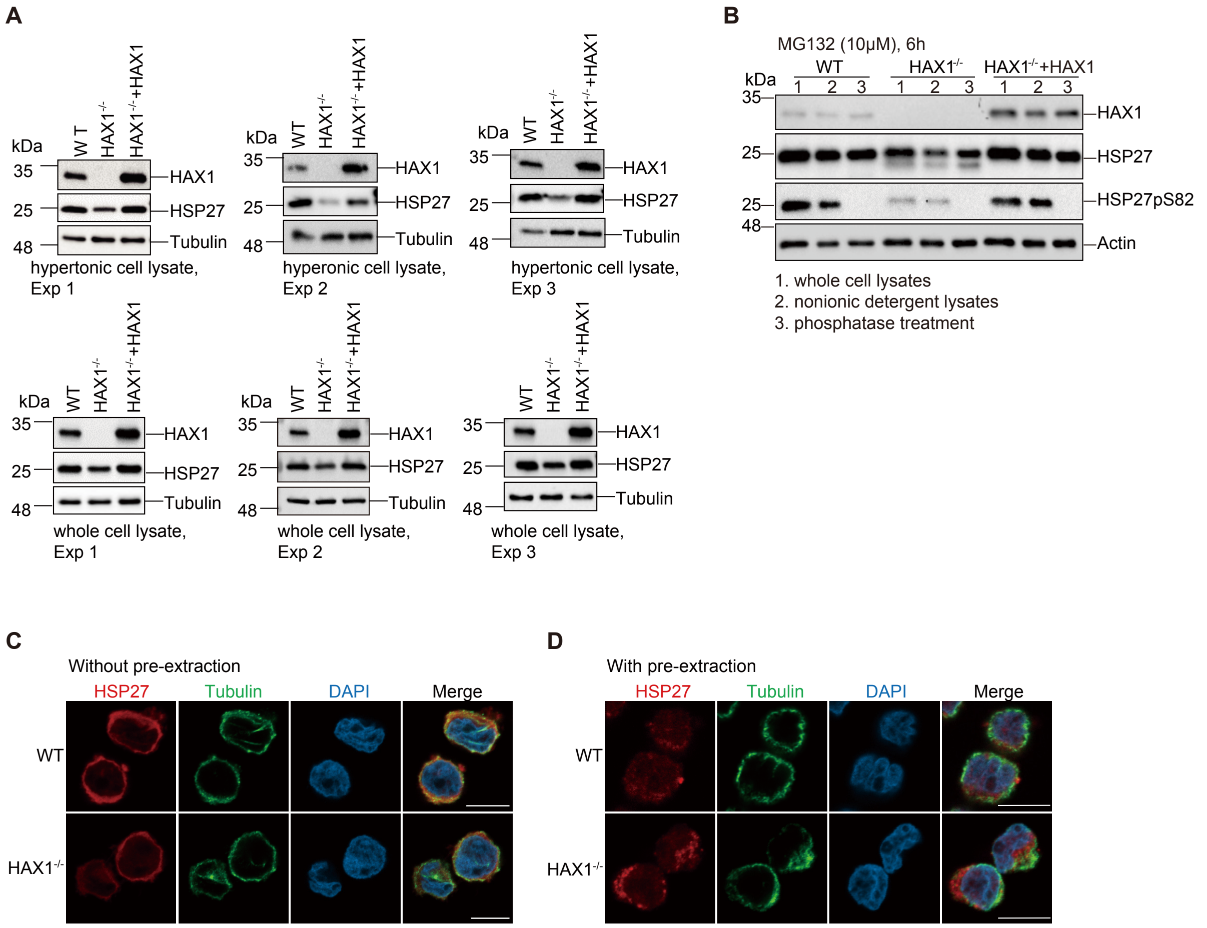

Supplemental Figure 4. HAX1 regulates the phosphorylation state and the solubility of HSP27 in mitochondria. Related to Figure 5.

(A) PLB-985 cells harvested from indicated genotypes were subjected to hypertonic cell lysis (upper panel) or whole cell lysates (lower panel) and analyzed by immunoblotting with indicated antibodies. The immunoblots shown in Experiment 1 upper panel is additionally shown in the main Figure 5B. (B) PLB-985 cells from indicated genotypes were harvested after MG132 treatment (10 μm, 6h). The harvested cell pellets were 1) cooked with Laemmli buffer to obtain whole cell lysates, 2) lysed with nonionic detergents or 3) treated with phosphatase. All samples were analyzed by immunoblotting with the indicated antibodies. (C-D) Representative images of immunostaining of HSP27 and in WT or HAX1$^{-/-}$ PLB-985 cells without (C) or with (D) pre-extraction treatment. DNA was visualized with DAPI. Scale bar, 10 μm.

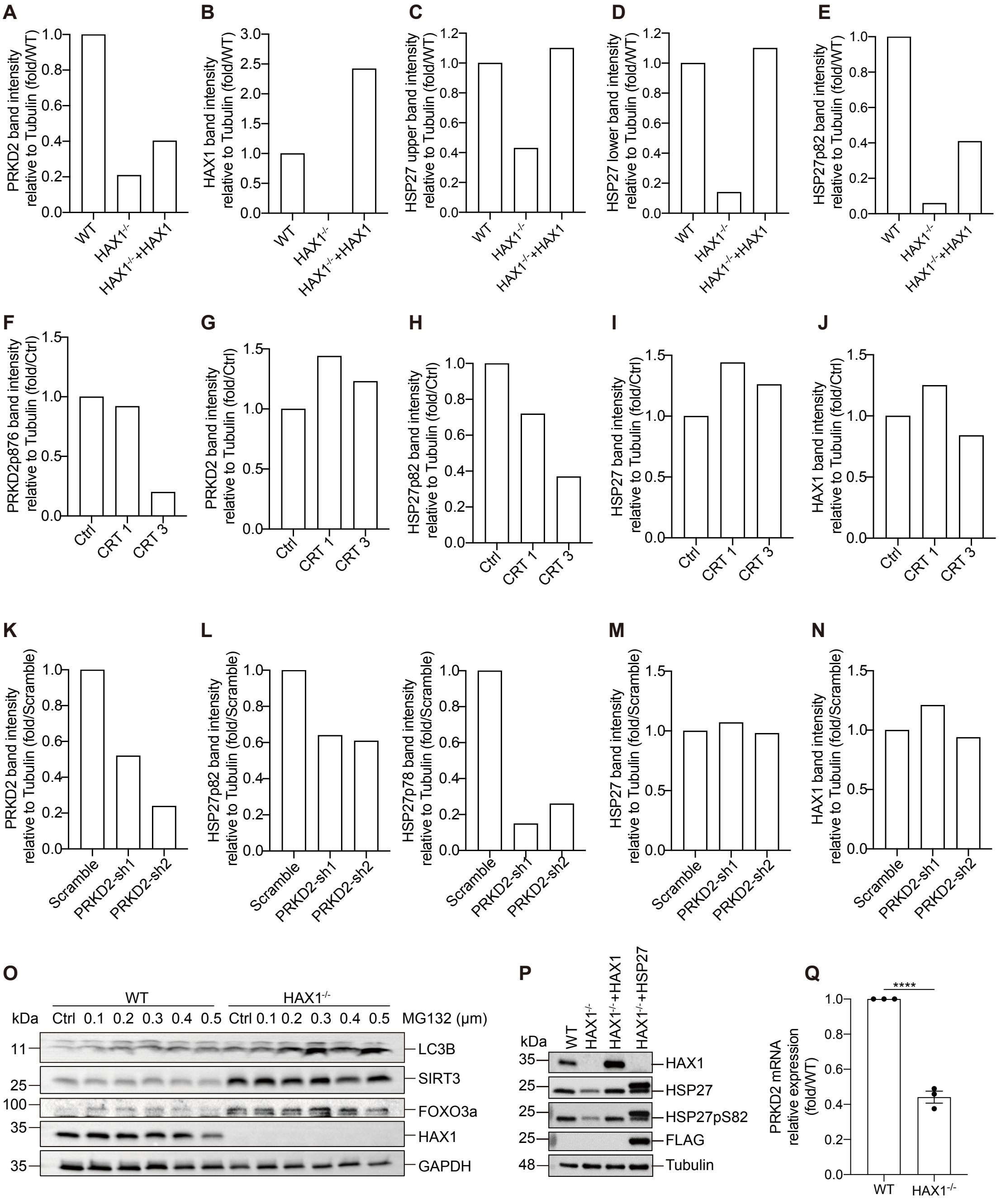

Supplemental Figure 5. PRKD2 is a mitochondrial kinase involved in HSP27 phosphorylation. Related to Figure 6.

(A-E) Analysis of band intensities of indicated proteins in the immunoblotting shown in Figure 6B. (F-J) Analysis of band intensities of indicated proteins in the immunoblotting shown in Figure 6D. (K-N) Analysis of band intensities of indicated proteins in the immunoblotting shown in Figure 6E. (O) Lysates of WT or HAX1$^{-/-}$ PLB-985 cells treated with or without MG132 at indicated concentrations for 18 h were analyzed by immunoblotting with indicated antibodies. (P) Lysates of WT, HAX1$^{-/-}$, HAX1$^{-/-}$+HAX1 or HAX1$^{-/-}$+HSP27 cells were analyzed by immunoblotting with indicated antibodies. (Q) PRKD2 mRNA relative expression in WT and HAX1$^{-/-}$ PLB-985 cells were examined by qPCR (n = 3, ****P<0.0001, unpaired t-test).

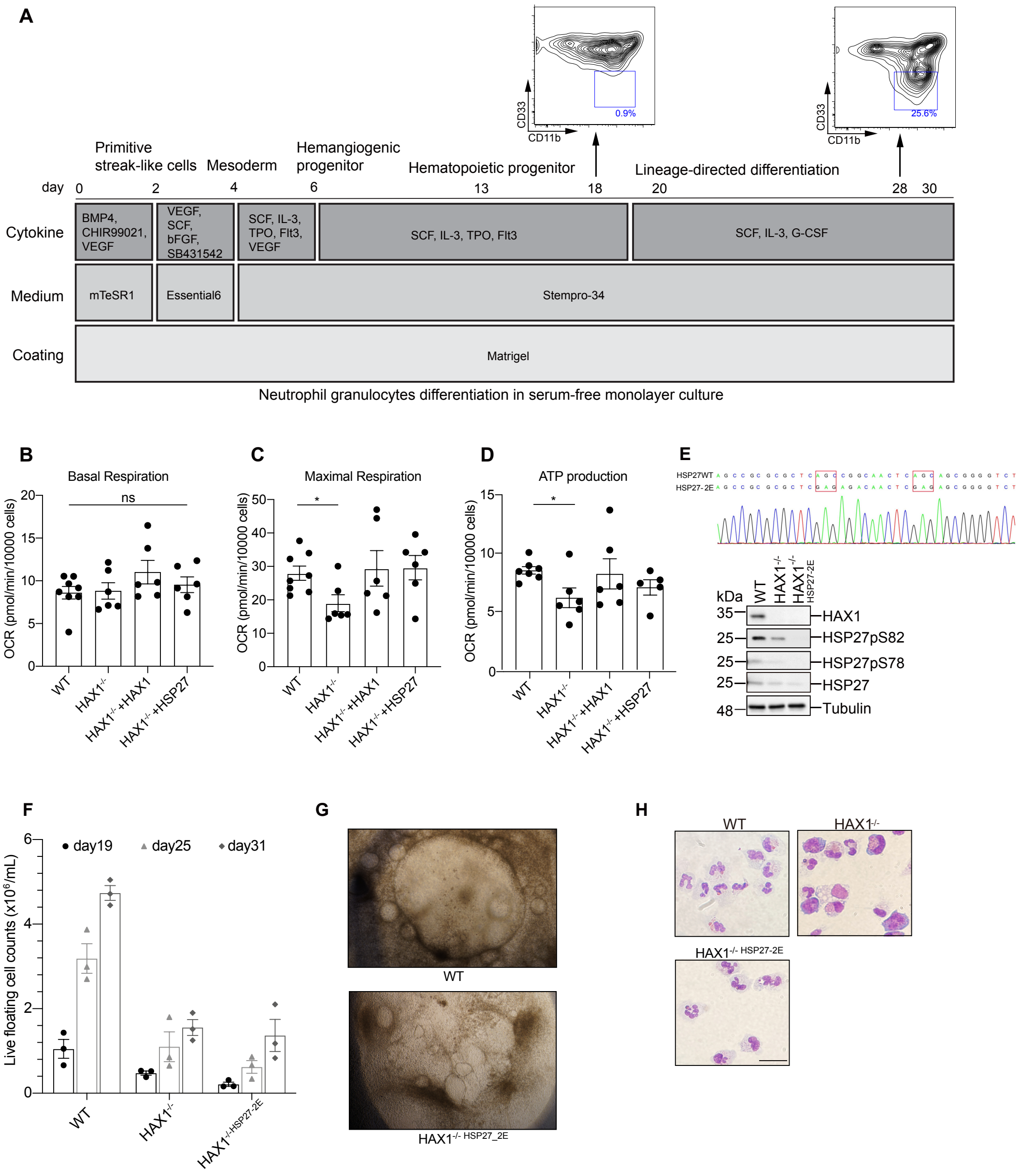

Supplemental Figure 6. HSP27 overexpression restores neutrophil differentiation defect in HAX1$^{-/-}$ iPS cells. Related to Figure 7. (A) Scheme of in vitro differentiation of neutrophil granulocytes. Neutrophil maturation was analyzed with harvested floating cells gated on CD11b^{+}CD33^{low} at day 18 or 28 of differentiation. (B-D) Analysis of Basal OCR, maximal respiratory capacity, and ATP production of hematopoietic stem cells (at day 18 of differentiation) derived from WT, HAX1$^{-/-}$, HAX1$^{-/-}$+HAX1 or HAX1$^{-/-HSP27-2E}$ iPSCs (n = 6, *P<0.05, multiple t test with Holm-Sidak correction). (E) Genomic sequencing of HAX1$^{-/-HSP27-2E}$ iPSCs (upper panel) and immunoblotting of WT, HAX1$^{-/-}$, HAX1$^{-/-HSP27-2E}$ iPSCs (lower panel). (F) Quantification of live floating cells per 6 iPSC colonies (per well), determined at indicated timepoints during differentiation (n = 3, *P < 0.05, multiple t-test). (G) Images represent the colony formation from WT and HAX1$^{-/-HSP27-2E}$ iPSCs at day 28 of differentiation determined by light microscopy (5 x object, Zeiss). (H) Light microscopy of iPSC-derived immature and mature neutrophil granulocytes stained with May-Grünwald Giemsa (×63) at day 28 of differentiation. Scale bar, 10 µm.

Supplemental methods

Whole-exome sequencing and variant filtering

Whole Exome sequencing was performed at the Dr. von Hauner Children's hospital NGS facility. Briefly, genomic DNA from whole blood was used for preparation of whole-exome libraries using the Nextera FLEX (Illumina) with SureSelect XT Human All Exon V5+UTR or V6+UTR kit (Agilent Technologies,) and subsequently sequenced with a NextSeq 500 platform (Illumina) to an average coverage depth of 90x. Bioinformatic analysis used Burrows-Wheeler Aligner (BWA 0.7.15), Genome Analysis ToolKit (GATK 3.6) and Variant Effect Predictor (VEP 89). Frequency filtering was done using Variant alleles were filtered allele for frequencies frequency from against public (e.g. GnomAD, ExAC and GME) and in-house databases. Potentially causative variants were confirmed by Sanger sequencing.

Sanger sequencing

Sanger sequencing of *CLPB* was performed to confirm WES-detected variants and their segregation with the clinical phenotype across the family members. Genomic DNA was PCR-amplified using OneTaq Polymerase (NEB), specific primers are provided in Supplementary Table 5. Amplicons were sequenced either in-house or by using the commercial service of Eurofins Genomics.

iPS cell culture and generation of neutrophil granulocytes

Healthy control fibroblast derived iPS cells were kindly provided from Dr. Drukker (Institute of Stem Cell Research and the Induced Pluripotent Stem Cell Core Facility, Helmholtz Center Munich). Undifferentiated iPS cell colonies were disseminated on 6-well culture plates coated with growth factor-reduced matrigel (Becton-Dickinson) in mTeSR1 medium. BMP4 (80 ng/ml, Peprotech), CHIR99021 (4 µM, Millipore) and VEGF (80 ng/ml, Peprotech) were added to the medium on day 0 of differentiation. Doxycycline (0.05 µg/ml) was added to induce HSP27 or HAX1 protein overexpression. On day 2, medium was replaced by essential 6 medium (Thermo Fisher scientific) supplemented with VEGF (80 ng/ml), basic FGF (25 ng/ml, Peprotech), SCF (50 ng/ml, Peprotech) and SB431542 (2 µM, selleckchem) and doxycycline (0.05 µg/ml). The cytokines were switched to SCF (50 ng/ml), IL-3 (50 ng/ml, Peprotech), Flt-3 (50 ng/ml, Peprotech), TPO (5 ng/ml, Peprotech), VEGF (50 ng/ml), doxycycline (0.05 µg/ml) with StemPro-34 SFM medium (Thermo Fisher scientific) on day 6 for the generation of hemangiogenic progenitors. Meanwhile, 6 iPS colonies at similar size and differentiation status were selected and kept for subsequent differentiation. On day 8, medium was replaced by StemPro-34 SFM medium supplemented with SCF (50 ng/ml), IL-3 (50 ng/ml), Flt-3 (50 ng/ml), TPO (5 ng/ml) and doxycycline (0.05 µg/ml). Thereafter, half medium was changed

every 3-4 days until day 17. On day 17, cytokines were changed to G-CSF (50 ng/ml, Peprotech), SCF (50 ng/ml), IL-3 (50 ng/ml) and doxycycline (0.5 µg/ml). Mature neutrophil-like cells appeared around day 20. Floating live cells were enumerated by Trypan blue staining in a counting chamber.

Establishment of stable inducible expression of HSP27 or HAX1 in HAX1 knockout iPS cells

Reconstitution of HAX1 knockout iPS cells with HSP27 or HAX1 were performed using inducible expression transposon system *PiggyBac*. *HSP27* and *HAX1* cDNA were amplified using a polymerase chain reaction and then inserted into all-in-one *PiggyBac* transposon destination vector for doxycycline-inducible expression (addgene, cat# 80478). HAX1 knockout iPS cells were transfected with these destination vectors carrying *HSP27* or *HAX1* together with the *PiggyBac* transposase expression vector (System Biosciences, cat# PB210PA-1), and positively transported cells were then selected using puromycin (0.5 µg/ml). As a control, we prepared HAX1 knockout iPS cells transfected with an empty *piggybac* construct with the *PiggyBac* transposase vector. After puromycin (0.5 µg/ml) selection, undifferentiated iPSC cells were subsequently stimulated with doxycycline (0.05 µg/ml) for 24 hours prior to immunoblotting for checking the overexpression of HAX1 and HSP27.

sgRNA design and cloning for targeted gene correction in iPS cells

SgRNA-Cas9 with fusion *GFP* plasmid was purchased from Addgene (PX458, cat# 48138). *SgRNAs* targeting *HAX1* (*sgRNA*target *HAX1*: AGTACGAGATTTCAATAGCA) was designed with the online CRISPR Design tool developed by Feng Zhang’s Lab (http://crispr.mit.edu/). *SgRNA* with a lower number of predicted potential off target sites in coding region were selected. *SgRNA-Cas9* vector was transfected into healthy control-derived iPS cells using Amaxa Nucleofector® II Device (Lonza, program B-016) with solutions indicated in Human stem cell nucleofector kit 2 (Lonza, cat# VPH-5022). *pCas-Guide* vector with a scrambled sequence was used as control. Two days after transfection, GFP positive cells were sorted by BD FACS Aria (BD Biosciences) and 3000 iPS cells were seeded on a 10 cm dish pre-coated with mouse embryonic fibroblasts. 10 days after seeding, each colony derived from a single cell was picked up for genotyping and further expansion.

FACS analysis and sorting

All antibodies used for FACS analysis are listed in the Supplemental Table 6. Upon doxycycline (0.05 µg/ml) induction, floating neutrophil-like cells were collected and shortly washed with PBS prior to staining with FACS antibodies. Mature neutrophil population was

identified by gating with (Live/mcheery^{+}/CD33^{low}/CD11b^{+}) prior to further FACS analysis. FACS data were analyzed using FlowJo Software (TreeStar) v9 and v10.

Cytospin and light-microscopy

2 × 10^4 differentiated iPS cells were centrifuged at 500 rpm at RT for 5 min utilizing a Tharmac Cellspin I Cytocentrifuge (THARMAC) on to air-dried slides. The prepared cytospin slides were stained with May-Gruenwald Giemsa (Merck, cat# 1014241000) for 2 min, following Giemsas Azur-Eosin-Methylene blue solution (Merck, cat# 1092040500, diluted 1:20 in Soerengen buffer) for 17 min. Distribution of the maturation stages and morphology of cells were analyzed with a Zeiss Axioplan 2 imaging microscope (Carl Zeiss).

Molecular Cloning

The coding regions of genes used in this study were amplified from homemade human cDNA and were inserted into vectors summarized in the Supplemental Table 9. *PRKD2* was amplified from *pDONR233-PRKD2* vector ordered from addgene. All primers have been synthetized by Eurofins Genomics and are listed in Supplement Table 5. All plasmids were sequence confirmed.

Cell transfection and sorting

4 µg of *sgRNA-Cas9-GFP/RFP* plasmid and 10 µg polyethylenimine (1) (linear, MW25000) were incubated in 100 µl Opti-MEM for 5 min respectively. The mixture was incubated at room temperature for 20 min. Afterwards, the mixture containing plasmids and PEI was dropped onto 2.5 × 10^5 HeLa cells cultured in the well of 6-well plate. Suspension cells (3-5 × 10^6) were resuspended in 100 µl of provided kit buffer and mixed with 4 µg of *sgRNA-Cas9-GFP/RFP* plasmids before transferred to a sterile 0.2-cm cuvette (Lonza, cat# VVCA-1003). The electroporation was performed with T-019 program by the Lonza® Nucleofector® II electroporation system. After electroporation, cells were gently resuspended in pre-warmed RPMI complete medium and then seeded in 12-well plates. The medium was replaced after 8 h. After 2 days, cells were harvested and GFP/RFP positive cells were sorted into 96-well plates by single-cell sorting by FACSAria. At an appropriate size, single colonies were harvested for immunoblotting. Knock-out clones were validated by immunoblotting and genomic sequencing.

Stable transfection

In order to confer stable overexpression of indicated genes (*HAX1, CLPB and HSP27*), lentiviral vector pRRL was applied for gene delivery. First, genes of interest were cloned via SpeI and AgeI restriction sites into pRRL vector. For lentivirus production, 4 × 10^6 HEK293T

cells were seeded in 10 cm dish 12 h before transfection via PEI. Cells were subsequently transfected with lentiviral vector, *gag-pol*, *VSVG* and *rev* helper plasmids. Supernatants containing viral particles were collected every 24 h for 72 h. PLB-985 cells were stably transduced, GFP sorted and expanded. Overexpression efficiency was analyzed by immunoblotting.

Inhibitor studies

To inhibit the activity of PRKD family in PLB-985 cells, cells were treated with 1 μM or 3 μM CRT0066101 for 3 h at 37°C prior to analysis by SDS-PAGE and immunoblotting.

Mitochondrial isolation

PLB-985 or HeLa cells were respectively harvested and homogenized in TH buffer (300 mM Trehalose, 10 mM KCl and 10 mM Hepes, pH 7.4) with 0.1 mg BSA/mL, by a Potter S homogenizer (Sartorius). Homogenized cell fractions were centrifuged at 400× g, 4°C for 10 min and supernatants were kept as a preliminary postnuclear fraction. Cells were homogenized twice and supernatants collected from each time were centrifuged at 800× g, 4°C for 5 min to obtain a pure postnuclear fraction. This fraction was subsequently pelleted by centrifugation at 10,000× g for 10 min at 4°C. Freshly isolated mitochondria were washed in BSA-free TH buffer and protein concentrations were determined by Bradford analysis using bovine serum albumin (Sigma- Aldrich, A7030) as a standard.

Mitochondrial swelling experiment

Protocols for swelling human mitochondria were modified according to (David U. Mick et al., 2012). In brief, isolated mitochondrial were either osmotically stabilized in SEM buffer (250 mM sucrose, 1 mM EDTA, and 10 mM MOPS, pH 7.2) or ruptured the outmembrane by EM buffer (1 mM EDTA and 10 mM MOPS, pH 7.2) or fully broken via sonification, prior to treatment of protease K for 10 min at 4°C. The degradation reactions were stopped by addition of 1 mM PMSF to each sample for another 10 min, at 4°C. Samples from SEM and EM treatment were further subjected to ultracentrifugation and remained pellets were resuspended with 1 × loading dye, cooked at 95°C, 5 min. Mitochondrial isolation is described above.

Mitochondrial carbonate extraction

Mitochondria isolated from indicated cell lines were solubilized with 0.1 M Na_2CO_3 at pH 10.8 or pH 11.5. As a control, mitochondria were resuspended with 1% Triton buffer on ice. After 20 min incubation on ice, mitochondrial membranes were pelleted by ultracentrifugation at

45,000 rpm, 4°C for 45 min. All samples were precipitated with trichloroacetic acid and washed with ice cold acetone before analyzed by immunoblotting.

Immunoprecipitation with Anti-FLAG M2 agarose beads

Wild-type and mutant *HAX1* or *CLPB* Flag-tagged plasmids were transfected into semi-confluent HEK293T cells with 10 µg plasmid/10 cm dish. After 48 h, cells were pelleted and lysed in freshly prepared RIPA Buffer (450 mM NaCl, 25 mM Tris-HCl pH 7.5, 1 mM EDTA, 1% NP40, 5% Glycerol, 25 mM Na-Pyrophosphate, 50 mM Na-Fluoride, EDTA-free protease inhibitor (Roche)). The supernatant was collected and remaining debris were removed by centrifugation of 14,000 rpm, at 4°C for 15 min. Subsequently, the supernatant was incubated with anti-FLAG M2 Affinity gel (Sigma, cat# A2220) overnight at 4°C on a rotating laboratory wheel. Beads were pelleted and washed 5 times in RIPA buffer before analyzed by immunoblotting.

Immunofluorescence studies

Fixed cells were shortly washed with PBS and quenched in 50 mM NH_4Cl for 30 min. Afterwards, cells were permeabilized with 0.2% Trion for 2 min at RT prior to blocking with 0.02% Triton and 1% BSA in TBS (w/v). Coverslips were then incubated with indicated primary and secondary antibodies for 1 h at RT before DAPI staining. Nuclear staining was conducted using 4'6-diamidino-2-phenylindole (DAPI). Coverslips were mounted on glass slides using aqueous mounting medium (Dako Cytomation), before examined by confocal microscopy. For pre-extraction step, cells were permeabilized with pre-extraction buffer (0.5% Triton X-100 in 5 mM $MgCl_2$) for 1 min at RT prior to cell fixation. For staining of monoclonal antibodies, cells were cooked in antigen retrieval buffer after the quenching step. Confocal microscopy was performed with a Zeiss LSM-800 upright microscope using a 63x 1.4NA Oil DIC Plan-Apochromat immersion lens. Detailed pre-extraction method was described in Supplemental method.

Interactome analysis of HSP27

HEK293T cells expressing either pRRL or $HSP27^{FLAG}$ were lysed in freshly prepared mitochondrial IP Buffer (100 mM NaCl, 50 mM TrisHCl pH 7.5, 10 mM $MgCl_2$, 10% Glycerol, 1% digitonin, 25 mM Na-Pyrophosphate, 50 mM Na-Fluoride, EDTA-free protease inhibitor (Roche)) respectively. The supernatant from each sample was collected and remaining debris were removed by centrifugation of 14,000 rpm, at 4°C for 15 min. Subsequently, the supernatant was incubated with anti-FLAG M2 agarose beads for 1.5 h at 4°C on a rotating wheel. Beads were pelleted and washed 5 times in mitochondrial IP washing buffer (0.1% digitonin) prior to elution with 0.1 M glycine at pH 3.0, on a thermo shaker for 5 min at RT.

Protein eluates were precipitated with cold acetone and resuspended in LYSE buffer (PreOmics). Bound proteins were further digested and analyzed by mass spectrometry.

Measurement of mitochondrial complexes activity

The activities of the mitochondrial complexes I and IV were assessed by the Complex I Enzyme Activity Microplate Assay Kit (abcam) and the Complex IV Human Specific Activity Microplate Assay Kit (abcam) respectively. Mitochondria adopted for the measurements were harvested on the same day.

Measurement of mitochondrial ROS

ROS were measured using MitoSOX Red mitochondrial superoxide indicator (Molecular Probes, Invitrogen) according to the manufacturer instructions. Cells were incubated with 3 µM MitoSOX at 37°C for 10 min, briefly washed with PBS, and resuspended before being processed by fluorescence- activated cell sorting (FACS Canto II, BD Biosciences) at an excitation/emission of 510/580 nm.

In-gel-mass spectrometry

Mass spectrometry of Supplemental Figure. 1 was performed as previously described (2). After immunoprecipitation of $HAX1^{FLAG}$, the eluate was subjected to SDS-PAGE and coomassie stained bands were cut out and in-gel digested. Gel pieces were processed as described in Schulz et al. and proteins digested with trypsin (20 ng/µl) at 37°C overnight. To extract the peptides, a 0.1% trifluoroacetic acid (TFA) and reverse-phase chromatography (EASY-nLC; Bruker Daltonics) was used, with a PepMap100 C18 nano-column (Dionex). Peptides were eluted by a gradient from 9.5-90.5% acetonitrile in 0.1% TFA for 80 min and mixed with a-cyano-4-hydroxycinnamic acid (HCCA) as matrix (4.5% of saturated HCCA in 90% acetonitrile, 0.1% TFA, 1 mM $NH_4H_2PO_4$). The samples were spotted onto an anchorchip target using a robot (Bruker Daltonics) and further analyzed in a MALDI-TOF/TOF mass spectrometer (Ultraflextreme; Bruker Daltonics) recording MS and post-source decay MS/MS spectra using the software as previously described (Bruker Daltonics) (2).

SILAC labelling

Cells were cultured in RPMI 1640 media lacking two amino acids (lysine and arginine) (RPMI 1640 media for SILAC, Life technologies) with dialyzed FBS. The medium was supplemented with either “light” standard unlabelled Lysine and Arginine or “medium” isotope labelled $^{13}C_4$ -– Lysine (Lys4) and $^{13}C_6$ -– Arginine (Arg6) (Silantes, Munich, Germany). Cells were cultured for more than 10 passages in SILAC medium and their incorporation of “medium” amino acids was confirmed by MS analysis. For the pulse experiment with heavy SILAC amino acids, cells

cultured in “medium” amino acids were washed and incubated with “heavy”-isotope labelled $^{13}C_6{}^{15}N_2$ — Lysine (Lys8) and $^{13}C_6{}^{15}N_4$ — Arginine (Arg10) (Silantes) for 24 hours (see Figure. 3A). At time 0, 6 hours and 24 hours, pulsed cells were collected and counted using cell counting chamber. 2 × 10^6 PLB-985 cells of “heavy”-pulsed cells were mixed with the same number of cells cultured in “light” medium (3). 1 × 10^6 “heavy”-pulsed+ “light” cells were washed with PBS and pelleted at 1200 rpm for 3 minutes and snap-frozen. 3 × 10^6 “heavy”-pulsed+ “light” cells were further processed for the isolation of mitochondria (see above) which were subsequently snap-frozen. Data were obtained in three independent cell clones per genotype in two entirely independent biological replicates corresponding to two rounds of SILAC labeling.

Lysis and protein digestion for mass spectrometry

Pellets of cells and mitochondria were lysed in 40 µl of LYSE buffer (preOmics, Martinsried, Germany), heated at 95°C for 5 min and sonicated using a water-bath sonicator (Diagenode) for 8 min with a 50% duty cycle. Protein content in the lysates was determined by comparison to a Tryptophan protein standard using a spectrophotometric method, with excitation wavelength 280 nm and emission wavelength 350 nm. For each sample, 10 µg of protein were digested in LYSE with 1 µg of endoproteinase LysC and 1 µg of trypsin at 37°C overnight under continuous stirring. Peptides were acidified to a final concentration of 0.1% trifluoro-acetic acid (TFA) and loaded onto StageTip plugs of styrene divinylbenzene reversed-phase sulfonate (SDB-RPS). Purified peptides were eluted with 80% acetonitrile-1% ammonia and dried. The amount of recovered peptide in each sample was measured using NanoDrop 2000 Spectrophotomether (Thermo Scientifics).

Data Acquisition and Computational proteomics

Eluted peptides (0.5 µg/sample) were separated on a reverse phase 50-cm column with 75-µm inner diameter, packed in-house with 1.8-µm C18 particles (Dr Maisch GmbH, Germany) kept at 60°C by a column oven (Sonation, Biberach, germany) controlled by the SprayQC software (4). Liquid chromatography performed on an EASY-nLC 1200 ultra-high-pressure system coupled through a nanoelectrospray source to a Q Exactive HF-X mass spectrometer (all from Thermo Fisher Scientific). Peptides were loaded in buffer A (0.1% [v/v] formic acid), applying a 100 minutes nonlinear gradient of 5% - 98% buffer B (0.1% [v/v] formic acid and 80% [v/v] acetonitrile) at a flow rate of 350 nL/minute. Data acquisition switched between a full scan and 15 data-dependent MS/MS scans. Multiple sequencing of peptides was minimized by excluding the selected peptide candidates for 40 seconds.

The MaxQuant software (version 1.6.1.13) was used for the analysis of raw files (5). Peak lists were searched against the human UniProt FASTA database version of 2018 (21042 protein

entries) and a common contaminants database (247 entries) using the Andromeda search engine (6). False discovery rate was set to 1% for peptides (minimum length of 7 amino acids) and proteins and was determined by searching a reverse database. A maximum of two missed cleavages were allowed in the database search. Peptide identification was performed with an allowed initial precursor mass deviation up to 7 ppm and an allowed fragment mass deviation of 10 ppm. The “Match between runs” option in MaxQuant was activated.

Statistical analysis of mass-spectrometry data

Data analysis was performed with the Perseus software (version 1.5.4.2) embedded in the MaxQuant environment (7). For the SILAC experiment, data are the median of three independent clones and two biological replicates (n = 6). For the HSP27 pulldown, two independent experiments were carried out, each with three independent pulldowns for both HSP27 and control (GFP) antibody. Only proteins quantified in both experiments were used for the analysis. Categorical annotations were supplied in the form of UniProt Keywords, KEGG and Gene Ontology. SILAC ratios and the label free quantification (LFQ) intensity of “light”-labelled samples were used for data analysis. Annotation enrichments were derived by Fischer’s exact test, using Benjamini-Hochberg FDR for truncation and a threshold value of 0.02. All volcano plots shown are based on SILAC light LFQ intensity. Enrichments in proteins in the HSP27 pulldown (right side of the volcano plot) were calculated using the annotated human proteome as background list. To determine the lists of proteins significantly changing among wild type, HAX1- and CLPB-deficient clones we performed Student’s t test using 0.05 FDR for truncation and 250 randomizations. Hierarchical clustering was performed on SILAC values (Log2) after Z scoring. Data were filtered for 3 valid values in total, unless indicated otherwise in the figure legend.

Supplemental Tables

Supplemental Table 1. List of proteins identified in complex with HAX1

Gene symbol	Name	No. of peptides matched	% sequence coverage	Mass	Score
ASAP2_HUMAN	Arf-GAP with SH3 domain, ANK repeat and PH domain-containing protein 2	1	1	111581	33
DCD_HUMAN	Dermcidin	1	1	11277	46
HSP7C_HUMAN	Heat shock cognate 71 kDa protein	2	2	70854	97
HSP76_HUMAN	Heat shock 70 kDa protein 6	1	1	70984	50
ZN616_HUMAN	Zinc finger protein 616	1	1	90215	38
CLPB_HUMAN	Caseinolytic peptidase B protein homolog	21	17	78680	1080
STXB4_HUMAN	Syntaxin-binding protein 4	1	1	61623	41
ANR11_HUMAN	Ankyrin repeat domain-containing protein 11	3	3	297731	36
SHRM3_HUMAN	Protein Shroom3	1	1	216714	34
QRIC2_HUMAN	Glutamine-rich protein 2	1	1	180715	33
STML2_HUMAN	Stomatin-like protein 2, mitochondrial	5	5	38510	344
HERC2_HUMAN	E3 ubiquitin-protein ligase HERC2	2	2	526895	43
HORN_HUMAN	Hornerin	5	5	282228	357
HAX1_HUMAN	HCLS1-associated protein X-1	3	3	31601	282
PLAK_HUMAN	Junction plakoglobin	2	2	81693	146

Gene symbol	Name	No. of peptides matched	% sequence coverage	Mass	Score
PLIN5_HUMAN	Perilipin-5	3	3	50760	46
SHRM2_HUMAN	Protein Shroom2	3	3	176303	39
CILP1_HUMAN	Cartilage intermediate layer protein 1	2	2	132480	34
HYDIN_HUMAN	Hydrocephalus-inducing protein homolog	3	3	575528	32
C2D2A_HUMAN	Coiled-coil and C2 domain-containing protein 2A	2	2	186070	32
PGAM5_HUMAN	Serine/threonine-protein phosphatase PGAM5	2	2	31985	144
FRAS1_HUMAN	Extracellular matrix protein FRAS1	1	1	442928	25

Supplemental Table 1. List of proteins identified in complex with HAX1. Related to Figure 1.

List of proteins identified in complex with HAX1. 22 proteins identified as potential binding partners of HAX1 by mass spectrometry. The columns correspond to their gene symbol, name, number of unique peptides matched, percent sequence coverage, estimated MW and scores for binding with HAX1 in LC-MS/MS analysis.

Supplemental Table 2. The synthesis of proteins related to the Glycolysis pathway

Gene names	$HAX1^{-/-}$ vs WT	P value	Change $HAX1^{-/-}$	$CLPB^{-/-}$ vs WT	P value	Change $CLPB^{-/-}$
ADPGK	*	0.0036	up		0.692	up
ALDOA		0.1453	down	*	0.037	down
ALDOC		0.6892	up	*	0.035	up
GPI		0.1103	down	*	0.011	down
OGDH	*	0.0176	up	*	0.005	up
PFKL	*	0.0072	up	*	0.006	up

PGAM1		0.1272	down	*	0.034	down
TPI1	*	0.0024	down	*	0.038	down

Supplemental Table 2. The synthesis of proteins related to the Glycolysis pathway. Related to Supplemental Figure 3.

Proteins related to Glycolysis were selected based on Gene Ontology (GO) annotations. Proteins with significantly different expression (*P<0.05) between WT and HAX1$^{-/-}$ or WT and CLPB$^{-/-}$ are listed.

Supplemental Table 3. The synthesis of proteins related to Golgi pathway

Gene names	HAX1$^{-/-}$ vs WT	P value	Change HAX1$^{-/-}$	CLPB1$^{-/-}$ vs WT	P value	Change CLPB$^{-/-}$
ARHGEF2	*	0.0013	up		0.01835	
ARID3A	*	0.0043	down		0.13937	
CYBB		0.1796		*	0.00023	up
DPP7		0.0303		*	0.00115	up
HLA-C		0.0204		*	6.1E-05	up
LIG1	*	0.0026	up		0.73633	
MSH6	*	0.0023	up		0.03504	
NCSTN	*	0.0015	up		0.02609	
QPCTL		0.0978		*	0.00066	up
RAF1	*	0.0022	up		0.01250	
SAR1A	*	0.0021	up		0.00949	
SCAMP2	*	0.0029	up		0.09975	
TMEM214	*	0.0054	up		0.04037	
TMEM43	*	0.0005	up	*	0.00113	up
TSNAX	*	0.0007	down		0.94368	
YKT6	*	0.0029	down		0.96969	

Supplemental Table 3. The synthesis of proteins related to Golgi pathway. Related to Supplemental Figure 3.

Proteins related to Golgi apparatus were selected based on Gene Ontology (GO) annotations. Proteins with significantly different expression (*P<0.05) between WT and HAX1$^{-/-}$ or WT and CLPB$^{-/-}$ are listed.

Supplemental Table 4. Subunits of mitochondrial complexes identified in the HSP27 IP

Protein ID	Protein name	Gene name
O95299	NADH dehydrogenase [ubiquinone] 1 alpha subcomplex subunit 10	NDUFA10
Q9P0J0	NADH dehydrogenase [ubiquinone] 1 alpha subcomplex subunit 13	NDUFA13
Q16718	NADH dehydrogenase [ubiquinone] 1 alpha subcomplex subunit 5	NDUFA5
A0A0C4DGS0	NADH dehydrogenase [ubiquinone] 1 alpha subcomplex subunit 6	NDUFA6
P51970	NADH dehydrogenase [ubiquinone] 1 alpha subcomplex subunit 8	NDUFA8
Q16795	NADH dehydrogenase [ubiquinone] 1 alpha subcomplex subunit 9	NDUFA9
Q9Y375	Complex I intermediate-associated protein 30	NDUFAF1
Q9P032	NADH dehydrogenase [ubiquinone] 1 alpha subcomplex assembly factor 4	NDUFAF4
O96000	NADH dehydrogenase [ubiquinone] 1 beta subcomplex subunit 10	NDUFB10
O95168	NADH dehydrogenase [ubiquinone] 1 beta subcomplex subunit 4	NDUFB4
P28331	NADH-ubiquinone oxidoreductase 75 kDa subunit, mitochondrial	NDUFS1
O75306	NADH dehydrogenase [ubiquinone] iron-sulfur protein 2	NDUFS2
O75489	NADH dehydrogenase [ubiquinone] iron-sulfur protein 3	NDUFS3
P56181	NADH dehydrogenase [ubiquinone] flavoprotein 3	NDUFV3
P31040	Succinate dehydrogenase [ubiquinone] flavoprotein subunit	SDHA
P21912	Succinate dehydrogenase [ubiquinone] iron-sulfur subunit	SDHB
P08574	Cytochrome c1, heme protein, mitochondrial	CYC1
Q9UDW1	Cytochrome b-c1 complex subunit 9	UQCR10
P14927	Cytochrome b-c1 complex subunit 7	UQCRB
P31930	Cytochrome b-c1 complex subunit 1	UQCRC1

P22695	Cytochrome b-c1 complex subunit 2	UQCRC2
P47985	Cytochrome b-c1 complex subunit Rieske	UQCRFS1
P07919	Cytochrome b-c1 complex subunit 6	UQCRH
O14949	Cytochrome b-c1 complex subunit 8	UQCRQ
P13073	Cytochrome c oxidase subunit 4 isoform 1	COX4I1
P20674	Cytochrome c oxidase subunit 5A	COX5A
P14406	Cytochrome c oxidase subunit 7A2	COX7A2
P15954	Cytochrome c oxidase subunit 7C	COX7C
P00403	Cytochrome c oxidase subunit 2	MT-CO2
P00414	Cytochrome c oxidase subunit 3	MT-CO3
P25705	ATP synthase subunit alpha	ATP5A1
P06576	ATP synthase subunit beta, mitochondrial;ATP synthase subunit beta	ATP5B
P36542	ATP synthase subunit gamma, mitochondrial	ATP5C1
Q5QNZ2	ATP synthase F (0) complex subunit B1, mitochondrial	ATP5F1
P48047	ATP synthase subunit O, mitochondrial	ATP5O
Q06055	ATP synthase F (0) complex subunit C1, mitochondrial;ATP synthase F(0) complex subunit C2, mitochondrial;ATP synthase F(0) complex subunit C3, mitochondrial	ATP5G

Supplemental Table 4. Subunits of RCs identified in the HSP27 IP. Related to Figure 6. List of subunits of RCs identified in complex with HSP27. The columns correspond to protein ID, protein name and gene name.

Supplemental Table 5. Oligonucleotides used in this study

Primers	**Forward**	**Reverse**
Plasmids		
pRRL-HAX1	GAACCGGTGCCACCATG AGCCTCTTTGATCTCTTC CGGGG	GGACTAGTCTACCGGGACCGG AACCAACGTC
pRRL-HAX1-cFlag	GAACCGGTGCCACCATG AGCCTCTTTGATCTCTTC CGGGG	GGACTAGTCTACTTGTCGTCAT CGTCTTTGTAGTCCCGGGACC GGAACCAACGTC

pRRL-HAX1Δ1-cFlag	GAACCGGTGCCACCATGCACAGAGATCCCTTTTTTGGAGGG	GGACTAGTCTACCGGGACCGGAACCAACGTC
pRRL-HAX1Δ2'-OL-cFlag	ACCTCGGAGCAGACTACGGGAGGGACAGACACTTC	CCCGTAGTCTGCTCCGAGGTCCAGGAAAGCCGAAA
pRRL-HAX1Δ3-OL-cFlag	CCATCCTCCTTTTGATGATGTATGGCCTATGGACC	CATCATCAAAAGGAGGATGGGAAGGCAAGGTCCAG
pRRL-HAX1Δ5-OL-cFlag	AGAGGACAATATAGTGGAGGAGCGCCGGACTGTGG	CCTCCACTATATTGTCCTCTCTGGTTCTAGGATGG
pRRL-HAX1Δ6+7-cFlag	GAACCGGTGCCACCATGAGCCTCTTTGATCTCTTCCGGGG	GGACTAGTCTACTTGTCGTCATCGTCTTTGTAGTCCCCATCTGGTTTAGTGATCTTGGTC
pRRL-HAX1Δ118-168-OL-cFlag	ACCTGGTGAGTTTGATGATGTATGGCCTATGGACC	CATCATCAAACTCACCAGGTGTCTCTGACTCAGGA
pRRL-HAX1Δ123-168-OL-cFlag	ACGGGAGGGATTTGATGATGTATGGCCTATGGACC	CATCATCAAATCCCTCCCGTAGTCTCTCACCAGGT
pRRL-HAX1Δ126-168-OL-cFlag	ACAGACACTTTTTGATGATGTATGGCCTATGGACC	CATCATCAAAAAGTGTCTGTCCCTCCCGTAGTCTC
pRRL-HAX1Δ137-168-OL-cFlag	AGATAGTCACTTTGATGATGTATGGCCTATGGACC	CATCATCAAAGTGACTATCTGGATACTTAAGCATT
pRRL-HAX1Δ144-168-OL-cFlag	CTTTGGGGGGTTTGATGATGTATGGCCTATGGACC	CATCATCAAACCCCCCAAAGATCCTGGGCTGGTGA
pRRL-HAX1Δ155-168-OL-cFlag	TGAATCCCCCTTTGATGATGTATGGCCTATGGACC	CATCATCAAAGGGGGATTCACTTCTTGCATCACTC
pRRL-HAX1Δ163-168-OL-cFlag	CTGGGGCTCCTTTGATGATGTATGGCCTATGGACC	CATCATCAAAGGAGCCCCAGTCTGGTGCTGGTTGG

pRRL-HAX-L130R	AAGTATCCAGATAGTCACCAGCCCA	ACGCATTGAGTCCCGAAGTGTCTGT
pRRL-CLPB	GAACCGGTGCCACCATGCTGGGGTCCCTGGTG	GGACTAGTCTAGATGGTGTTGCACACCTTC
pRRL-CLPB-cFlag	GAACCGGTGCCACCATGCTGGGGTCCCTGGTG	GGACTAGTCTACTTGTCGTCATCGTCTTTGTAGTCGATGGTGTTGCACACCTTC
pRRL-CLPBΔ79-cFlag	GAACCGGTGCCACCATGACCAAATGCCTCGCGGCTGCCACTT	GGACTAGTCTACTTGTCGTCATCGTCTTTGTAGTCGATGGTGTTGCACACCTTC
pRRL-CLPBΔANK1-4-OL-cFlag	GAGTCCGTCCAAGCGTGAGGCTGAGGAGCGGCGCC	CCTCACGCTTGGACGGACTCTTGCTGTAGCAATGA
pRRL-CLPBΔTATP-OL-cFlag	CTGGTACGATGTCTACTTCCTCCCCTTCTGCCACT	GGAAGTAGACATCGTACCAGCCATTCTCCTTCCTC
pRRL-CLPBΔCTD2-OL-cFlag	CTACTTCCTCACGGTGGAGGACTCAGACAAGCAGC	CCTCCACCGTGAGGAAGTAGACGATCTCATTGATC
pRRL-CLPB-T268M	AAGGGCTGCATGGCCTTGCAC	GAAACTGGCGCGGTTGTTC
pRRL-CLPB-Y272C	GCCTTGCACTGTGCTGTTCTTG	CGTGCAGCCCTTGAAACT
pRRL-CLPB-R408G	GGGCTTCATCGGGCTGGACAT	TTTTTAGCATCTTTGTGCATATATTTGGC
pRRL-CLPB-R561Q	TTTCTGGGACAGATCAATGAGATCG	CTCATCCCTCCGGAAGTG
TRMPVIR-97mer-shRNA	CAGAAGGCTCGAGAAGGTATATTGCTGTTGACAGTGAGCG	CTAAAGTAGCCCCTTGAATTCCGAGGCAGTAGGCA
pRRL-HSP27-cflag	GAACCGGTGCCACCATGACCGAGCGCCGCGTCCCCTTC	GGACTAGTTTACTTGTCGTCATCGTCTTTGTAGTCCTTGGCGGCAGTCTCATCGG
pENTR-HSP27	ACGCGTCGACGCCACCATGACCGAGCGCCGCGTCCC	CCGGAATTCGTTACTTGGCGGCAGTCTCATCGG

pENTR-HAX1	ACGCGTCGACGCCACCATGAGCCTCTTTGATCTCTTCCGG	CCGGAATTCGCTACCGGGACCGGAACCAACGTC
CLPB_Exon 15	CTTCCCCTGGTGTCTGGTAAT	TTTCCATCTCTTGCCACTCTG
CLPB_Exon 16	ATAAAAGGTGGAGCACAGTGG	TATAGGGAGGCAGGCTGAGAG
97mer shRNA		
Scrambled shRNA	TGCTGTTGACAGTGAGCGCGTAGCGACTAAACACATCAAATAGTGAAGCCACAGATGTATTTGATGTGTTTAGTCGCTACTTGCCTACTGCCTCGGA	
PKD2-shRNA1	TGCTGTTGACAGTGAGCGCCACGACCAACAGATACTATAATAGTGAAGCCACAGATGTATTATAGTATCTGTTGGTCGTGTTGCCTACTGCCTCGGA	
PKD2-shRNA2	TGCTGTTGACAGTGAGCGCTCCCAGCAATGAACTGTTCTATAGTGAAGCCACAGATGTATAGAACAGTTCATTGCTGGGATTGCCTACTGCCTCGGA	
sgRNA target (sequence 5’-3’)		
HAX1	T1: CAGTGGTCCATTCGCAGACGGGG<br>T2: AAAGGTTCGCGTCCCAGTACGGG	
CLPB	T1: CGGACTGGGCATGTGCGCCCTGG<br>T2: GCGTCTGGAGATCATCGACAAGG	
HSP27-2E	T1: CAGCCGGCAACTCAGCAGCGGGG<br>T2: CCTACAGCCGCGCGCTCAGCCGG<br>HR template:<br>CGTGCGCCCCCTGCCCCCCGCCGCCATCGAGAGCCCCGCAGTGGCCGCGCCCGCCTACAGCCGCGCGCTCGAGAGACAACTCGAGAGCGGGGTCTCGGAGATCCGGCACACTGCGGACCGCTGGCGCGTGTCCCTGGATGTCAACCACTTCGCCC	

Supplemental Table 6. Antibodies used in this study

Antibody	Source	Identifier
Alexa Fluor 488 goat anti mouse	Invitrogen	cat# A-11001

Alexa Fluor 633 goat anti mouse	Invitrogen	cat# A-21052
Alexa Fluor 633 goat anti rabbit	Invitrogen	cat# A-21070
Alexa Fluor 680 goat anti rabbit	Invitrogen	cat# A-21109
mouse monoclonal anti-Alpha Tubulin	ThermoFisher	cat# A11126
rabbit polyclonal antibody anti-CLPB	Proteintech	cat# 15743-1-AP
mouse monoclonal antibody anti-Flag	Sigma	cat# F7425-0.2MG
rabbit polyclonal antibody anti-Foxo3a	Cell signaling	cat# 2497
mouse polyclonal antibody anti-GAPDH	Santacruz	cat# sc32233
mouse monoclonal antibody anti-HAX1	BD biosciences	cat# 610824
rabbit polyclonal antibody anti-HAX1	Proteintech	cat# 11266-1-AP
HRP conjugated antibody anti-GAPDH	Proteintech	cat# HRP-60004
HRP conjugated alpha Tubulin antibody	Proteintech	cat# HRP-66031
mouse monoclonal antibody anti-HSP27	Proteintech	cat# 66767-1-Ig
rabbit polyclonal antibody anti-HSP27	Proteintech	cat# 18284-1-AP
rabbit polyclonal antibody anti-LC3B	Cell signaling	cat# 2775S
rabbit polyclonal antibody anti-MITRAC12	Dr. Sven Dennerlein	Richter-Dennerlein R et al., 2016 (8)
mouse monoclonal antibody anti-OPA1	BD biosciences	cat# 612607
mouse monoclonal antibody anti-PGC1a	Proteintech	cat#66369-1-Ig

rabbit polyclonal antibody anti-phospho-HSP27(Ser82)	Cell signaling	cat# 2406S
rabbit polyclonal antibody anti-phospho-HSP27(Ser78)	Cell signaling	cat# 2405S
rabbit polyclonal antibody anti-Phospho-HSP27(Ser15)	Bioworld Technology	cat# AP0245
rabbit polyclonal antibody anti-PRKD2	Millipore	cat# 7-488
rabbit polyclonal antibody anti-phospho-PRKD2(Ser876)	Abnova	cat# PAB31649
mouse monoclonal antibody anti-SLP2	Proteintech	cat# 60052-1-Ig
rabbit polyclonal antibody anti-TFAM	Proteintech	cat#22586-1-AP
rabbit polyclonal antibody anti-Tim23	Dr. Sven Dennerlein	Richter-Dennerlein R et al., 2016 (8)
rabbit polyclonal antibody anti-Tim44	Proteintech	cat# 13859-1-AP
mouse monoclonal antibody anti-Tom20	Proteintech	cat# 66777-1-Ig
rabbit polyclonal antibody anti-Tom70	Dr. Sven Dennerlein	Richter-Dennerlein R et al., 2016 (8)
APC anti-human CD33	Biolegend	cat# 303408
FITC anti-human CD16	Beckman Coulter	cat# IM0814U
Fixable Viability Stain 780	BD biosciences	cat# 565388
PE anti-human CD49d	Biolegend	cat# 304304
PE-Cy7 anti-human CD11b	Biolegend	cat# 301322
PE Cy7 anti-human CD33	Biolegend	cat# 366618

Supplemental Table 7. Chemicals, Peptides and Recombinant Proteins used in this study

Reagent	Source	Identifier
Anti-FLAG M2 Affinity Gel	Sigma-Aldrich	A2220
L-Arginine HCl-^{13}C	Silantes	201204102

4,4,5,5-D4-L-Lysine 2HCl-^{2}H	Silantes	211104113
L-Arginine HCl-^{13}C,^{15}N	Silantes	201603902
L-Lysine HCl-^{13}C, ^{15}N	Silantes	211603902
MitoSOX Red Mitochondrial Superoxide indicator	Thermo Fisher Scientific	M36008
CRT0066101 hydrochloride	Sigma-Aldrich	SML1507
Doxycycline hyclate	Sigma-Aldrich	D9891
B18R	Stemgent	cat# 03-0017
BMP4	Peprotech	cat# 120-05
VEGF	Peprotech	cat# 100-20
CHIR99021	millipore	cat# 361571
bFGF	Peprotech	cat# 100-18B
SCF	Peprotech	cat# 300-07
SB431542	selleckchem	cat# S1067
IL-3	Peprotech	cat# 200-03
Flt-3	Peprotech	cat# 300-19
TPO	Peprotech	cat# 300-18
G-CSF	Peprotech	cat# 300-23
Oligomycin	Sigma-Aldrich	O4876
FCCP	Sigma-Aldrich	C2920
Rotenone	Sigma-Aldrich	R8875
Antimycin A	Sigma-Aldrich	A8674
Hoechst 33342 solution	Thermo Fisher Scientific	62249

Supplemental Table 8. Cell lines used in this study

Cell Lines	Source
HeLa	DSMZ
HEK293T	DSMZ
PLB-985	DSMZ
iPSCs	Kunze C et al., 2017 (9)
iPSCs- WT (the scramble)	This study
iPSCs- HAX1$^{-/-}$	This study
iPSCs- HAX1$^{-/-}$+HAX1	This study
iPSCs- HAX1$^{-/-}$+HSP27	This study

iPSCs- HAX1$^{-/-HSP27_2E}$	This study
HeLa- HAX1$^{-/-}$	This study
HeLa-CLPB$^{-/-}$	This study
PLB-pRRL-IRES-EGFP	This study
PLB-HAX1$^{-/-}$	This study
PLB-HAX1$^{-/-}$+pRRL-HAX1-IRES-EGFP	This study
PLB-HAX1$^{-/-}$+pRRL-HSP27^{FLAG}-IRES-EGFP	This study
PLB-CLPB$^{-/-}$	This study
PLB-CLPB$^{-/-}$+pRRL-CLPB-IRES-EGFP	This study
PLB-TRMPVIR-shNC	This study
PLB-TRMPVIR-shPRKD2	This study

Supplemental Table 9. Constructions used in this study

**Constructs**	**Source**	**Identifier**
pRRL-IRES-EGFP	Schambach, A., et al., 2006 (10)	PMID: 16226060
pRRL-IRES-RFP	Schambach, A., et al., 2006 (10)	PMID: 16226060
pSpCas9-2A-GFP (PX458)	Addgene	cat# 48138
pDONR233-PRKD2	Addgene	cat# 23490
TRMPVIR	Addgene	cat# 27994
pENTR1A no cdDB	Addgene	cat# 17398
PB-TAC-ERP2	Addgene	cat# 80478

Supplemental Table 10. Software and Algorithms used in this study

**Software and Algorithms**	**Source**	**Identifier**
Excel	Microsoft	
Prism	GraphPad	
Zen 2.3	Zeiss	
Gen5 1.11.5	BioTek	
(http://crispr.mit.edu/)	Feng Zhang's Lab	

FlowJo software	Tree Star Software	
Molecular Imager Chemi Doc	Biorad	
SprayQC	Scheltema RA et al., 2012	PMID: 22515319
MaxQuant (version 1.6.1.13)	Cox J et al., 2008	PMID: 19029910
Perseus (version 1.5.4.2)	Tyanova S et al., 2016	PMID: 27348712
Genome Analysis Toolkit (GATK) (version 3.5/3.6)	Broad Institute	https://software.broadinstitute.org/gatk/
Ensembl Variant Effect Predictor (VEP) (version 83/89)	McLaren et al., 2016	PMID: 27268795
Combined Annotation Dependent Depletion (CADD) (version 1.3)	Kircher et al., 2014	PMID: 24487276
ProtParam	Expasy 3.0	
gnomAD (version 2.1.1)	gnomAD	https://gnomad.broadinstitute.org/
Wave (version 2.6.3)	Agilent Technologies	https://www.agilent.com

Supplemental references

1. Chae YC, Angelin A, Lisanti S, Kossenkov AV, Speicher KD, Wang H, et al. Landscape of the mitochondrial Hsp90 metabolome in tumours. *Nature Communications.* 2013;4:2139.
2. Schulz C, Lytovchenko O, Melin J, Chacinska A, Guiard B, Neumann P, et al. Tim50's presequence receptor domain is essential for signal driven transport across the TIM23 complex. *J Cell Biol.* 2011;195(4):643-56.
3. Boisvert FM, Ahmad Y, Gierlinski M, Charriere F, Lamont D, Scott M, et al. A quantitative spatial proteomics analysis of proteome turnover in human cells. *Mol Cell Proteomics.* 2012;11(3):M111 011429.
4. Scheltema RA, and Mann M. SprayQc: a real-time LC-MS/MS quality monitoring system to maximize uptime using off the shelf components. *J Proteome Res.* 2012;11(6):3458-66.
5. Cox J, and Mann M. MaxQuant enables high peptide identification rates, individualized p.p.b.-range mass accuracies and proteome-wide protein quantification. *Nat Biotechnol.* 2008;26(12):1367-72.
6. Cox J, and Mann M. Quantitative, high-resolution proteomics for data-driven systems biology. *Annu Rev Biochem.* 2011;80:273-99.
7. Tyanova S, Temu T, and Cox J. The MaxQuant computational platform for mass spectrometry-based shotgun proteomics. *Nat Protoc.* 2016;11(12):2301-19.
8. Richter-Dennerlein R, Oeljeklaus S, Lorenzi I, Ronsor C, Bareth B, Schendzielorz AB, et al. Mitochondrial Protein Synthesis Adapts to Influx of Nuclear-Encoded Protein. *Cell.* 2016;167(2):471-83 e10.
9. Kunze C, Borner K, Kienle E, Orschmann T, Rusha E, Schneider M, et al. Synthetic AAV/CRISPR vectors for blocking HIV-1 expression in persistently infected astrocytes. *Glia.* 2018;66(2):413-27.
10. Schambach A, Bohne J, Baum C, Hermann FG, Egerer L, von Laer D, et al. Woodchuck hepatitis virus post-transcriptional regulatory element deleted from X protein and promoter sequences enhances retroviral vector titer and expression. *Gene Ther.* 2006;13(7):641-5.

unedited blots for Figure 1

Figure 1B

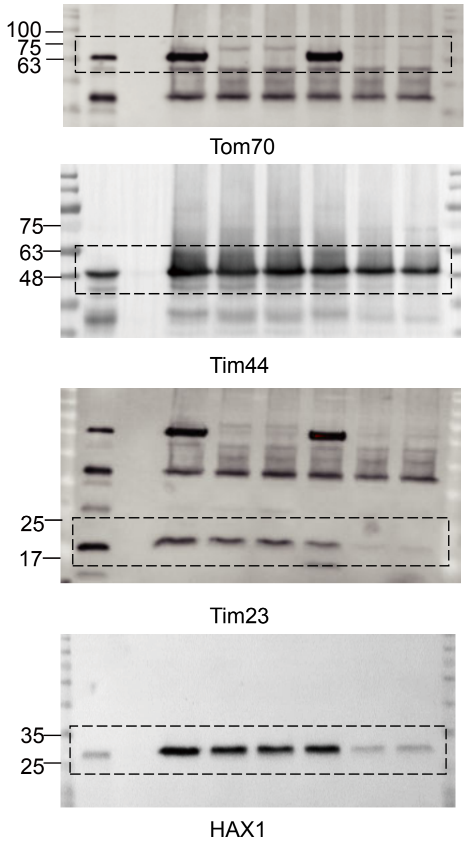

Figure 1D

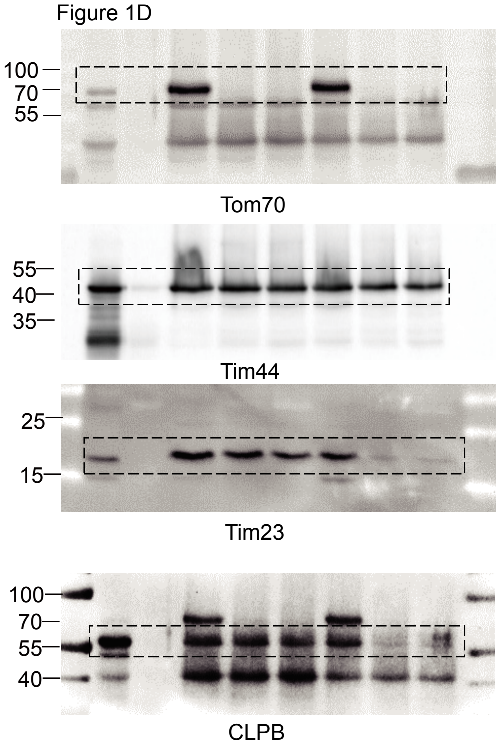

Figure 1E

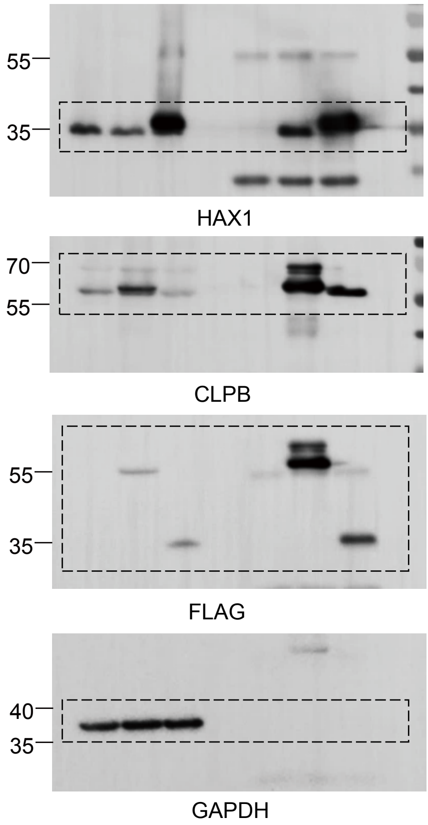

Figure 1F

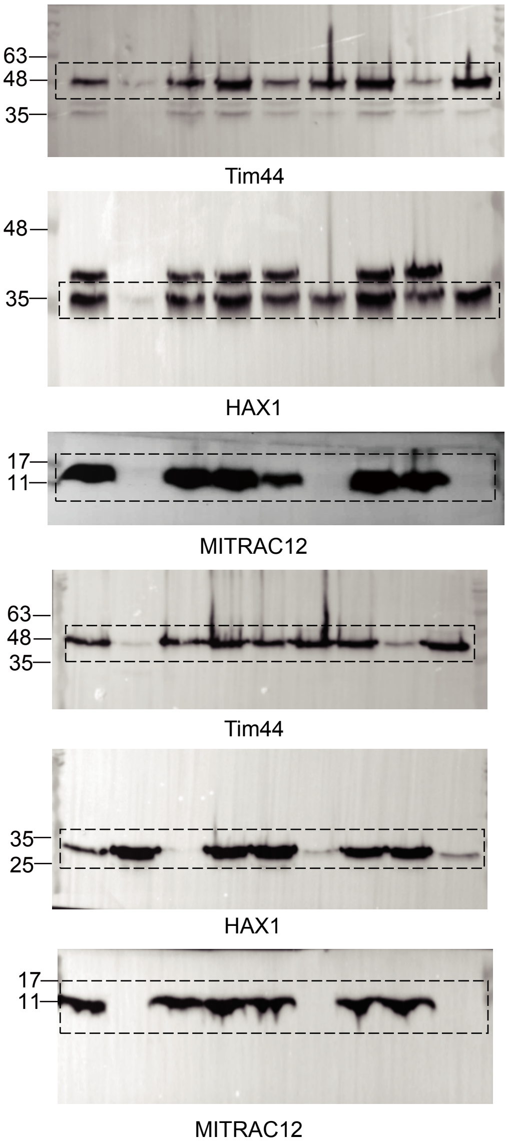

unedited blots for Figure 2

Figure 2B

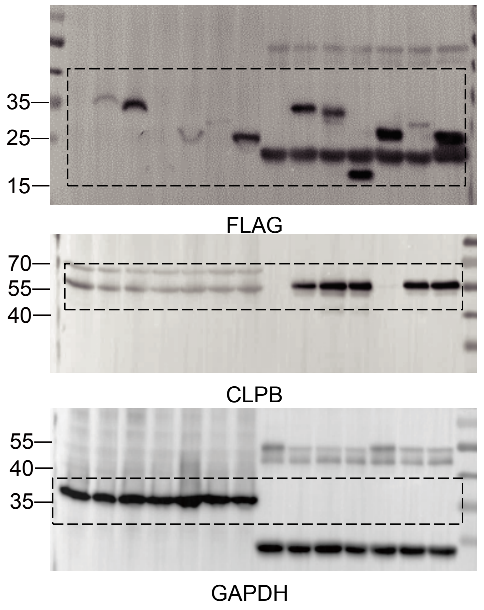

Figure 2C

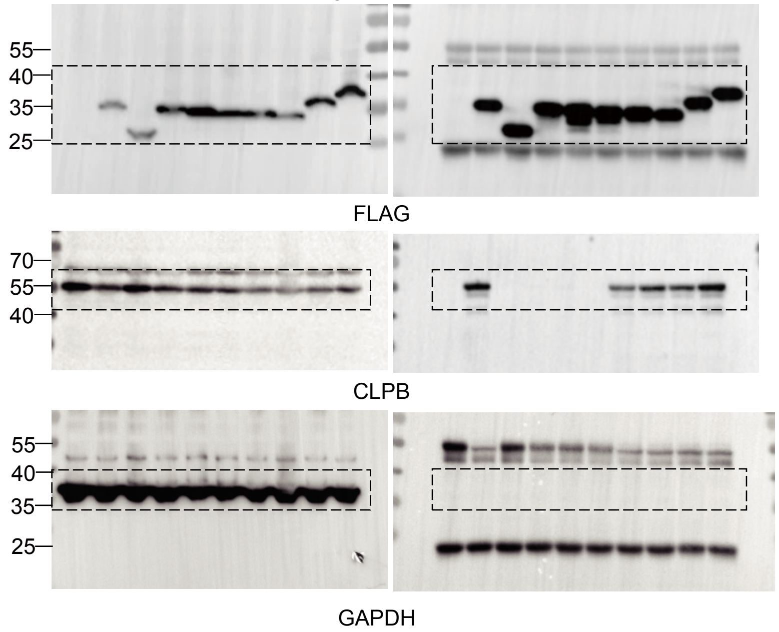

Figure 2D

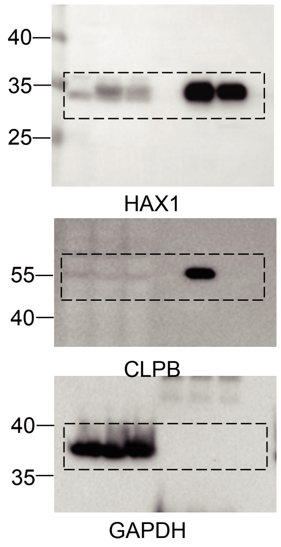

Figure 2F

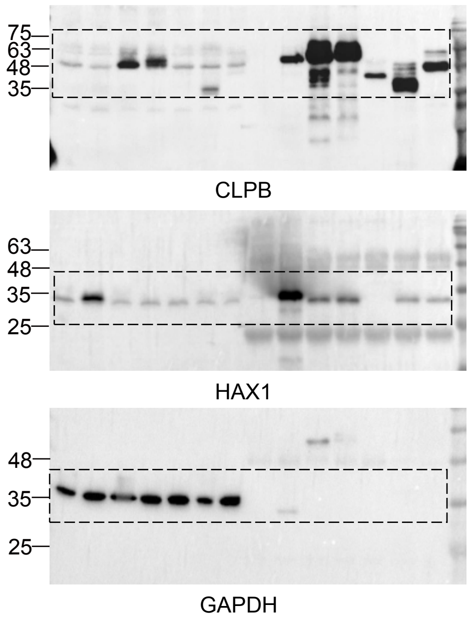

Figure 2G

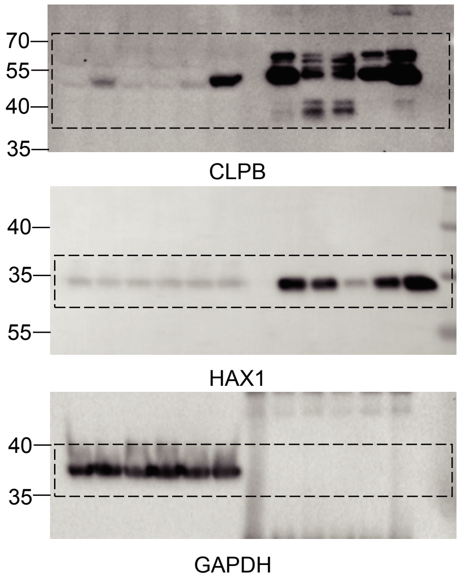

unedited blots for Figure 4

Figure 4A

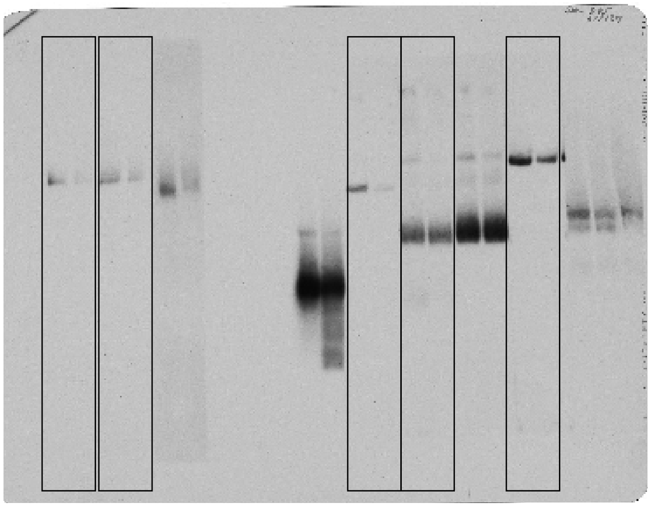

higher exposure time

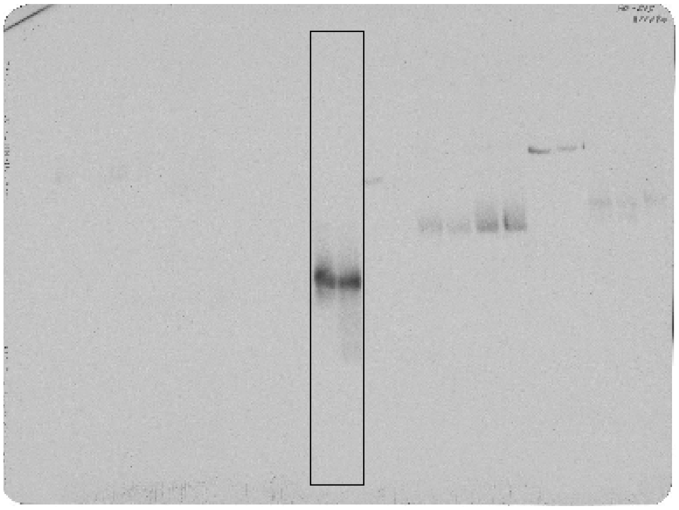

lower exposure time

unedited blots for Figure 5

Figure 5B

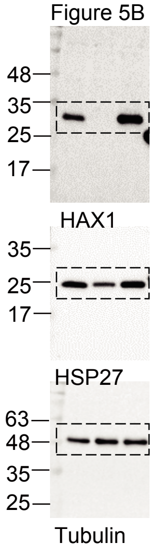

Figure 5C

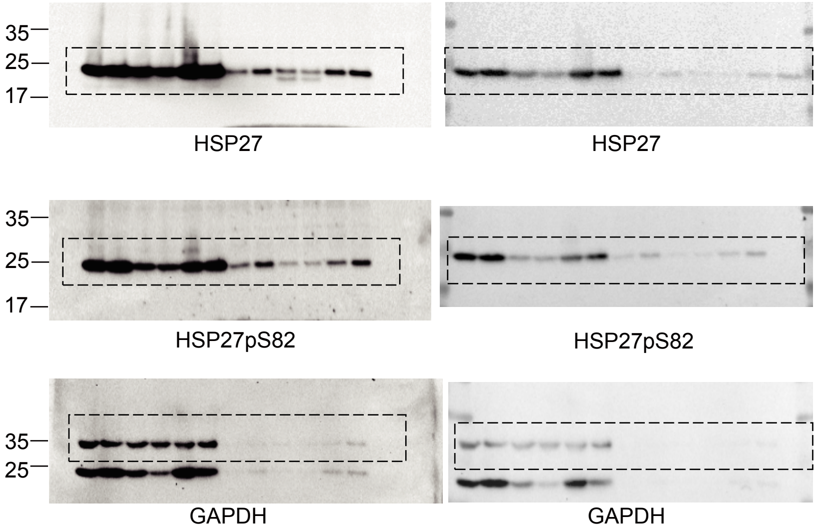

Figure 5D

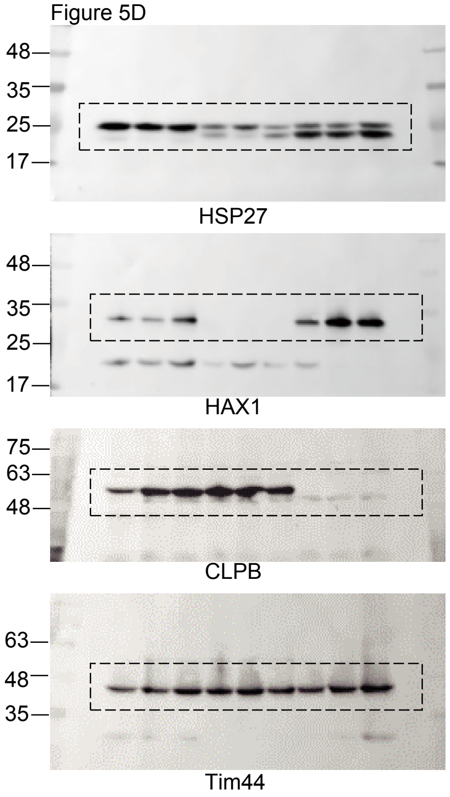

Figure 5E

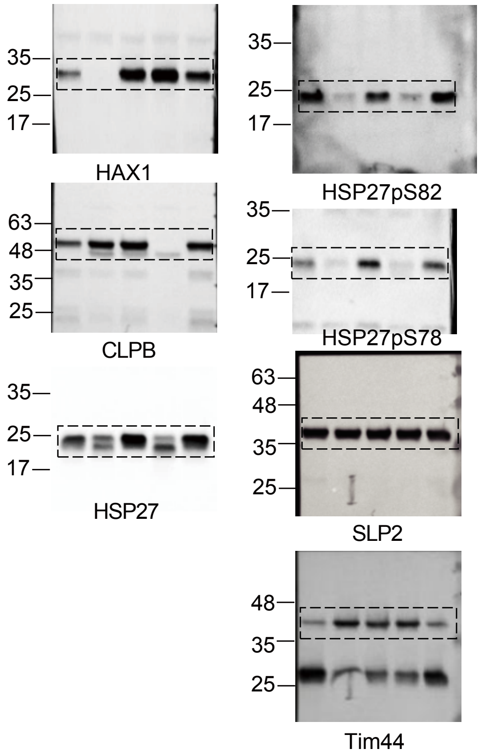

Figure 5F

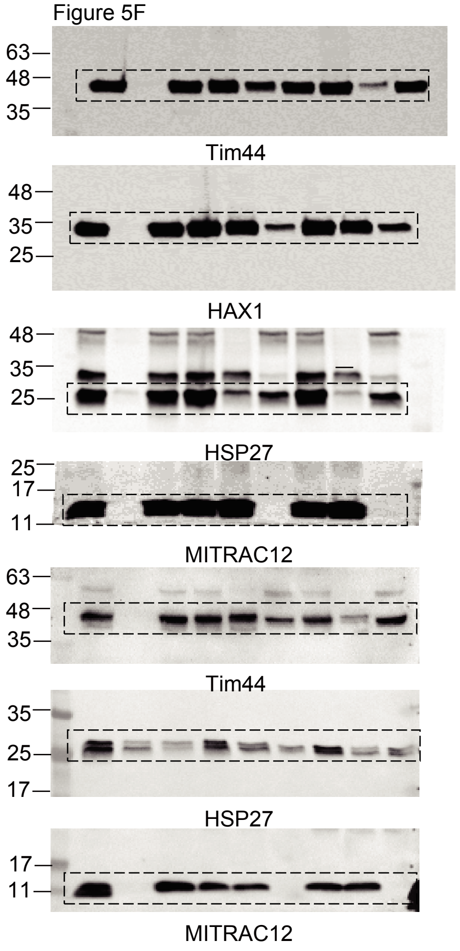

Figure 5F

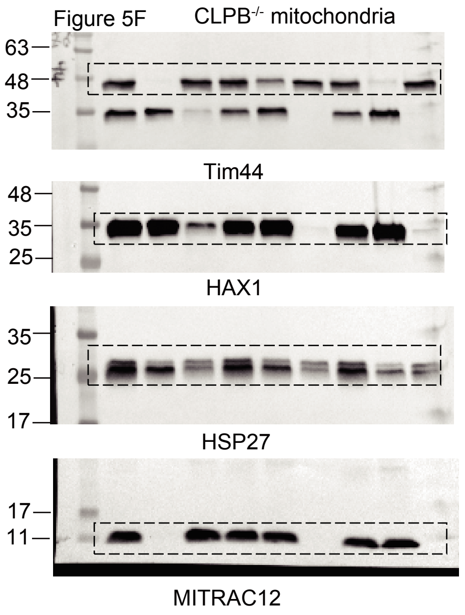

unedited blots for Figure 6

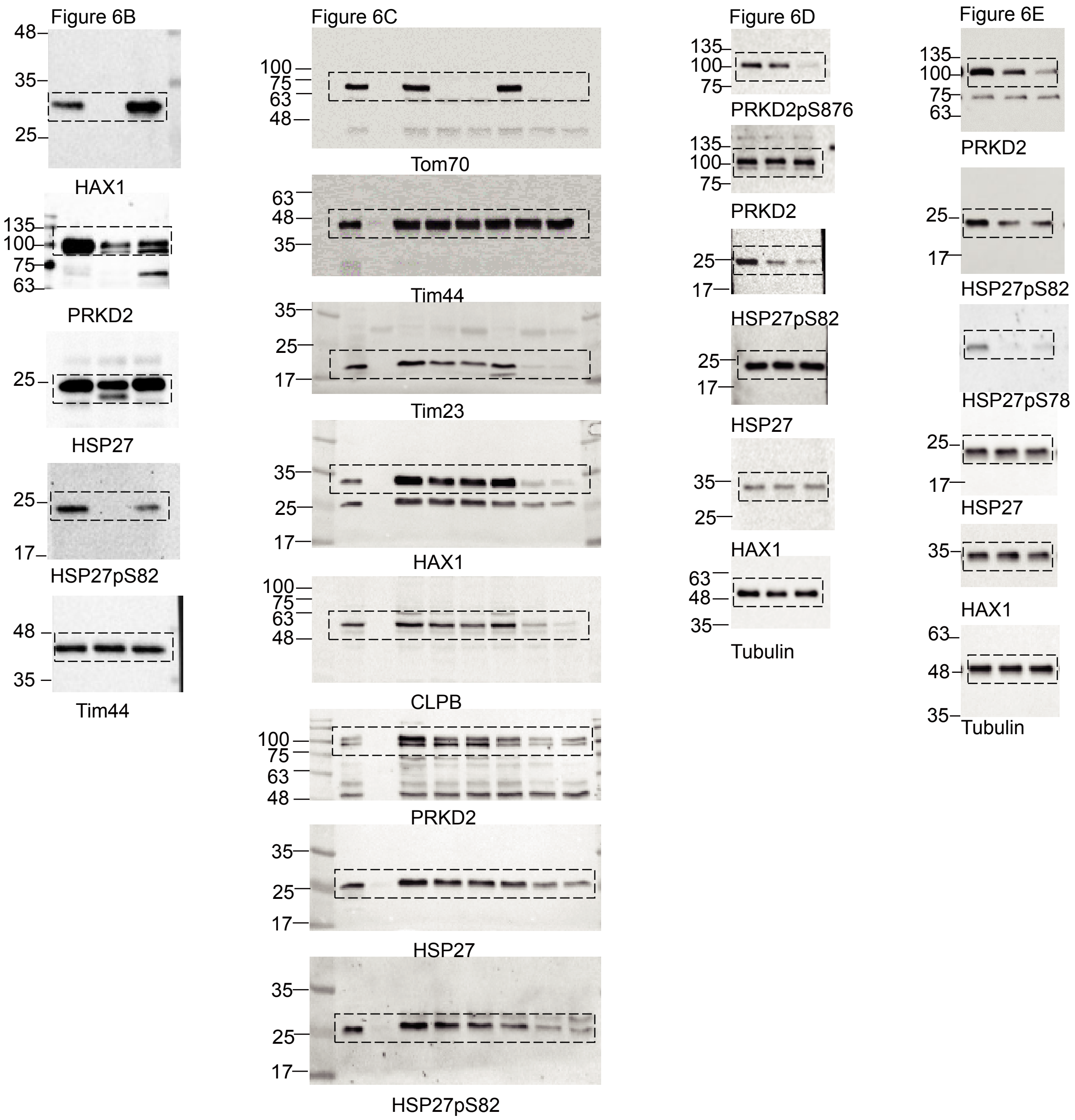

unedited blots for Figure 7

Figure 7A

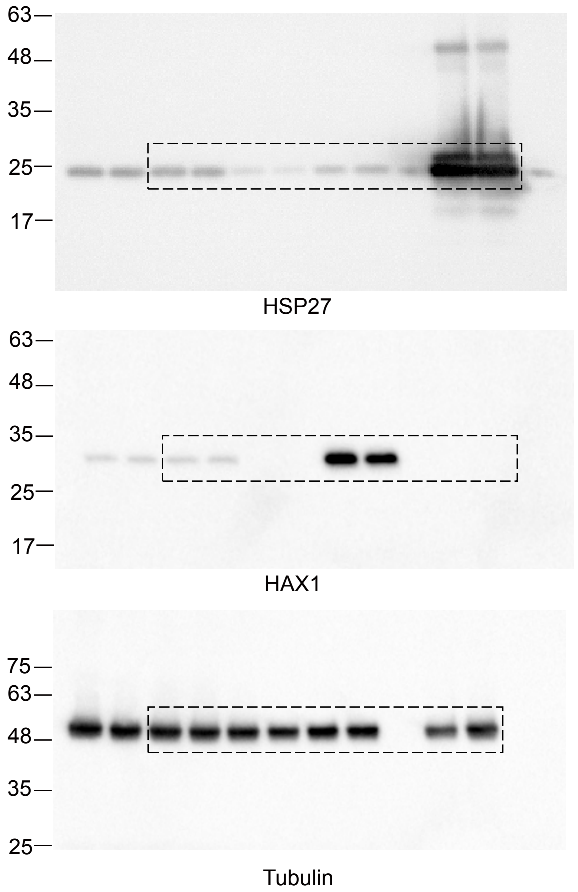

unedited blots for Supplemental Figure 1

Supplemental Figure 1A

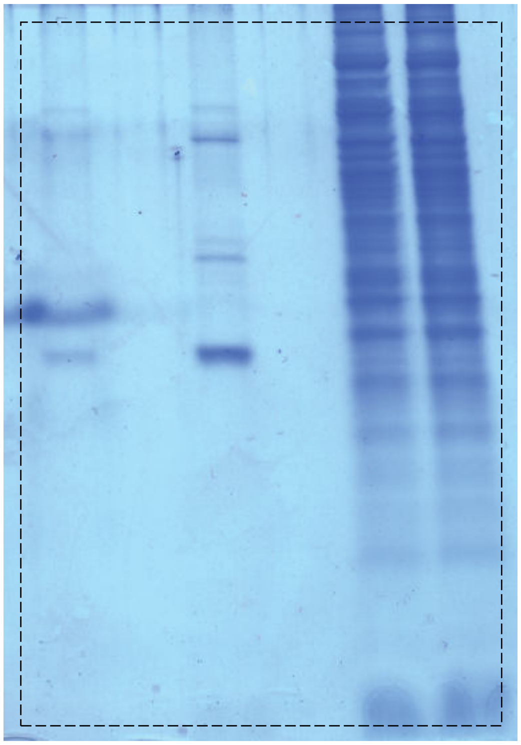

Supplemental Figure 1B

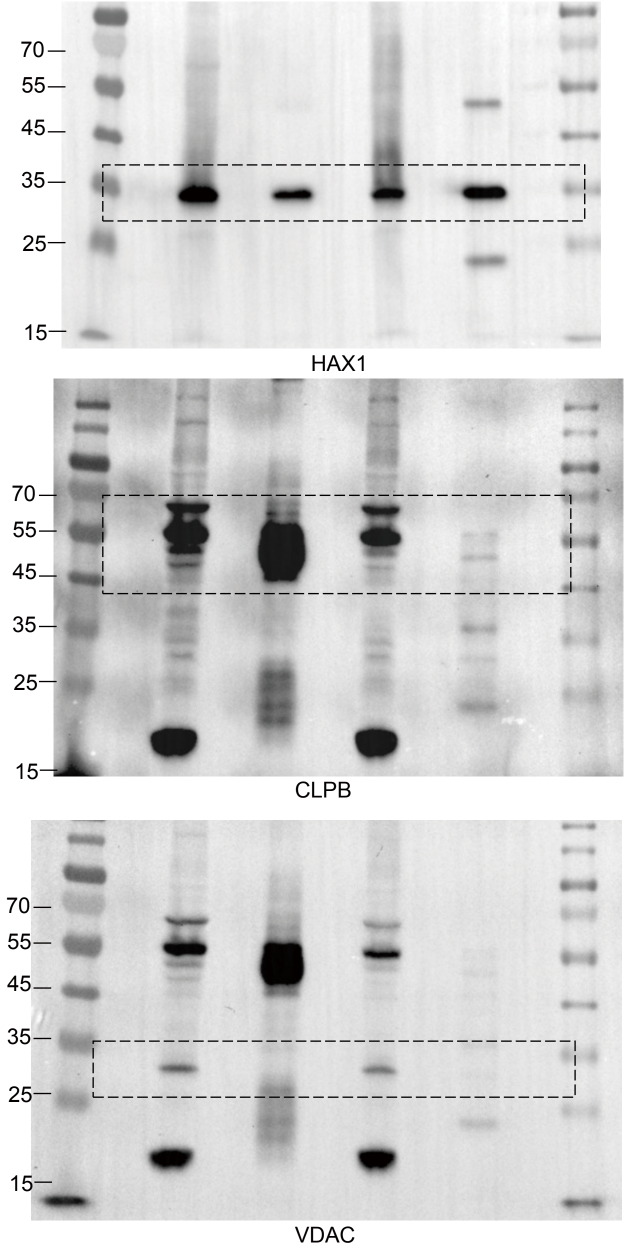

unedited blots for Supplemental Figure 2

Supplemental Figure 2A

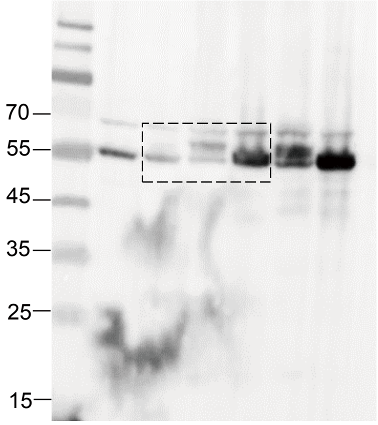

CLPB

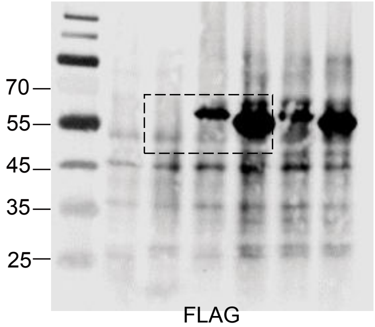

FLAG

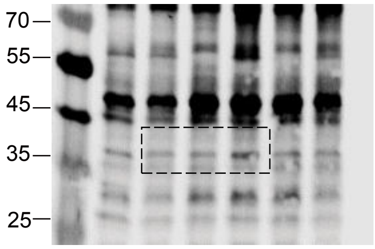

RFP

Supplemental Figure 2B

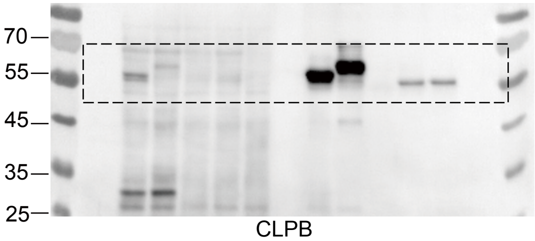

CLPB

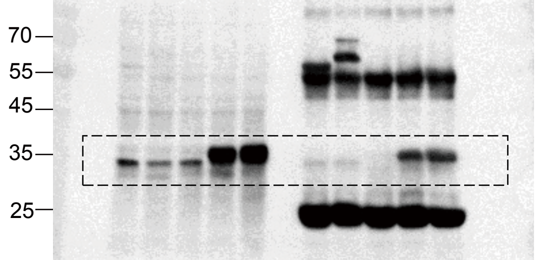

FLAG

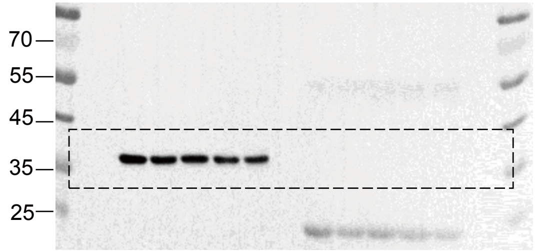

GAPDH

Supplemental Figure 2C

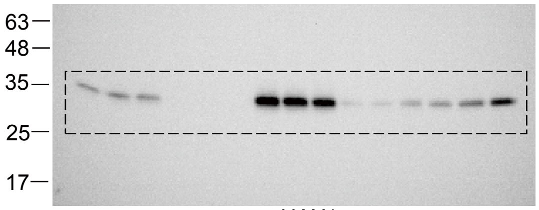

HAX1

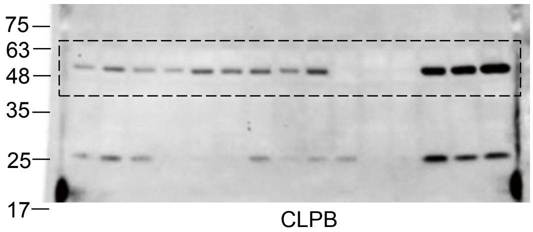

CLPB

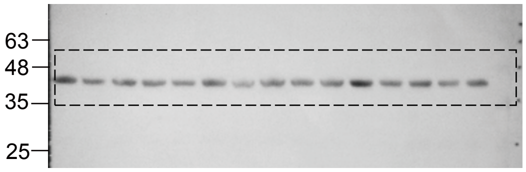

β-Actin

unedited blots for Supplemental Figure 3

Supplemental Figure 3E

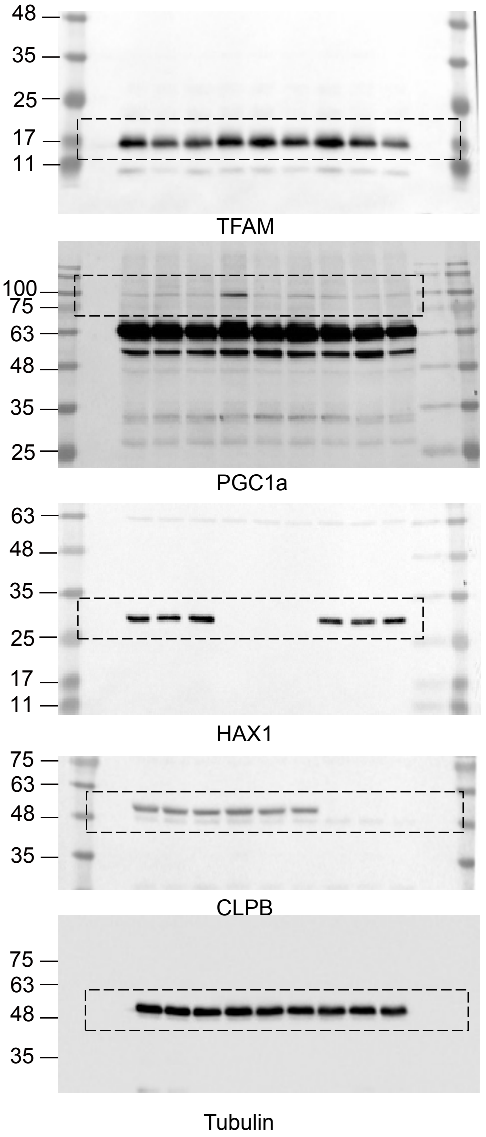

unedited blots for Supplemental Figure 4

Supplemental Figure 4A upper panel

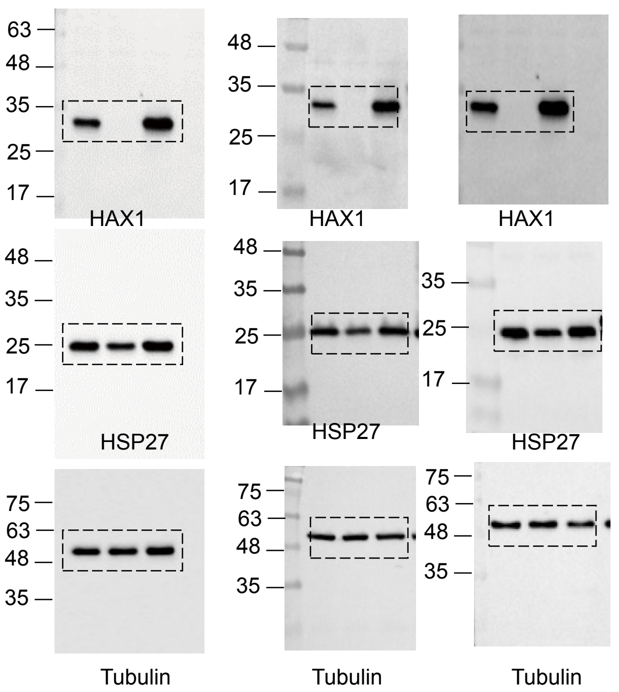

Supplemental Figure 4A lower panel

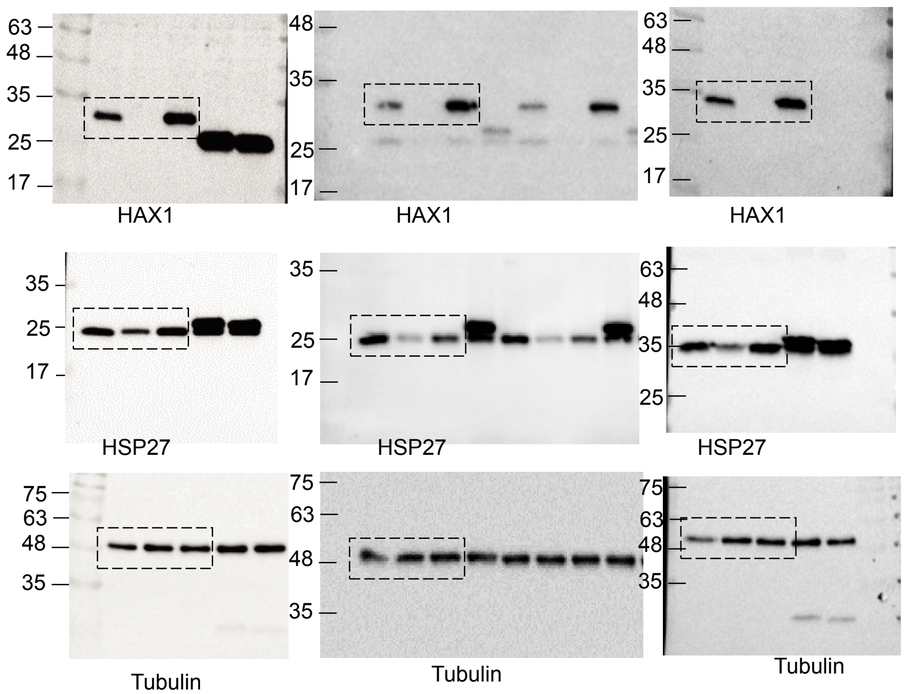

Supplemental Figure 4B

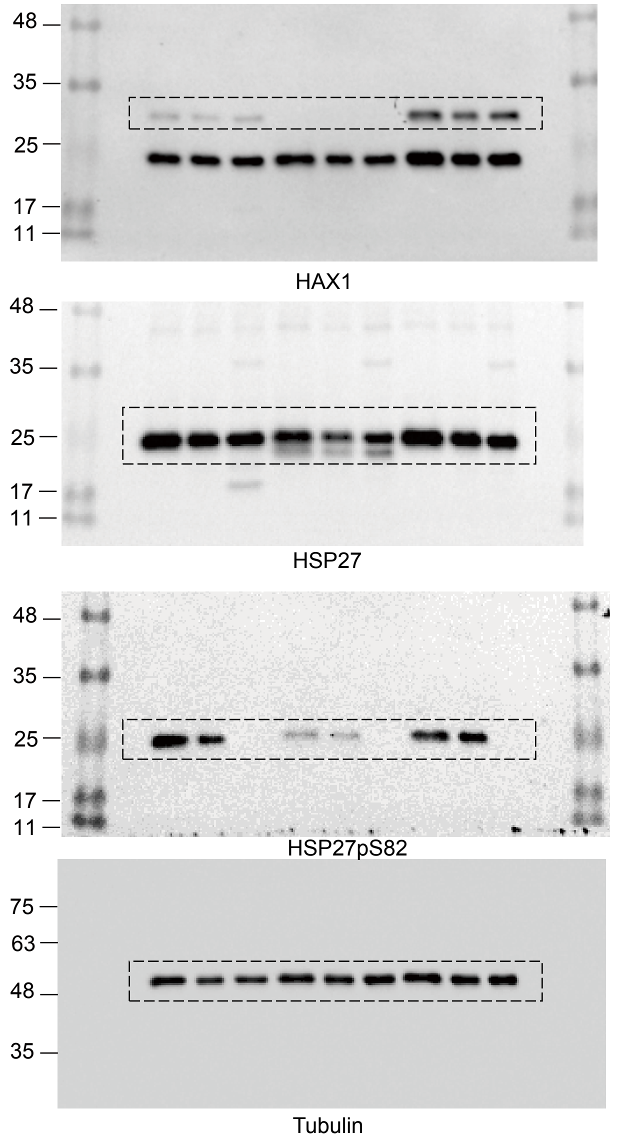

unedited blots for Supplemental Figure 5

Supplemental Figure 5O

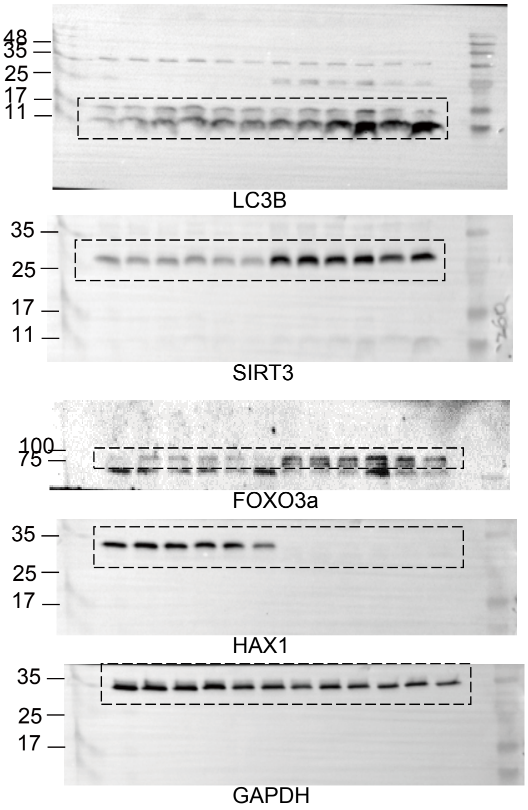

Supplemental Figure 5P

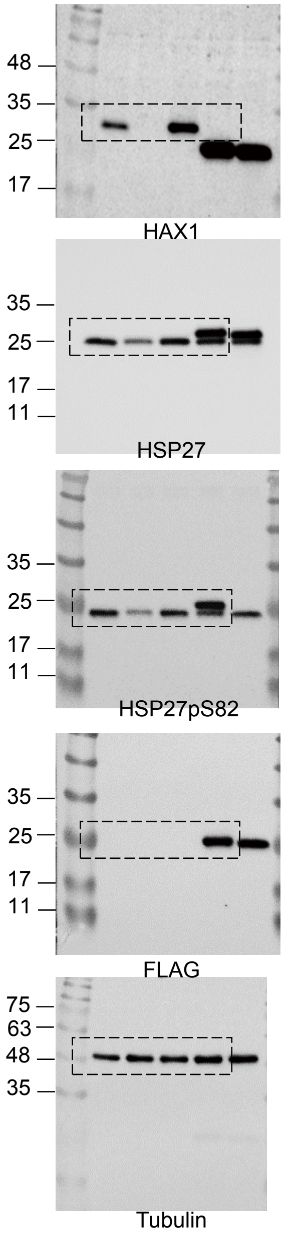

unedited blots for Supplemental Figure 6

Supplemental Figure 6E

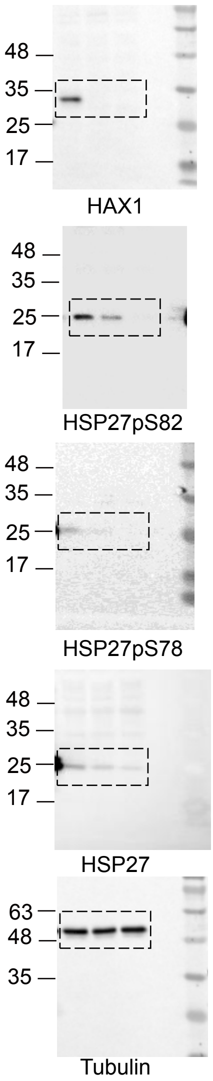

Supplement: Supplemental data [file jci-132-153153-s112.pdf]
